# Efficacy and safety of immunochemotherapy, immunotherapy, chemotherapy, and targeted therapy as first-line treatment for advanced and metastatic esophageal cancer: a systematic review and network meta-analysis

**DOI:** 10.1016/j.lanwpc.2023.100841

**Published:** 2023-07-06

**Authors:** Zhen Gao, Shujie Huang, Sichao Wang, Dezhao Tang, Wei Xu, Ruijie Zeng, Guibin Qiao

**Affiliations:** a Department of Thoracic Surgery, Guangdong Provincial People's Hospital (Guangdong Academy of Medical Sciences), Southern Medical University, Guangzhou, China; b Centre of Cancer Cell and Molecular Biology, Barts Cancer Institute, Queen Mary University of London, John Vane Science Centre, Charterhouse Square, London, UK; c Department of Clinical Oncology, Li Ka Shing Faculty of Medicine, The University of Hong Kong, Hong Kong, China; d Department of Clinical Oncology, The University of Hong Kong-Shenzhen Hospital, Shenzhen, China; e School of Public Health, Chongqing Medical University, Chongqing, China; f Department of Gastroenterology, Guangdong Provincial People's Hospital (Guangdong Academy of Medical Sciences), Southern Medical University, Guangzhou, China

**Keywords:** Esophageal carcinoma, Immunotherapy, Chemotherapy, PD-L1, Metastasis, Overall survival

## Abstract

**Background:**

The treatment of esophageal cancer has entered a new phase with the development of immunotherapy. The current investigation purpose is to investigate and contrast the efficacy and safety of immunotherapy, immunochemotherapy, chemotherapy, and targeted therapy as first-line treatment for individuals suffering from advanced and metastatic esophageal cancer.

**Methods:**

Within the framework of this systematic review and network meta-analysis, clinical trials published or reported in English up until 01 May, 2022, were retrieved from Embase, PubMed, Cochrane Central Register of Controlled Trials, the ClinicalTrials.gov databases, ESMO, and ASCO. The analysis incorporated randomized controlled trials (RCTs) from phase 2 to 3 that evaluated a minimum of two first-line therapeutic regimens for metastatic esophageal cancer were included in the analysis. The primary outcomes were overall survival (OS) and progression-free survival (PFS). Secondary clinical outcomes included the incidence of objective response rate (ORR), and adverse events (AEs) of any grade and ≥3 grade. Relative summary data were extracted from included studies by GZ, HS, WS, and TD. For clear statistical analysis, chemotherapy was divided into two categories of fluorouracil-based chemotherapy (FbCT) and fluorouracil-free chemotherapy (FfCT). Bayesian frequentist approach was employed to conduct the network meta-analysis. The indirect intercomparison between regimens was presented with league tables (HRs and 95% CI for OS and PFS, ORs and 95% CI for ORR and AEs). A greater surface value under the cumulative ranking (SUCRA) indicates a higher potential ranking for the corresponding treatment. A further calculation of relative results about esophageal squamous cell cancer was performed in the subgroup analysis. The current protocol for the systematic review has been properly registered on PROSPERO (registration number: CRD42021241145).

**Findings:**

The final analysis comprised 17 trials that involved 9128 patients and 19 distinct treatment regimens. Within the scope of investigated immunotherapy (IO) combinations, toripalimab + FfCT (tori + FfCT) demonstrated the best OS advantages (tori + FfCT vs. FbCT, HR 0.57, 95% CI 0.38–0.85; tori + FfCT vs. FfCT, HR 0.58, 95% CI 0.43–0.78). In terms of PFS, camrelizumab + FfCT (cam + FfCT) demonstrated the best PFS advantages (FbCT vs. cam + FfCT, HR 1.79, 95% CI 1.22–2.63; FfCT vs. cam + FfCT, HR 1.79, 95% CI 1.47–2.17). Nivolumab + FbCT (nivo + FbCT vs. FfCT, OR 3.29, 95% CI 1.43–7.56) showed the best objective responses. Compared to the conventional chemotherapy regimen, the toxicity was observed to be the slightest for the tori + FfCT (FbCT vs. tori + FfCT, OR 3.07, 95% CI 1.22–7.7) and sintilimab + FfCT (FbCT vs. sin + FfCT, OR 2.93, 95% CI 1.16–7.37). The results in this study were evaluated as having a low heterogeneity since the I^2^ value was ≤25% in all analyses.

**Interpretation:**

Compared to foreign IO combinations, sin + FfCT, tori + FfCT, cam + FfCT, and tisle + FbCT are superior first-line treatment options for patients with advanced and metastatic esophageal cancer. Although foreign IO combinations, such as pembro + FbCT and nivo + FbCT obtained better objective response rates than other IO combinations, the addition of chemotherapy to IO worsens the safety profiles. Our findings could provide complementary evidence for current guideline recommendations.

**Funding:**

This work was supported by a grant from the Science and Technology Program of Guangzhou, China (202206010103); and 10.13039/501100003453Natural Science Foundation of Guangdong Province (2022A1515012469).

**Research in context**

**Evidence before this study**

After decades of domination of chemotherapy followed by an unsatisfied challenge from targeted therapy, the efficacy and safety of immune checkpoint inhibitors (ICIs) plus chemotherapy as first-line treatment for esophageal cancer (EC) have been reported. However, no head-to-head comparison between targeted therapy regimens or immunochemotherapy regimes has been published. Consequently, there is an increasing need for first-class evidence about the optimal choice for advanced and metastatic EC. Several meta-analysis have also corroborated the advantangeous effect on survival outcomes associated with immunochemotherapy compared to chemotherapy alone. However, no study has compared the efficacy and safety of immunochemotherapy, immunotherapy, chemotherapy, and targeted therapy for EC patients. Current meta-analyses ignored the difference between chemotherapy, although the difference is relatively small. The optimal choice of drug or treatment regimen for esophageal cancer patients as a first-line therapy remains controversial. Based on the PRISMA guideline of network meta-analysis (Supplementary File S1), we searched studies that were published in PubMed, EMBASE, Cochrane Central Register of Controlled Trials, and ClinicalTrials.gov databases before 1 May 2022, with search terms including “metastatic esophageal or gastroesophageal junction cancer”, “immunotherapy”, “PD-1 inhibitor”, “CTLA-4” with a restriction of randomized controlled trials (RCTs) (Supplementary File S2). Adittionaly, unofficially published trials from prominent international conferences (ASCO, ESMO) published between 2018 and 2022 were manually included.

**Added value of this study**

We compared 19 treatment regimens especially ICIs combined with different chemotherapy regimens (with or without 5-FU). It is the first to include all valuable treatment regimens, including target therapy in a network meta-analysis for advanced and metastatic EC. The findings of our investigation indicate that the employment of IO combinations yielded superior findings towards both OS and PFS compared to conventional chemotherapy and chemotherapy-targeted combined therapy among EC patients. Furthermore, compared to foreign IO combinations, sintilimab + chemotherapy, toripalimab + chemotherapy, camrelizumab + chemotherapy, and tislelizumab + chemotherapy were superior first-line treatment options for patients with advanced and metastatic EC.

**Implications of all the available evidence**

Our results showed a possibility of changing the recommendation of current EC treatment. More specific drugs or regimens should be administered on the basis of the current conclusion. The indirect comparison could contain unnoticed bias or omission in guiding clinical practise. Further head-to-head clinical trials should be performed to increase the reliability of current results.

## Introduction

Esophageal cancer (EC) is the sixth leading cause of cancer death and caused 540,000 deaths in 2020 alone.^1^ Owing to the scarcity of efficient early screening measures, patients initially diagnosed with EC often present with advanced or metastatic diseases.^2^^,^^3^

In the era of chemotherapy, advanced EC shows a dismal prognosis with a 5-year OS rate of ≤10%.^4^ 5-fluorouracil-based chemotherapy (FbCT) for treating advanced and metastatic EC is accepted worldwide.^5^ The efficacy and safety of various chemotherapy strategies for EC were evaluated through single-arm phase II trials. The outcomes revealed that the median OS duration was about 10 months for EC patients.^6^, ^7^, ^8^ Similar to chemotherapy, little clinical benefit was discovered in targeted therapy.^9^ Several clinical trials evaluated the survival and safety benefits of these novel drugs over traditional chemotherapy; however, their results showed only slightly improved survival time and more adverse events (AEs).^10^, ^11^, ^12^ The efficacy of chemoradiotherapy as an adjunctive therapy to surgery in patients who have been diagnosed with resectable locally advanced resectable esophageal cancer was validated with respect to OS benefits.^13^^,^^14^ However, concurrent chemoradiotherapy is currently not recommended as first-line treatment option for metastatic EC.^15^

Immunotherapy has recently been widely applied in EC treatment.^16^, ^17^, ^18^, ^19^, ^20^, ^21^, ^22^, ^23^ ICIs are a class of monoclonal antibodies (mAbs) that maintain immune tolerance and prevent cancer cell evasion from the immune response by improving immune surveillance.^24^ The most popular ICIs targets include programmed cell death receptor-1 (PD-1), programmed cell death ligand-1 (PD-L1), and cytotoxic T lymphocyte-associated molecule-4 (CTLA-4). CTLA-4 is considered one of the immunoglobulin superfamily constituents on the T cell surface. It is closely related to the T-cell costimulatory receptor, CD28. CTLA-4 and CD28 both interact with the same ligands, B7-1 (CD80) and B7-2 (CD86), which are situated on the antigen-presenting cells (APCs) surface. Studies revealed that CTLA-4 performs a critical function in regulating the immune response by reducing T-cell activation. Upon interaction with its ligands, CTLA-4 sends inhibitory signals to T cells, effectively limiting their activation, proliferation, and cytokine production.^25^ Ipilimumab was the first CTLA-4 inhibitor that was approved for the cancer treatment. It is also a component of the double immunotherapy arm which prolonged the OS and increased the complete response rate compared to chemotherapy in the CheckMate-648 trial.^23^ Cadonilimab, a PD-1/CTLA-4 double inhibitor, also got good results in gastric or gastroesophageal junction cancer.^26^ PD-1 and PD-L1 is another molecule pair that contains T-cell-inhibition function. The PD-1 expression was detected on the T cells surface, and it binds with PD-L1, which is always over-expressed on tumour cell surface. ICIs based on PD-1 pathway blockade were proven to lead to tumor regression and to derive clinical benefit for tumour patients.^27^ ICIs-based combination regimens as first-line, neoadjuvant, and adjuvant settings have shown positive effects in extending the survival time of EC patients.^28^^,^^29^ Recently, there have been several randomized controlled trials (RCTs) conducted to assess the relative efficacy of immunotherapy in combination with chemotherapy contrasted to placebo in conjunction with chemotherapy as first-line treatment for EC patients.^19^^,^^22^ These studies suggested that immunochemotherapy as compared to chemotherapy showed better OS in EC patients. Notably, the indications for ICIs within esophageal adenocarcinoma (AEC) and esophageal squamous cell carcinoma (SCC) may vary depending on the specific ICI and the patient's tumor characteristics. Clinical trial results supported in both SCC and AEC patients. However, the guidelines provide different recommendations for SCC and AEC. The Food and Drug Administration (FDA) and Chinese National Medical Products Administration (NMPA) have granted approval for the first-line treatment which involves the utilization of nivolumab in combination with fluoropyrimidine and platinum-containing chemotherapy for treating advanced unresectable or metastatic esophageal squamous cell carcinoma without regard for the PD-L1 expression, as per the findings of CheckMate-648.^15^^,^^23^ On the other hand, nivolumab in combination with fluoropyrimidine and oxaliplatin was defined to conditionally recommend for esophageal adenocarcinoma patients with a combined positive score (CPS) less than 5. It is noteworthy that the efficacy of immune checkpoint inhibitors (ICIs) in treating esophageal adenocarcinoma and esophageal squamous cell carcinoma is subject to variability based on the specific tumour attributes and immune profile of the patients. The ongoing research aims to optimize the use of ICIs in both subtypes and identify biomarkers that have the potential to predict the response to the mentioned therapies. Moreover, several meta-analyses have corroborated the survival advantage of immunochemotherapy compared to chemotherapy alone for individuals with esophageal squamous cell cancer (ESCC).^30^, ^31^, ^32^ The optimal choice of drug or treatment regimen for esophageal cancer patients as a first-line therapy remained controversial.

The present study incorporated a systematic review and network meta-analysis, which entailed meticulous literature screening and rigorous statistical analysis for the purpose of identifying the optimal first-line option for advanced and metastatic EC patients.

## Methods

The current network meta-analysis (NMA) was carried out in accordance with the Preferred Reporting Items for Systematic Reviews and Meta-Analysis extension statement (PRISMA) (Supplementary File S1).^33^ The Bayesian approach enables the indirect comparison of treatment results between various treatment regimens that were not elucidated directly throughout the trials.^34^ This systematic review protocol was registered on PROSPERO (registration number: CRD42021241145).

### Data sources and search strategy

PubMed, EMBASE, Cochrane Central Register of Controlled Trials, and the ClinicalTrials.gov databases have been accessed to retrieve relevant English articles published till 01 May 2022. Moreover, to include updated outcomes, abstracts, posters, and presentations from prominent international conferences (American Society of Clinical Oncology, European Society of Medical Oncology) published between 2018 and 2022 were also included. The keywords for the literature search were displayed in Supplementary File S2.

### Selection criteria

Published and unpublished phase II/III RCTs that met the following criteria were included:

(1) Phase II or III studies that enrolled treatment-naïve adult patients with metastatic esophageal or gastroesophageal junction cancer
(2) Trials that compared any two or more distinct arms of first-line treatment for patients with any level of PD-L1 expression
(3) RCTs that used Immunotherapy combinations as first-line treatment settings
(4) Trials that reported on at least one of the following clinical outcome measures: OS, PFS, objective response rate (ORR), and/or serious adverse events (SAEs) defined as greater than or equal to grade 3 according to the National Cancer Institute Common Terminology Criteria for Adverse Events

The exclusion criteria were delineated as follows:

(1) RCTs with ambiguous clinical outcomes (e.g., unreported hazard ratio [HR] and 95% confidence interval [95% CI])
(2) RCTs in which immunotherapy and immunochemotherapy were used as neoadjuvant treatments.

To determine the eligibility, titles and abstracts have been screened prior to the evaluation of full texts.

### Data extraction and quality assessment

Four investigators (GZ, HS, WS, and TD) independently extracted the data according to the PRISMA guideline. Discrepancies were resolved via discussion with other researchers (XW, GZ, HS, WS, QG). The clinical outcomes extracted included overall survival (OS), progression-free survival (PFS), the incidence of objective response rate (ORR), and adverse events of any grade and grade no less than 3. In investigations that encompassed both esophageal cancer and gastric malignancy, data for esophageal cancer will be extracted alone if possible. For studies that were published only with Kaplan–Meier curves and no HR or 95% CI (like CALGB 80403 3^35^ and Bleiberg 1997^9^), the individual patient data (IPD) was extracted with the iKM tool reported by Liu et al.^36^

The Cochrane Risk of Bias Tool (2.0) for RCTs was employed to evaluate the quality of the included studies.^37^ We then classified these studies into the low, high or unclear risk of bias (moderate risk).

### Statistical analysis

The primary outcomes for this meta-analysis were OS and PFS, the secondary results include ORR and AEs over grade 3. Network plots of all available treatments from the included trials were plotted to compare and illustrate multiple treatment arms. The Bayesian approach was used to analyze synthesized data, and the fixed effects module was used for this meta-analysis. When networks are sparse, random-effects models may generate implausibly wide credible intervals from network meta-analysis estimates, it can be difficult to draw reliable conclusions about the treatment effects, even when the direct and indirect estimates are coherent. When this occurred, we either conducted a fixed-effect network meta-analysis or used the direct estimates as our best estimates of the treatment effects.^38^^,^^39^ Pooled HRs with 95% CIs were calculated for OS, PFS, 1-year OS, and 1-year PFS. Pooled odds ratios (ORs) with 95% CI for ORR, DCR, and >grade 3 AEs. The Bayesian network meta-analysis (NMA) was performed using the R statistical package gemtc, which uses the contrast-based model and provides effect size estimates for multiple comparisons.^40^ The function mtc. run was used to generate samples by using the Markov Chains Monte Carlo (MCMC) sampler. MCMC simulations were run using three chains with different initial values for 100,000 iterations. The convergence of the network models derived from the MCMC simulations was assessed using trace and density plots. We used non-informative priors for all parameters and assumed a common heterogeneity. Several comparisons with pairwise meta-analysis were performed to verify the robustness of this study. Furthermore, the ranking probability was calculated for all available treatment strategies. The ranking was presented by surface under the cumulative ranking (SUCRA). A higher SUCRA value means a higher ranking in long OS, PFS and high ORR with a lower ranking in AEs ≥3 grade.

I^2^ values have been utilized to estimate heterogeneity between investigations. An I^2^ value <25% was considered to have low heterogeneity. The utilization of node splitting technique was implemented in order to evaluate the statistical incongruity that exists between the direct and indirect evidence at the level of paired comparisons.^41^ A P value of ≤0.05 was considered statistically significant.

## Results

### Systematic review and characteristics of the included studies

**Fig. 1:**
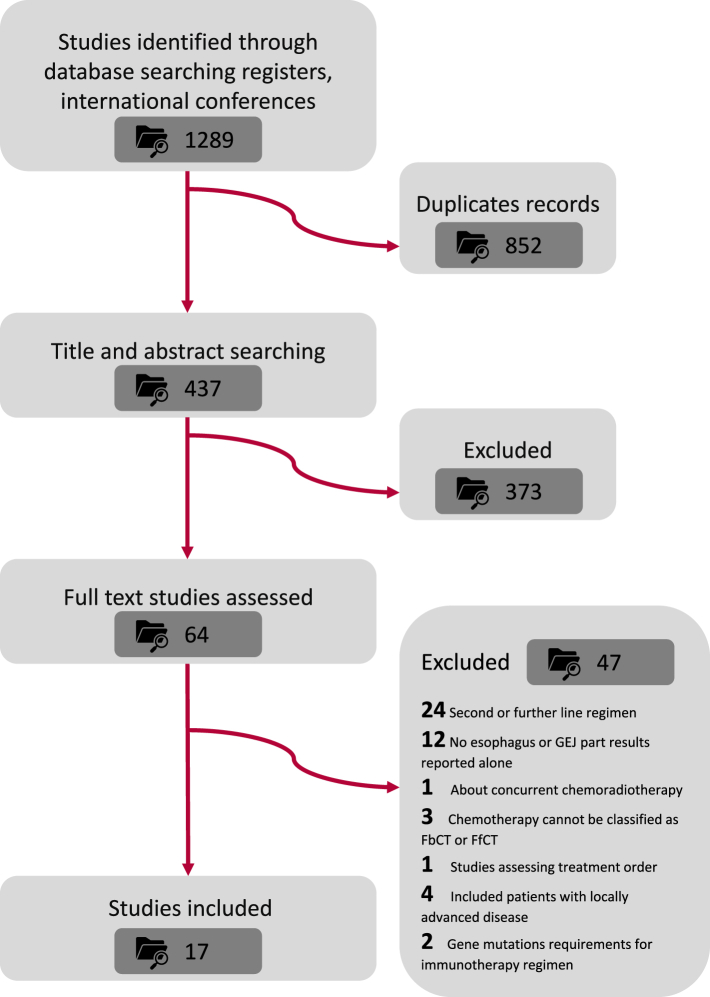
Study search and inclusion. The process of literature screening, inclusion, and exclusion followed the PRISMA guidelines. ICI, Immune checkpoint inhibitors; PRISMA, Preferred Reporting Items for Systematic Reviews and Meta-Analysis; RCT, Randomized controlled trial.

**Fig. 2:**
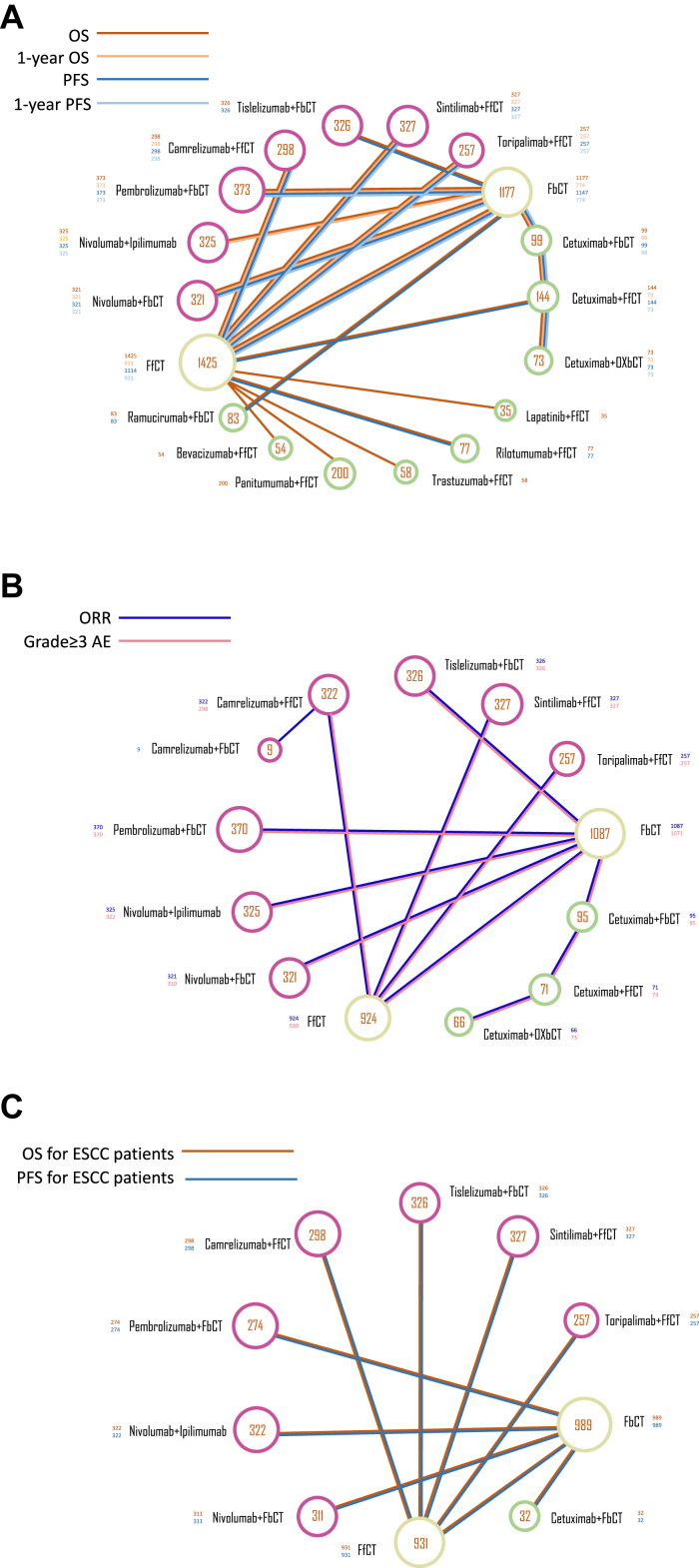
Network plot comparing treatment outcomes among different treatment groups of patients with esophageal cancer. (A) Comparisons on overall survival and progression-free survival with esophageal cancer. (B) Comparisons on adverse events ≥3 and objective response rate in patients with esophageal cancer. (C) Comparison of overall survival and progression-free survival with esophageal squamous cell carcinoma. Each round dot represents a type of treatment and the dot size represents the number of included patients. FbCT, 5-FU-based chemotherapy; FfCT, 5-FU-free chemotherapy; OXbCT, Oxaliplatin-based chemotherapy.

The process of screening and reviewing resulted in the retrieval of a total of 1289 records from the database search, and additional conference proceedings were also conducted. Sixty-four RCTs were deemed eligible for full-text review; eventually, 17 studies were included in the analysis (Fig. 1).^9^, ^10^, ^11^, ^12^^,^^18^, ^19^, ^20^, ^21^, ^22^, ^23^^,^^35^^,^^42^, ^43^, ^44^, ^45^, ^46^, ^47^ For statistical purposes, we divided chemotherapy regimens into two categories-fluorouracil-based chemotherapy (FbCT) and fluorouracil-free chemotherapy (FfCT).^48^ Finally, 9128 patients in the included studies had received the following 19 treatment regimens: nivolumab plus fluorouracil and cisplatin (nivo + FbCT), nivolumab plus ipilimumab (nivo + ipi), pembrolizumab plus fluorouracil and cisplatin (pembro + FbCT), placebo plus fluorouracil and cisplatin, camrelizumab plus paclitaxel and cisplatin (cam + FfCT), placebo plus paclitaxel and cisplatin, tislelizumab plus fluorouracil and cisplatin (tis + FbCT), sintilimab plus paclitaxel and cisplatin (sin + FfCT), toripalimab plus paclitaxel and cisplatin (tori + FfCT), fluorouracil plus cisplatin, cisplatin, cetuximab plus epirubicin, cisplatin and fluorouracil, cetuximab plus irinotecan and cisplatin (cetu + FfCT), cetuximab plus oxaliplatin, leucovorin and fluorouracil (cetu + FbCT), cetuximab plus fluorouracil and cisplatin (cetu + FbCT), fluorouracil and cisplatin, cetuximab plus capecitabine and cisplatin, capecitabine and cisplatin, lapatinib plus capecitabine and oxaliplatin, placebo plus capecitabine and oxaliplatin, rilotumumab plus epirubicin, cisplatin and capecitabine, placebo plus epirubicin, cisplatin and capecitabine, trastuzumab plus capecitabine and cisplatin (trastu + FfCT), placebo plus capecitabine and cisplatin, panitumumab plus epirubicin, oxaliplatin and capecitabine (pani + FfCT), epirubicin, oxaliplatin and capecitabine, bevacizumab plus capecitabine and cisplatin (beva + FfCT), ramucirumab plus fluorouracil and cisplatin (ramu + FbCT), and camrelizumab plus fluorouracil and cisplatin (cam + FbCT) (Fig. 2). Details in regimen information have been presented in Supplementary File S3.

Based on where these immunotherapy (IO) combinations were developed, we categorized them into domestic (toripalimab, tislelizumab, camrelizumab, and sintilimab) and foreign ICIs (pembrolizumab, nivolumab and ipilimumab).

### Bias assessment

During the literature bias assessment, five studies were assessed as low bias risk with RoB 2.0 tool due to their high-quality (Supplementary File S4).^49^ Finally, three studies were assessed as moderate risk since we have some concerns about the randomization process of Bleiberg1997, Lorenzen 2009 and ASCO e16084.

### Comparisons of OS, PFS, and ORR

**Fig. 3:**
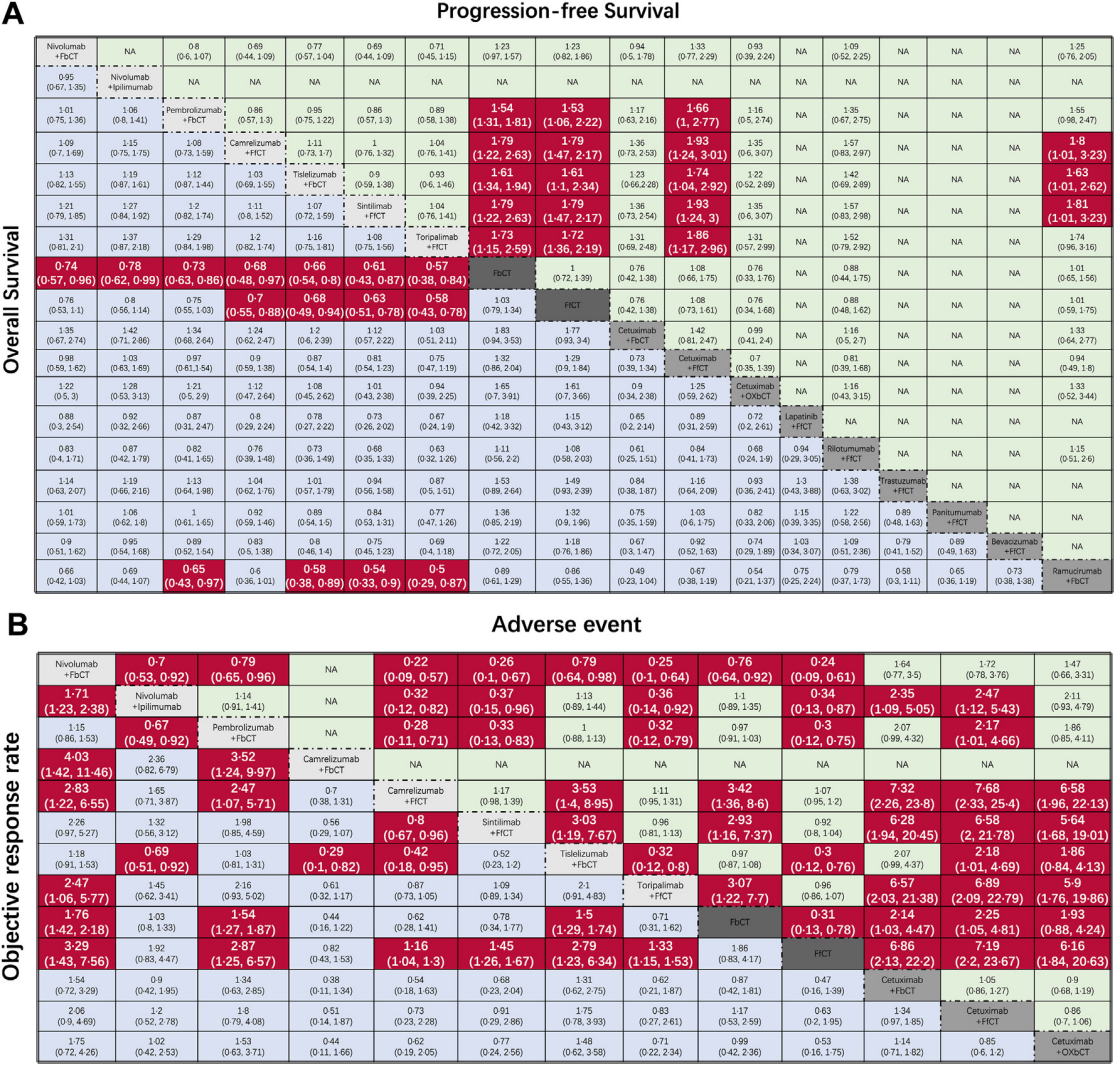
Survival and safety profiles of the Bayesian network meta-analysis in patients with metastatic EC. (A) Hazard Ratios (HR) and 95% confidence interval (CIs) for overall survival (lower triangle in purple) and progression-free survival (upper triangle in yellow). The statistics in each cell represents the hazard or odds ratios (95% confidential intervals) when comparing the column-defining regimen to the row-defining regimen. An HR value of less than 1 represents a favorable survival outcome. (B) Odd ratios and 95% CIs for objective response rate (lower triangle in purple) and grade ≥3 adverse events (upper triangle in yellow), and an OR value indicates better efficacy and safety. FbCT, 5-FU-based chemotherapy; FfCT, 5-FU-free chemotherapy; OXbCT, Oxaliplatin-based chemotherapy.

In terms of OS (Fig. 2, Fig. 3A), patients who underwent IO combination therapies were more likely to experience an improvement in their OS than those who received conventional chemotherapy. Among the investigated IO combinations, tori + FfCT seemed to obtain the best OS advantages (tori + FfCT vs. FbCT, HR 0.57, 95% CI 0.38–0.85; tori + FfCT vs. FfCT, HR 0.58, 95% CI 0.43–0.78). Sin + FfCT was comparable to tori + FfCT in providing OS benefit (sin + FfCT vs. FfCT, HR 0.63, 95% CI 0.51–0.78).

Regarding PFS (Fig. 3A), IO combinations provided better PFS than conventional chemotherapy. Among the investigated IO combinations, cam + FfCT demonstrated the best PFS advantages (FbCT vs. cam + FfCT, HR 1.79, 95% CI 1.22–2.63; FfCT vs. cam + FfCT, HR 1.79, 95% CI 1.47–2.17). Sin + FfCT was comparable to cam + FfCT in providing PFS benefit (sin + FfCT vs. cam + FfCT, HR 1, 95% CI 0.76–1.32). 1-year OS and PFS comparisons also revealed similar results (Supplementary File S5). Moreover, IO combinations yielded superior PFS benefits than chemotherapy-targeted combined therapy such as cetu + FfCT (cetu + FfCT vs. tori + FfCT, HR 1.86, 95% CI 1.17–2.96) and ramu + FbCT (ramu + FbCT vs. sin + FfCT, HR 1.81, 95% CI 1.01–3.23).

In terms of ORR, the efficacy outcomes exhibited dissimilarities compared to the PFS. Nivo + FbCT was observed to be the best treatment with regard to the objective response (nivo + FbCT vs. FfCT, OR 3.29, 95% CI 1.43–7.56), which was followed by pembro + FbCT (pembro + FbCT vs. FfCT, OR 2.87, 95% CI 1.25–6.57). Dual-/triple chemotherapy regimens were also revealed to be comparable to chemotherapy-targeted combined therapy in providing both OS and PFS benefits.

### Safety and toxicity

Safety and toxicity were identified in accordance with AEs of any grade and grade ≥3 (Fig. 2B). According to the conventional chemotherapy regimen, tori + FfCT (FbCT vs. tori + FfCT, OR 3.07, 95% CI 1.22–7.7), sintilimab + chemo (FbCT vs. sintilimab + chemo, OR 2.93, 95% CI 1.16–7.37), and cam + FfCT (FbCT vs. cam + FfCT, OR 3.42, 95% CI 1.36–8.6) had the lowest toxicities (Fig. 3B). However, the incorporation of immunotherapy resulted in increased toxicity profiles in nivo + FbCT (FfCT vs. nivo + FbCT, OR 0.24, 95% CI 0.09–0.61), nivo + ipi (FfCT vs. nivo + ipi, OR 0.34, 95% CI 0.13–0.87), and pembro + FbCT (FfCT vs. pembro + FbCT, OR 0.3, 95% CI 0.12–0.75). Frequently reported treatment-related AEs for the IO combinations included nausea, leukopenia, neutropenia, asthenia and anemia. Immune-related AEs included hypothyroidism, rash and pruritus (Supplementary File S6). The frequencies of these specific AEs varied across IO combinations. Pembro + FbCT exhibited the highest probability of causing nausea and decreased appetite, whereas, cam + FfCT and tori + FfCT were associated with the highest risk of leukopenia, neutropenia, and anemia.

### Subgroup analysis

**Fig. 4:**
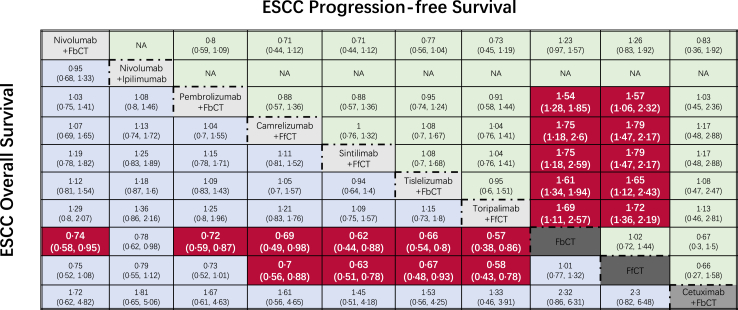
Survival profiles of the Bayesian network meta-analysis in patients with esophageal squamous cell carcinoma. FbCT, 5-FU-based chemotherapy; FfCT, 5-FU-free chemotherapy; OXbCT, Oxaliplatin-based chemotherapy.

In ESCC subgroup analysis, 10 different regimens (7 immunochemotherapy, 1 targeted therapy and 2 chemotherapy regimens) were included (Fig. 4A). Similar to the overall analysis results, tori + FfCT obtained the lowest HR (tori + FfCT vs. FbCT, HR 0.57, 95% CI 0.38–0.86; tori + FbCT vs. FfCT, HR 0.58, 95% CI 0.43–0.78) compared to chemotherapy. Furthermore, sin + FfCT and tisle + FbCT indicate excellent performance in OS benefit. On the contrary, sin + FfCT was found to be the most appropriate regimen for ESCC patients to improve PFS (FfCT vs. sin + FfCT, HR 1.75, 95% CI 1.18–2.59; FfCT vs. sin + FfCT, HR 1.79, 95% CI 1.47–2.17).

Nivolumab + FbCT shows a huge advantage in ORR compared to chemotherapy (nivo + FbCT vs. FbCT, OR 1.76, 95% CI 1.42–2.18; nivo + FbCT vs. FfCT, OR 3.29, 95% CI 1.42–7.57) and three immunochemotherapy regimes (nivo + FbCT vs. cam + FbCT, OR 4.04, 95% CI 1.42–11.48; nivo + FbCT vs. cam + FfCT, OR 2.83, 95% CI 1.21–6.57; nivo + FbCT vs. tori + FfCT, OR 2.47, 95% CI 1.06–5.77) (Fig. 4B). In terms of safety, the majority of domestic and foreign IO combinations have demonstrated distinctive safety profiles. Nivo + FbCT, nivo + ipi, and pembro + FbCT had significantly more AEs than chemotherapy alone (FfCT vs. nivo + FbCT, OR 0.24, 95% CI 0.09–0.61; FfCT vs. nivo + ipi, OR 0.34, 95% CI 0.13–0.87; FfCT vs. pembro + FbCT, OR 0.3, 95% CI 0.12–0.75). Conversely, all domestic IO combinations except tisle + FbCT (FfCT vs. tisle + FbCT, OR 0.3, 95% CI 0.13–0.78), such as cam + FfCT, sin + FfCT, and tori + FfCT, demonstrated safer profiles than chemotherapy.

### Rankings

**Fig. 5:**
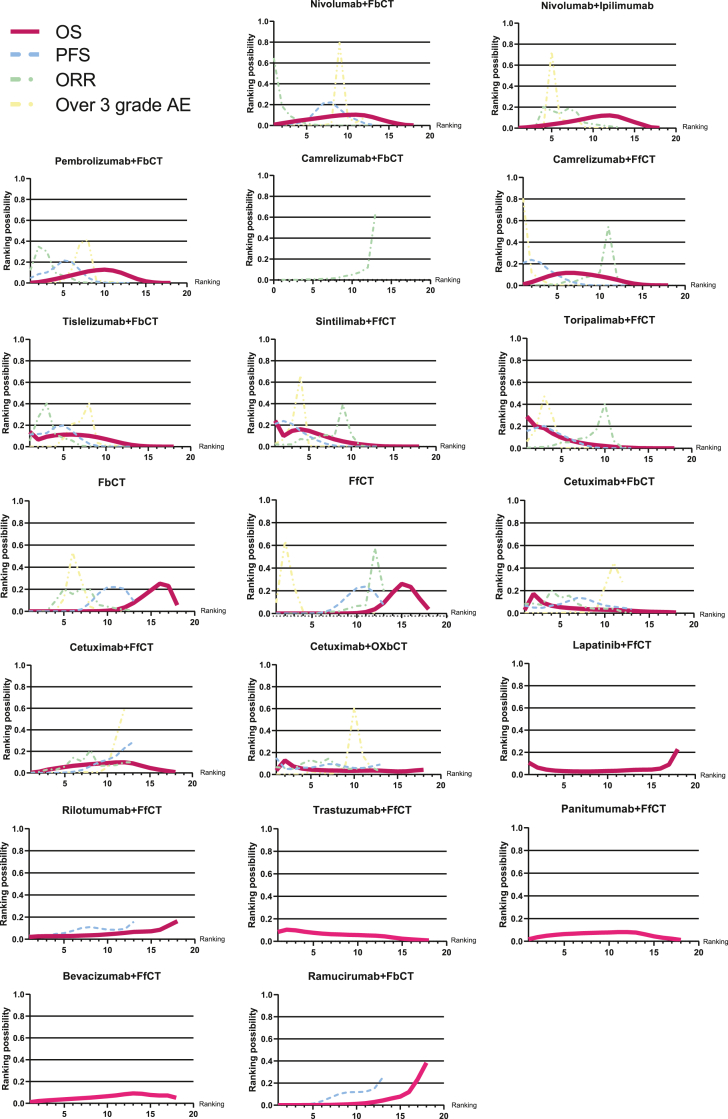
Bayesian ranking profiles for immunotherapy combinations on efficacy and safety for patients with esophageal cancer. Ranking plots depict the probability of each immunotherapy combination being ranked from first to last regarding OS, PFS, ORR, and AEs. OS, Overall survival; PFS, Progression-free survival; AE, adverse events; FbCT, 5-FU-based chemotherapy; FfCT, 5-FU-free chemotherapy; OXbCT, Oxaliplatin-based chemotherapy.

According to the Bayesian ranking profiles (SUCRA value), the ranking possibilities of all 19 treatment regimens mentioned in the included studies are shown (Fig. 5). The ranking outcomes align with the direct/indirect results obtained by HRs and ORs. It is worth mentioning that the ranking results of AEs were reverse ranked for a better understanding of the ranking sequence, which means the regimen would lead to less grade ≥3 AEs incidence with a higher rank. Similar to HR results in league tables, tori + FfCT obtained the first rank (SUCRA 0.290) for OS while sin + FfCT ranked second (SUCRA 0.241). Tisle + FbCT ranked third for OS of advanced and metastatic esophageal cancer with a SUCRA value of 0.139. Regarding PFS, the regimens of FfCT combined with sintilimab and camrelizumab ranked first and second with similar SUCRA of 0.211 and 0.210, respectively. The detailed SUCRA value data are displayed in Supplementary File S7.

### Heterogeneity, inconsistency, and transitivity assessment

The heterogeneity of study results was assessed using the I^2^ test. According to I^2^ test results, all the comparisons (OS, PFS, 1 year OS, 1 year PFS, ORR, DCR, ≥ grade 3 AEs, and OS, PFS, ORR, ≥ grade 3 AEs for ESCC) had a low heterogeneity since all the I^2^ value were ≤25% as defined before (Supplementary File S8). Furthermore, the head-to-head forest plots (Supplementary File S9) showed low heterogeneity in our analysis. An inconsistency test was conducted for demonstrating the consistency between direct and indirect comparisons. Analysis of the inconsistency showed a low inconsistency in our study; all the P values in the inconsistency test were >0.05 (Supplementary File S10). Transitivity assessment also shows great consistency and transitivity in included studies (Supplementary File S11). No relevant deviation in OS (Supplementary File S12A) and PFS (Supplementary File S12B) was shown in the node-splitting analysis of inconsistency. The forest plots for the main network meta-analysis results are present in Supplementary File S13. The convergence of iterations was evaluated as good in trace and density plots (Supplementary File S14).

## Discussion

To the best of our knowledge, this is the first NMA to comprehensively compare the efficacy and safety profiles of IO combinations for advanced and metastatic esophageal cancer in use. The main findings of this study provide evidence for clinical application, including the following:

1. IO combinations provided superior OS and PFS than conventional chemotherapy and chemotherapy-targeted combined therapy. Among the investigated IO combinations, tori + FfCT obtained the best OS advantages; whereas, cam + FfCT and sin + FfCT combinations brought comparable best PFS benefits.
2. The addition of tori + FfCT, sin + FfCT, and cam + FbCT did not increase toxicity when compared to conventional chemotherapy.
3. Nivo + FbCT and pembro + FbCT obtained OR rates better than those obtained from other IO combinations.
4. In the ESCC group, pembro + FbCT, cam + FfCT, sin + FfCT, tisle + FbCT, and tori + FfCT showed superior OS and PFS survival benefits to the conventional chemotherapy group.
5. Efficacy and safety showed satisfactory and balanced characteristics in pembro + FbCT, cam + FfCT, sin + FfCT, tisle + FbCT, and tori + FfCT combinations when investigating the whole population and ESCC subgroups.

There are several explanations for these findings. First, the synergistic effect of immunotherapy and chemotherapy has been confirmed in clinical and preclinical medicine.^50^, ^51^, ^52^ Some chemotherapeutic agents can promote immunogenic cell death (ICD), a mechanism by which cancer cells undergoing apoptosis produce molecules known as damage-associated molecular patterns (DAMPs). The immune system could be stimulated by these molecules and induce cancer cells’ recognition and elimination. This immune activation can enhance the effectiveness of immunotherapy, particularly immune checkpoint inhibitors.^53^ In addition, the application of chemotherapy has been found to deplete both tumour-infiltrating and circulating Treg cells, inducing the activation of protective anticancer immunity. The tumor microenvironment (TME) comprises a number of various cell types, extracellular matrix components, and signaling molecules that surround and interact with cancer cells. CD8+ cells infiltration and phenotype of tumor infiltrating lymphocytes (TILs) are potential biomarkers showing up in TME for ICIs efficacy. Chemotherapy agents were found to elevate the number of CD8+ TILs through the TME.^54^ Chemotherapy can also modulate the TME in ways that could either promote or inhibit tumor incidence and development and have a significant impact on the TME modulation that help create an environment conducive to the immune system to attack cancer cells through that improving the efficacy of immunotherapy.^55^ Chemotherapy can alter the tumor microenvironment by decreasing the presence of immunosuppressive cells, including regulatory T cells (Tregs), myeloid-derived suppressor cells (MDSCs), and tumor-associated macrophages (TAMs).^56^ However, according to results from a pooled research including 13 phase III clinical trials, there is no evidence about the interaction enhancement effect between ICIs and other therapies including chemotherapy.^57^ The authors believed that the benefit from a combination of ICIs and chemotherapy can be explained as increasing the chance of a single-agent response to individual patient. While some combinations may not demonstrate a clear synergistic effect, ongoing research aims to optimize the use of combination therapies and identify biomarkers that can predict treatment response. Cytotoxic chemotherapy demonstrated beneficial immunomodulatory effects via releasing of numerous tumor antigens.^52^ Moreover, chemotherapy induces tumor stroma disruption and provides cytotoxic lymphocytes more access to the tumor site.^58^ Besides enhancing the anti-tumor effects of immunotherapy, chemotherapeutics also help to reverse immunosuppression by eliminating myeloid-derived suppressor cells (MDSC) and T regulatory cells (Treg) and decreasing the production of immune suppressive cytokines.^51^ Second, the different performance between domestic and foreign IO combinations could be attributed to their chemical structures and additional activation of immuno-cytotoxic pathway.^59^^,^^60^ For instance, toripalimab is a fully humanized IgG4 that exhibits a great binding affinity to PD-1. In comparison to nivolumab and pembrolizumab, its dominant binding to FG loop of PD1 with a lengthy complementarity-determining regions 3 (CDR3) loop of a heavy chain is significantly different.^61^ Subsequent induction of PD-1 receptor endocytosis enables the reduction of PD-1 expression on the cell membrane surface.^62^ Therefore, these factors could be the key reasons for better OS/PFS survival benefit of domestic IO combinations such as tori + FfCT than pembro + FbCT and nivo + FbCT/ipi. Although these findings showed better OS/PFS data in domestic IO combinations, there lack a head-to-head comparison between domestic and foreign IO combinations. Therefore, it is still early to conclude a hasty conclusion on the optimal IO combination.

Surprisingly, compared to conventional chemotherapy, adding several domestic IO such as toripalimab, sintilimab, and camrelizumab did not increase the grade of AEs ≥3. However, for foreign IO combinations such as pembro + FbCT and nivo + FbCT/+ipi, increased AEs were observed. These findings suggested that IO combinations had relatively manageable safety profiles and domestic IO combinations seemed to be safer options. However, AEs associated with immunotherapy, such as hypothyroidism, rash, and pneumonitis, can vary in severity from milad to fatal. It is noteworthy that camrelizumab has a distinct side effect of reactive capillary endothelial proliferation, which occurs in approximately 80% of cases. Hence, irAEs should also be closely monitored and actively managed clinically.

It is worth noting that nivo and pembro-based IO combinations obtained better ORR than domestic IO combinations did. The discordance between radiological response and survival benefit may be due to two reasons. First, ORR is considered a positive early indicator of benefit.^63^ The high ORR yet low survival benefit may indicate that foreign IO combinations induce a better tumor response during the early phase but have a relatively weaker ability to prolong long-term survival. Second, the majority of trials have implemented the updated response evaluation criteria in solid tumors (revised RECIST ver 1.1) to assess the efficacy of treatment.^64^ However, the exact dimension of an esophageal tumor cannot be determined due to the luminal anatomy of the esophagus. Additionally, the requirement that the short-axis lengths of metastatic lymph nodes be greater than 1.5 cm may reduce the likelihood of missing smaller metastatic lymph node foci. Implementing morphology-based criteria to assess the effectiveness of IO combinations is therefore challenging.

The present investigation exhibits certain constraints. Indirect comparison between IO combination and other chemo-regimens should be performed via studies comparing FfCT and FbCT regimens. However, the participation size was small in the included chemo-only study.^9^ More data for efficacy and safety comparisons between FfCT and FbCT are needed. On the other hand, of the immunotherapeutics in use, this NMA only investigated PD-1 and CTLA-4 inhibitors-based combinations, which may hinder the discovery of better IO combinations. Third, the present investigation employed a fixed effects model for the network meta-analysis. Given the anticipated methodological heterogeneity among the included studies, a fixed-effects model is more suitable for addressing this condition. Another reason is that when networks exhibit sparsity, it is possible for random-effect models to produce credible intervals that are excessively wide, thereby leading to implausible estimates in network meta-analysis. It can be challenging to make reliable inferences about the treatment properties, even in cases where the direct and indirect estimates exhibit coherent. In instances of such occurrences, a network meta-analysis with fixed effects was conducted, or alternatively, the direct estimates were employed as the most suitable estimates of treatment characteristics.^38^^,^^39^ Finally, we want to express our concern about the bias evaluation of included studies performed by RoB 2.0. Except for 8 targeted therapy relative studies including gastric cancer patients, for which we manually extracted esophageal or gastroesophageal junction data, we included two unpublished clinical trials and two studies for which we obtained data from Kaplan–Meier curves. However, no closely related evaluation criteria of data bias are available in RoB 2.0. A more elaborate literature evaluation tool is needed for further meta-analysis, particularly for studies that require data screening.

By gathering data synthesized from high-quality RCTs, this meta-analysis provides medical practitioners with a source of data to evaluate the efficacy and safety of various promising alternatives within clinical practice. Our study suggested that IO combinations provided better OS and PFS than conventional chemotherapy and chemotherapy-targeted combined therapy. Furthermore, compared to foreign IO combinations, sin + FfCT, tori + FfCT, cam + FfCT, and tisle + FbCT were superior first-line treatment options for patients with advanced and metastatic esophageal cancer. Although the foreign IO combinations, such as pembro + FbCT and nivo + FbCT, obtained better ORR than other IO combinations, the addition of chemotherapy to IO worsens its safety profile. Our findings could provide complementary evidence for current guideline recommendations under the premise of cautious interpretation.

## Contributors

Shujie Huang, Sichao Wang & Zhen Gao: Conceptualization, Data curation, Formal analysis, Validation, Roles/Writing–original draft, Writing–review & editing; Dezhao Tang, Wei Xu & Ruijie Zeng: Methodology, Resources, Writing–review & editing; Guibin Qiao: Conceptualization, Project administration, Resources, Supervision, Writing–review & editing.

## Data sharing statement

All data needed to evaluate the conclusions in the paper are present in the paper and/or the Supplementary Materials.

## Declaration of interests

All authors have completed the ICMJE uniform disclosure form and declare: no support from any organization for the submitted work; no financial relationships with any organizations that might have an interest in the submitted work in the previous three years; no other relationships or activities that could appear to have influenced the submitted work.
