# Supplementary material for: Efficacy and safety of immunochemotherapy, immunotherapy, chemotherapy, and targeted therapy as first-line treatment for advanced and metastatic esophageal cancer: a systematic review and network meta-analysis
Source: Lancet Reg Health West Pac. 2023 Jul 6;38:100841. doi: 10.1016/j.lanwpc.2023.100841 (PMC10339186; doi:10.1016/j.lanwpc.2023.100841)
Supplement: Supplementary Files S1–S14 [file mmc1.pdf]

Efficacy and Safety of Immunochemotherapy, Immunotherapy, Chemotherapy, and Targeted Therapy as First-line Treatment for Advanced Esophageal Cancer: A Systematic Review and Network Meta-analysis

Supplementary Appendix

Material catalogue

Content

Supplementary File 1. Checklist of the PRISMA extension for Systematic Review and Network Meta-Analysis 3
Supplementary File 2. Search terms for meta-analysis. 5
Supplementary File 3. Baseline characteristics of studies included in the network meta-analysis. 6
Supplementary File 4. Cochrane risk of the bias assessment tool. 10
Supplementary File 5. 1-year survival profiles of the Bayesian network meta-analysis in patients with esophageal cancer. 11
Supplementary File 6. Overall adverse events of any grade among different treatment groups. 12
Supplementary File 7. Bayesian ranking results (SUCRA value) of network meta-analysis. 13
Supplementary File 8. Heterogeneity test 16
Supplementary File 9. Forest plots for (A) pooled (pair-wise, and network) and (B) indirect (back-calculated) analysis. 17
Supplementary File 10. Inconsistency test. 18
Supplementary File 11. Transitivity assessment. 19
Supplementary File 12. Node-splitting analysis of inconsistency. 20
Supplementary File 13. The forest plots for main network meta-analysis results. 21
Supplementary File 14. Trace and density plot. 23

Supplementary File 1. Checklist of the PRISMA extension for Systematic Review and Network Meta-Analysis

Section and Topic	Item #	Checklist item	Location where item is reported
**TITLE**			
Title	1	Identify the report as a systematic review *incorporating a network meta-analysis (or related form of meta-analysis).*	Page 1
**ABSTRACT**			
Abstract<br><br>Structured summary	2	Provide a structured summary including, as applicable:<br>◎**Background:** main objectives.<br>◎**Methods:** data sources; study eligibility criteria, participants, and interventions; study appraisal; and *synthesis methods, such as network meta-analysis.*<br>◎**Results:** number of studies and participants identified; summary estimates with corresponding confidence/credible intervals; *treatment rankings may also be discussed. Authors may choose to summarize pairwise comparisons against a chosen treatment included in their analyses for brevity.*<br>◎**Discussion/Conclusions:** limitations; conclusions and implications of findings.<br>◎**Other:** primary source of funding; systematic review registration number with registry name.	Page 3-5
**INTRODUCTION**			
Rationale	3	Describe the rationale for the review in the context of what is already known*, including mention of why a network meta-analysis has been conducted.*	Page 8-10
Objectives	4	Provide an explicit statement of questions being addressed, with reference to participants, interventions, comparisons, outcomes, and study design (PICOS).	Page 10
**METHODS**			
Protocol and registration	5	Indicate whether a review protocol exists and if and where it can be accessed (e.g., Web address); and, if available, provide registration information, including registration number.	Page 10-11
Eligibility criteria	6	Specify study characteristics (e.g., PICOS, length of follow-up) and report characteristics (e.g., years considered, language, publication status) used as criteria for eligibility, giving rationale. *Clearly describe eligible treatments included in the treatment network, and note whether any have been clustered or merged into the same node (with justification).*	Page 11-12
Information sources	7	Describe all information sources (e.g., databases with dates of coverage, contact with study authors to identify additional studies) in the search and date last searched.	Page 11
Search	8	Present full electronic search strategy for at least one database, including any limits used, such that it could be repeated.	Page 11
Study selection	9	State the process for selecting studies (i.e., screening, eligibility, included in systematic review, and, if applicable, included in the meta-analysis).	Page 11
Data collection process	10	Describe method of data extraction from reports (e.g., piloted forms, independently, in duplicate) and any processes for obtaining and confirming data from investigators.	Page 12
Data items	11	List and define all variables for which data were sought (e.g., PICOS, funding sources) and any assumptions and simplifications made.	Page 12
**Geometry of the network**	**S1**	Describe methods used to explore the geometry of the treatment network under study and potential biases related to it. This should include how the evidence base has been graphically summarized for presentation, and what characteristics were compiled and used to describe the evidence base to readers.	Page 12-13
Risk of bias in individual studies	12	Describe methods used for assessing risk of bias of individual studies (including specification of whether this was done at the study or outcome level), and how this information is to be used in any data synthesis.	Page 12
Summary measures	13	State the principal summary measures (e.g., risk ratio, difference in means). *Also describe the use of additional summary measures assessed, such as treatment rankings and surface under the cumulative ranking curve (SUCRA) values, as well as modified approaches used to present summary findings from meta-analyses.*	Page 12-13
Synthesis of results	14	Describe the methods of handling data and combining results of studies for each network meta-analysis. This should include, but not be limited to:<br>◎*Handling of multi-arm trials;*<br>◎*Selection of variance structure;*<br>◎*Selection of prior distributions in Bayesian analyses; and*<br>◎*Assessment of model fit.*	Page 12-14
**Assessment of Inconsistency**	**S2**	Describe the statistical methods used to evaluate the agreement of direct and indirect evidence in the treatment network(s) studied. Describe efforts taken to address its presence when found.	Page 12-13
Risk of bias across studies	15	Specify any assessment of risk of bias that may affect the cumulative evidence (e.g., publication bias, selective reporting within studies).	Page 12-13
Additional analyses	16	Describe methods of additional analyses, if done, indicating which were pre-specified. This may include, but not be limited to the following:<br>◎Sensitivity or subgroup analyses;<br>◎Meta-regression analyses;<br>◎*Alternative formulations of the treatment network; and*<br>◎*Use of alternative prior distributions for Bayesian analyses (if applicable).*	Page 12-13
**RESULTS**			
Study selection	17	Give numbers of studies screened, assessed for eligibility, and included in the review, with reasons for exclusions at each stage, ideally with a flow diagram.	Page 14
**Presentation of**	**S3**	Provide a network graph of the included studies to enable visualization of the geometry of the treatment network	Page 14

Section and Topic	Item #	Checklist item	Location where item is reported
**network structure**			
**Summary of network geometry**	**S4**	Provide a brief overview of characteristics of the treatment network. This may include commentary on the abundance of trials and randomized patients for the different interventions and pairwise comparisons in the network, gaps of evidence in the treatment network, and potential biases reflected by the network structure.	Page 14-15
Study characteristics	18	For each study, present characteristics for which data were extracted (e.g., study size, PICOS, follow-up period) and provide the citations.	Page 14-15
Risk of bias within studies	19	Present data on risk of bias of each study and, if available, any outcome level assessment (see item 12).	Page 15
Results of individual studies	20	For all outcomes considered (benefits or harms), present, for each study:1) simple summary data for each intervention group, and 2) effect estimates and confidence/credible intervals. *Modified approaches may be needed to deal with information from larger networks.*	Page 15-18
Synthesis of results	21	Present results of each meta-analysis done, including confidence/credible intervals. *In larger networks, authors may focus on comparisons versus a particular comparator (e.g., placebo or standard care), with full findings presented in an appendix. League tables and forest plots may be considered to summarize pairwise comparisons.* If additional summary measures were explored (such as treatment rankings), these should also be presented.	Page 15-18
**Exploration for inconsistency**	**S5**	Describe results from investigations of inconsistency. This may include such information as measures of model fit to compare consistency and inconsistency models, *P* values from statistical tests, or summary of inconsistency estimates from different parts of the treatment network.	Page 18-19
Risk of bias across studies	22	Present results of any assessment of risk of bias across studies (see Item 15).	Page 18-19
Additional analysis	23	Give results of additional analyses, if done (e.g., sensitivity or subgroup analyses, meta-regression, *alternative network geometries studied, alternative choice of prior distributions for Bayesian analyses,* and so forth [see Item 16]).	Page 17-18
**DISCUSSION**			
Summary of evidence	24	Summarize the main findings including the strength of evidence for each main outcome; consider their relevance to key groups (e.g., healthcare providers, users, and policy makers).	Page 19
Limitations	25	Discuss limitations at study and outcome level (e.g., risk of bias), and at review-level (e.g., incomplete retrieval of identified research, reporting bias). *Comment on the validity of the assumptions, such as transitivity and consistency. Comment on any concerns regarding network geometry (e.g., avoidance of certain comparisons).*	Page 23-24
Conclusions	26	Provide a general interpretation of the results in the context of other evidence, and implications for future research.	Page 24
**FUNDING**			
Funding	27	Describe sources of funding for the systematic review and other support (e.g., supply of data); role of funders for the systematic review. This should also include information regarding whether funding has been received from manufacturers of treatments in the network and/or whether some of the authors are content experts with professional conflicts of interest that could affect use of treatments in the network.	Page 5

PRISMA = Preferred Reporting Items for Systematic Reviews and Meta-Analysis; PICOS = population, intervention, comparators, outcomes, study design.

*The Checklist of the PRISMA extension is formulated for RICE-META1st with the structure of *PRISMA NMA Checklist of Items to Include When Reporting a Systematic Review Involving a Network Meta-analysis* in https://prisma-statement.org/Extensions/NetworkMetaAnalysis

Supplementary File 2. Search terms for meta-analysis.

(((((((((((((((((((esophagus cancer[title] OR oesophagus cancer[title]) OR esophagus neoplasms[title]) OR oesophagus neoplasms[title]) OR esophagogastric junction cancer[title]) OR esophagogastric junction neoplasms[title]) OR gastroesophageal junction cancer[title]) OR gastroesophageal junction neoplasms[title]) OR gastro-oesophageal junction cancer[title]) OR esophagus adenocarcinoma[title]) OR oesophagus adenocarcinoma[title]) OR esophagus squamous cell carcinoma[title]) OR oesophagus squamous cell carcinoma[title]) OR esophagogastric junction adenocarcinoma[title]) OR gastroesophageal junction adenocarcinoma[title]) OR gastro-oesophageal junction adenocarcinoma[title]) OR esophagogastric junction squamous cell carcinoma [title]) OR gastroesophageal junction squamous cell carcinoma [title]) OR gastro-oesophageal junction squamous cell carcinoma [title]) AND ((Immune Checkpoint Inhibitors [title/abstract] OR Immune Checkpoint Inhibitor [title/abstract]) OR Immune Checkpoint Blockers [title/abstract]) OR Immune Checkpoint Blockade [title/abstract]) OR Immune Checkpoint Inhibition [title/abstract]) OR PD-L1 Inhibitors [title/abstract]) OR PD L1 Inhibitors [title/abstract]) OR PD-L1 Inhibitor [title/abstract]) OR PD L1 Inhibitor[title/abstract]) OR Programmed Death-Ligand 1 Inhibitors [title/abstract]) OR Programmed Death Ligand 1 Inhibitors [title/abstract]) OR CTLA-4 Inhibitors [title/abstract]) OR CTLA 4 Inhibitors[title/abstract]) OR CTLA-4 Inhibitor[title/abstract]) OR CTLA 4 Inhibitor [title/abstract]) OR Cytotoxic T-Lymphocyte-Associated Protein 4 Inhibitors [title/abstract]) OR Cytotoxic T Lymphocyte Associated Protein 4 Inhibitors [title/abstract]) OR Cytotoxic T-Lymphocyte-Associated Protein 4 Inhibitor [title/abstract]) OR Cytotoxic T Lymphocyte Associated Protein 4 Inhibitor [title/abstract]) OR PD-1 Inhibitors [title/abstract]) OR PD 1 Inhibitors [title/abstract]) OR PD-1 Inhibitor [title/abstract]) OR PD 1 Inhibitor [title/abstract]) OR Programmed Cell Death Protein 1 Inhibitor [title/abstract]) OR Programmed Cell Death Protein 1 Inhibitors [title/abstract]) OR PD-1-PD-L1 Blockade [title/abstract]) OR PD 1 PD L1 Blockade [title/abstract]) OR Pembrolizumab [title/abstract]) OR SCH-900475 [title/abstract]) OR Keytruda [title/abstract]) OR MK-3475 [title/abstract]) OR lambrolizumab [title/abstract]) OR Nivolumab [title/abstract]) OR Opdivo [title/abstract] OR ONO-4538 [title/abstract]) OR ONO 4538 [title/abstract]) OR ONO4538 [title/abstract]) OR MDX-1106 [title/abstract]) OR MDX 1106 [title/abstract]) OR MDX1106 [title/abstract]) OR BMS-936558 [title/abstract]) OR BMS 936558 [title/abstract]) OR BMS936558 [title/abstract]) OR Cemiplimab [title/abstract]) OR Spartalizumab[title/abstract]) OR PDR-001[title/abstract]) OR PDR001 [title/abstract]) OR anti-Pd1 monoclonal antibody PDR001[title/abstract]) OR Camrelizumab [title/abstract]) OR SHR-1210 [title/abstract]) OR SHR 1210 [title/abstract]) OR Sintilimab [title/abstract]) OR IBI 308 [title/abstract]) OR IBI308 [title/abstract]) OR IBI-308 [title/abstract]) OR Tislelizumab [title/abstract]) OR Toripalimab[title/abstract]) OR Atezolizumab [title/abstract]) OR MPDL3280A [title/abstract]) OR MPDL-3280A[title/abstract]) OR Tecentriq [title/abstract]) OR RG7446 [title/abstract]) OR RG-7446 [title/abstract]) OR Avelumab [title/abstract]) OR MSB-0010682 [title/abstract]) OR MSB0010682 [title/abstract]) OR bavencio [title/abstract]) OR MSB0010718C [title/abstract]) OR MSB-0010718C [title/abstract]) OR Durvalumab [title/abstract]) OR MEDI4736 [title/abstract]) OR MEDI-4736 [title/abstract]) OR Imfinzi [title/abstract]) OR first-line[title/abstract]) OR first line[title/abstract]) OR treatment-naive[title/abstract]) OR treatment- naïve[title/abstract]) OR untreated[title/abstract]) OR treatment[title/abstract] OR therapy[title/abstract])) AND ((((((Randomized Controlled Trial[ptyp] OR controlled clinical trial[ptyp]) OR randomized[title/abstract]) OR randomised[title/abstract]) OR randomly[title/abstract]) OR trial[title/abstract]) OR phase[title/abstract])) AND (English[Language])) AND ("0001/01/01"[Date - Publication] : "2022/05/01"[Date - Publication])

Supplementary File 3. Baseline characteristics of studies included in the network meta-analysis.

Study	All	Patients number in disease histology		Patients number in disease site		Intervention	Patients number in reported outcomes								
		ESCC	EAC	EC	GEJ		OS			PFS			ORR	AEs	
							All	1-year in all	ESCC	All	1-year in all	ESCC		Any grade	Grade ≥ 3
CheckMate 648	970	951	NA	970	0	Nivolumab (240 mg every 2 weeks) plus fluorouracil (800 mg/m^2) and cisplatin (80mg/m^2), 4-week cycle	321	321	311	321	321	311	321	310	310
						Nivolumab (3 mg/kg every 2 weeks) plus ipilimumab (1 mg/kg every 6 weeks)	325	325	322	325[¶]	325[¶]	322	325	322	322
						Fluorouracil (800 mg/m^2 4-week cycle) and cisplatin (80mg/m^2), 4-week cycle	324	324	318	324	324	318	324	304	304
KEYNOTE-590	749	548	201	658	91	Pembrolizumab (200mg) plus fluorouracil (80 mg/m^2 on day 1-5) and cisplatin (80 mg/m^2 on day 1) once every 3 weeks for up to 35 cycles	373	373	274	373	373	274	373	370	370
						Placebo plus fluorouracil (80 mg/m^2 on day 1-5) and cisplatin (80 mg/m^2 on day 1) once every 3 weeks for up to 35 cycles	376	376	274	376	376	274	376	370	370
ESCORT-1st	596	596	0	596	0	Camrelizumab (200 mg on day 1) plus paclitaxel (175 mg/m^2 on day 1) and cisplatin (75 mg/m^2 on day 1), 3-week cycles	298	298	298	298	298	298	298	298	298
						Placebo plus paclitaxel (175 mg/m2 on day 1) and cisplatin (75 mg/m2 on day 1), 3-week cycles	298	298	298	298	298	298	298	297	297

RATIONALE-306[ϕ]	649	649	0	649	0	Tislelizumab (200 mg) plus fluorouracil and cisplatin, 3-week cycles	326	NA	326	326	NA	326	326	326	326
						Placebo plus fluorouracil and cisplatin, 3-week cycles	323	NA	323	323	NA	323	323	323	323
ORIENT-15	659	659	0	659	0	Sintilimab (3 mg/kg in patients weighing <60 kg or 200 mg in patients weighing ≥60 kg on day 1) plus paclitaxel (75 mg/m^2 on day 1 of each cycle) and cisplatin (87.5 mg/m^2 on day 1 and day 8 of cycle 1; 175 mg/m^2 on day 1 of the other cycles)	327	327	327	327	327	327	327	327	327
						Placebo plus paclitaxel (75 mg/m^2 on day 1 of each cycle) and cisplatin (87.5 mg/m^2 on day 1 and day 8 of cycle 1; 175 mg/m^2 on day 1 of the other cycles)	332	332	332	332	332	332	332	332	332
JUPITER-06	514	514	0	514	0	Toripalimab (240 mg on day 1) plus paclitaxel (175 mg/m^2) and cisplatin (75 mg/m^2), 3-week cycle	257	257	257	257	257	257	257	257	257
						Placebo plus paclitaxel (175 mg/m^2) and cisplatin (75 mg/m^2), 3-week cycle	257	257	257	257	257	257	257	257	257
Bleiberg1997	88	88	0	88	0	Fluorouracil (1g/m^2) plus cisplatin (100mg/m^2), 3-week cycle	44[§]	44[§]	44[§]	44[§]	44[§]	44[§]	34	44	44
						Cisplatin (100mg/m^2), 3-week cycle	44[§]	44[§]	44[§]	44[§]	44[§]	44[§]	37	44	44
CALGB 80403	245	23	222	91	119	Cetuximab (400 mg/m^2) plus epirubicin (50 mg/m^2), cisplatin (60 mg/m^2) and fluorouracil (200 mg/m^2), 3-week cycle	67[§]	67[§]	NA	67[§]	67[§]	NA	63	NA	67
						Cetuximab (400 mg/m^2) plus irinotecan (65 mg/m^2) and cisplatin (30 mg/m^2), 3-week cycle	73[§]	73[§]	NA	73[§]	73[§]	NA	71	NA	73
						Cetuximab (400 mg/m^2) plus oxaliplatin (85 mg/m^2), leucovorin (400 mg/m^2) and fluorouracil (400 mg/m^2)	73[§]	73[§]	NA	73[§]	73[§]	NA	66	NA	73

Lorenzen2009	62	62	0	62	0	Cetuximab (400mg/m^2) plus fluorouracil (1000 mg/m^2) and cisplatin (100 mg/m^2)	32	32	32	32	32	32	32	32	32
						Fluorouracil (1000 mg/m^2) and cisplatin (100 mg/m^2)	30	30	30	30	30	30	30	30	30
EXPAND	904	0	904	0	144	Cetuximab (400 mg/m^2) plus capecitabine (1000 mg/m^2 twice daily) and cisplatin (80 mg/m^2 on day 1), 3-week cycle	71	NA	NA	71	NA	NA	NA	NA	NA
						Capecitabine (1000 mg/m^2 twice daily) and cisplatin (80 mg/m^2 on day 1), 3-week cycle	73	NA	NA	73	NA	NA	NA	NA	NA
LOGiC	487	0	487	20	43	Lapatinib (1250mg) plus capecitabine (1700 mg/m^2) and oxaliplatin (130 mg/m^2), 3-week cycle	35	NA	NA	NA	NA	NA	NA	NA	NA
						Placebo plus capecitabine (1700 mg/m^2) and oxaliplatin (130 mg/m^2), 3-week cycle	28	NA	NA	NA	NA	NA	NA	NA	NA
RILOMET-1	609	0	609	77	124	Rilotumumab (15 mg/kg) plus epirubicin (50 mg/m^2), cisplatin (60 mg/m^2) and capecitabine (625 mg/m^2 orally twice daily), 3-week cycle	77	NA	NA	77	NA	NA	NA	NA	NA
						Placebo plus epirubicin (50 mg/m^2), cisplatin (60 mg/m^2) and capecitabine (625 mg/m^2 orally twice daily), 3-week cycle	110	NA	NA	110	NA	NA	NA	NA	NA
ToGA	584	0	584	0	106	Trastuzumab (8 mg/kg) plus capecitabine (1000 mg/m^2) and cisplatin (80 mg/m^2), 3-week cycle	58	NA	NA	NA	NA	NA	NA	NA	NA
						Placebo plus capecitabine (1000 mg/m2) and cisplatin (80 mg/m^2), 3-week cycle	48	NA	NA	NA	NA	NA	NA	NA	NA
REAL-3	553	0	545	217	169	Panitumumab (9 mg/kg) plus epirubicin (50 mg/m^2), oxaliplatin (130 mg/m^2) and capecitabine (1250 mg/m^2), 3-week cycle	200	NA	NA	NA	NA	NA	NA	NA	NA
						Epirubicin (50 mg/m^2), oxaliplatin (130 mg/m^2) and capecitabine (1250 mg/m^2), 3-week cycle	186	NA	NA	NA	NA	NA	NA	NA	NA

AVAGAST	774	0	774	0	103	Bevacizumab (7.5 mg/kg) plus capecitabine (1,000 mg/m2 twice daily) and cisplatin (80 mg/m2), 3-week cycle	54[§]	NA	NA	NA	NA	NA	NA	NA	NA
						Placebo plus capecitabine (1,000 mg/m2 twice daily) and cisplatin (80 mg/m2), 3-week cycle	49[§]	NA	NA	NA	NA	NA	NA	NA	NA
RAINFALL	645	0	645	0	163	Ramucirumab (8 mg/kg) plus fluorouracil (800 mg/m²) and cisplatin (80 mg/m²), 3-week cycle	83	NA	NA	83	NA	NA	NA	NA	NA
						Placebo plus fluorouracil (800 mg/m²) and cisplatin (80 mg/m²), 3-week cycle	80	NA	NA	80	NA	NA	NA	NA	NA
ASCO e16084[ɸ]	40	40	0	40	0	Camrelizumab plus fluorouracil and cisplatin	NA	NA	NA	NA	NA	NA	9	NA	NA
						Camrelizumab plus paclitaxel and cisplatin	NA	NA	NA	NA	NA	NA	24	NA	NA

ESCC: esophageal squamous cell carcinoma; EAC: esophageal adenocarcinoma; EC: esophageal cancer; GEJ: gastroesophageal junction.

¶ p value not been reported

§ HR not been reported, get data in figure

ɸ Conference report

Supplementary File 4. Cochrane risk of the bias assessment tool.

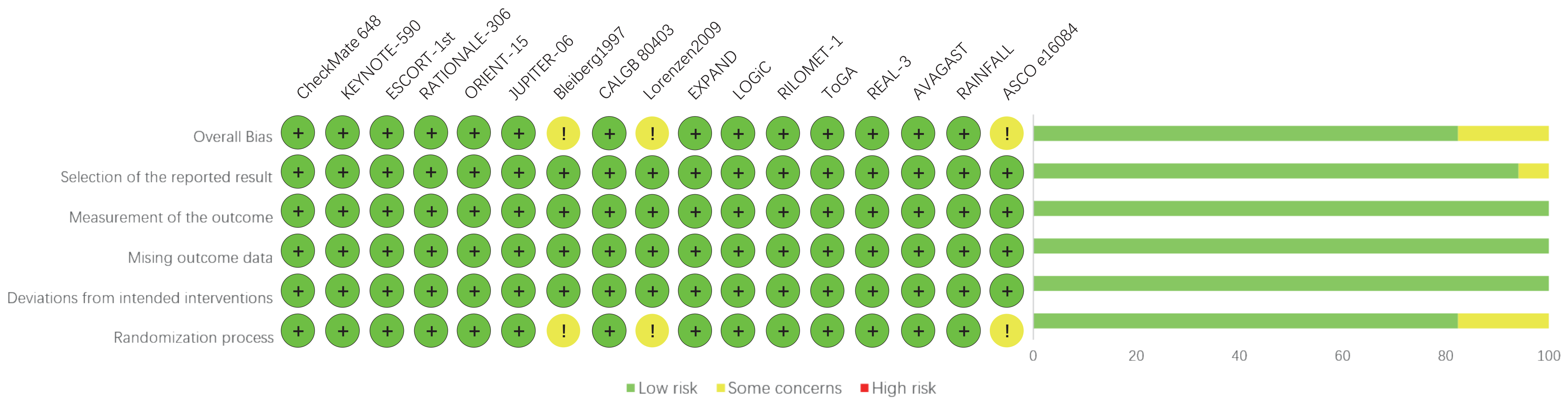

Supplementary File 5. 1-year survival profiles of the Bayesian network meta-analysis in patients with esophageal cancer.

1-year Progression-free Survival

1-year Overall Survival

Nivolumab +FbCT	0·96 (0·61, 1·51)	1·41 (0·89, 2·22)	2·32 (0·98, 5·47)	1·35 (0·63, 2·9)	2·38 (0·99, 5·69)	**0·67 (0·49, 0·92)**	0·54 (0·26, 1·09)	1·46 (0·4,5·39)	0·98 (0·21, 4·59)	0·56 (0·1, 3·2)
1 (0·8, 1·25)	Nivolumab +Ipilimumab	1·46 (0·93, 2·31)	**2·41 (1·02, 5·7)**	1·4 (0·65, 3·02)	**2·47 (1·03, 5·93)**	**0·7 (0·51, 0·96)**	0·56 (0·27, 1·13)	1·52 (0·42, 5·58)	1·02 (0·22, 4·77)	0·58 (0·1, 3·32)
0·96 (0·76, 1·2)	0·96 (0·77, 1·21)	Pembrolizumab +FbCT	1·65 (0·7, 3·91)	0·96 (0·44, 2·07)	1·69 (0·7, 4·07)	**0·47 (0·34, 0·66)**	**0·38 (0·19, 0·78)**	1·04 (0·28, 3·82)	0·69 (0·15, 3·25)	0·4 (0·07, 2·27)
1·23 (0·63, 2·4)	1·24 (0·63, 2·42)	1·29 (0·66, 2·51)	Camrelizumab +FfCT	0·58 (0·33, 1·02)	1·02 (0·5, 2·07)	**0·29 (0·13, 0·64)**	**0·23 (0·14, 0·38)**	0·63 (0·14, 2·8)	0·42 (0·08, 2·35)	0·24 (0·04, 1·6)
1·24 (0·64, 2·42)	1·25 (0·64, 2·43)	1·3 (0·67, 2·52)	1·01 (0·83, 1·23)	Sintilimab +FfCT	1·76 (0·97, 3·18)	**0·5 (0·25, 1)**	**0·4 (0·3, 0·53)**	1·08 (0·26, 4·57)	0·73 (0·14, 3·84)	0·41 (0·06, 2·65)
1·01 (0·51, 1·97)	1·01 (0·51, 1·98)	1·05 (0·53, 2·06)	0·81 (0·65, 1·01)	**0·81 (0·65, 1)**	Toripalimab +FfCT	**0·28 (0·12, 0·64)**	**0·23 (0·13, 0·38)**	0·62 (0·14, 2·77)	0·41 (0·07, 2·3)	0·24 (0·03, 1·58)
**1·22 (1·04, 1·43)**	**1·23 (1·05, 1·44)**	**1·27 (1·09, 1·5)**	0·99 (0·52, 1·9)	0·98 (0·51, 1·88)	1·22 (0·63, 2·34)	FbCT	0·8 (0·42, 1·51)	2·19 (0·62, 7·71)	1·46 (0·32, 6·65)	0·83 (0·15, 4·62)
1·53 (0·79, 2·93)	1·53 (0·8, 2·94)	1·59 (0·83, 3·06)	1·24 (1·07, 1·43)	**1·23 (1·08, 1·4)**	**1·52 (1·29, 1·79)**	1·25 (0·66, 2·35)	FfCT	2·73 (0·67, 11·2)	1·83 (0·35, 9·45)	1·04 (0·17, 6·49)
1·3 (0·62, 2·72)	1·31 (0·63, 2·73)	1·36 (0·65, 2·84)	1·06 (0·4, 2·79)	1·05 (0·4, 2·76)	1·3 (0·49, 3·44)	1·07 (0·52, 2·19)	0·85 (0·33, 2·23)	Cetuximab +FbCT	0·67 (0·29, 1·56)	0·38 (0·12, 1·23)
1·51 (0·66, 3·46)	1·52 (0·67, 3·47)	1·58 (0·69, 3·61)	1·23 (0·43, 3·47)	1·22 (0·43, 3·43)	1·51 (0·53, 4·27)	1·24 (0·55, 2·78)	0·99 (0·36, 2·77)	1·16 (0·8, 1·68)	Cetuximab +FfCT	0·57 (0·26, 1·28)
1·3 (0·53, 3·2)	1·3 (0·53, 3·22)	1·35 (0·55, 3·34)	1·05 (0·35, 3·16)	1·04 (0·35, 3·13)	1·29 (0·43, 3·9)	1·06 (0·44, 2·59)	0·85 (0·29, 2·53)	1 (0·59, 1·67)	0·86 (0·6, 1·23)	Cetuximab +OXbCT

FbCT, 5-FU-based chemotherapy; FfCT, 5-FU-free chemotherapy; OXbCT, Oxaliplatin-based chemotherapy.

Supplementary File 6. Overall adverse events of any grade among different treatment groups.

Symptoms (columns) / **Treatment regimen** (rows)

Treatment regimen	Nausea	Decreased appetite	Fatigue	Vomiting	WBC count decreased	Asthenia	Malaise	Neutropenia	Constipation	Anemia	Diarrhea	Alopecia	Hypothyroidism	Rash	Pruritus	Dysphagia	Renal disfunction	Liver disfunction
Nivolumab+FbCT	58·7	42·6	20·6	18·1	13·9		16·1	21·0	19·0	30·0	19·4	10·0	5·8	7·7	7·4		12·6	
Nivolumab+Ipilimumab	8·1	5·9	9·0	5·6	0·9		3·7	0·6	2·2	3·7	9·9	0·6	13·4	17·1	13·4		1·6	
Pembrolizumab+FbCT	63·0	39·2	36·5	29·7	24·1	12·2	11·6	36·5	13·5	38·6	26·2	13·8	10·3	7·8	6·2		18·1	4·9
Camrelizumab+FfCT	50·3	43·3		39·3	67·8	47·3		67·4		76·8		45·3	11·4	5·4	6·7		23·2	15·4
Sintilimab+FfCT	46·8	27·5		34·3	60·2	33·3		61·8		74·9	12·5	30·3	14·7	15·3			1·2	11·0
Toripalimab+FfCT	43·2	39·3	42·8	40·1	67·7			67·3	24·1	78·2	23·0	35·4		21·8		8·9	14·4	10·5
FbCT	52·4	33·3	23·0	22·7	13·8	4·7	11·2	22·6	17·2	33·8	18·4	9·5	2·9	3·1	1·3		13·6	2·5
FfCT	47·6	38·7	10·9	34·0	59·0	23·5		56·6	5·9	75·7	7·0	36·5	4·5	5·1	0·8	3·5	9·1	8·9
Cetuximab+FbCT	22·2		20·2	15·2	9·1			57·6		32·3	13·1			22·2				
Cetuximab+FfCT					21·9			58·9		13·7								
Cetuximab+OXbCT					16·4			46·6		6·8								

The heatmap depicts the frequencies of each adverse event during treatment courses among different treatment groups. The statistics in each cell represents the hazard or odds ratios (95% confidential intervals) when comparing the column-defining regimen to the row-defining regimen.

Supplmentary File 7. Bayesian ranking results (SUCRA value) of network meta-analysis.

Treatment	Rank of possibility (SUCRA value)																	
	1	2	3	4	5	6	7	8	9	10	11	12	13	14	15	16	17	18
**Overall survival for esophageal cancer patients**																		
Nivolumab+FbCT	0·008	0·021	0·034	0·046	0·056	0·067	0·078	0·089	0·097	0·102	0·104	0·098	0·081	0·058	0·036	0·019	0·005	0·001
Nivolumab+Ipilimumab	0·003	0·008	0·016	0·025	0·036	0·047	0·060	0·074	0·090	0·106	0·118	0·121	0·110	0·084	0·057	0·033	0·008	0·002
Pembrolizumab+FbCT	0·002	0·009	0·022	0·038	0·055	0·074	0·093	0·111	0·122	0·128	0·120	0·099	0·068	0·038	0·016	0·005	0·000	0·000
Camrelizumab+FfCT	0·010	0·031	0·059	0·086	0·107	0·116	0·116	0·109	0·099	0·085	0·070	0·053	0·033	0·017	0·007	0·002	0·001	0·000
Tislelizumab+FbCT	0·139	0·066	0·093	0·106	0·112	0·113	0·109	0·101	0·088	0·070	0·051	0·032	0·017	0·008	0·003	0·001	0·000	0·000
Sintilimab+FfCT	0·241	0·099	0·146	0·161	0·147	0·120	0·094	0·068	0·049	0·034	0·022	0·013	0·006	0·002	0·001	0·000	0·000	0·000
Toripalimab+FfCT	0·290	0·201	0·184	0·134	0·097	0·071	0·053	0·039	0·029	0·021	0·014	0·009	0·005	0·002	0·001	0·000	0·000	0·000
FbCT	0·000	0·000	0·000	0·000	0·000	0·000	0·000	0·001	0·002	0·006	0·015	0·037	0·077	0·133	0·192	0·251	0·230	0·057
FfCT	0·000	0·000	0·000	0·000	0·000	0·000	0·000	0·000	0·001	0·004	0·013	0·038	0·093	0·185	0·261	0·235	0·131	0·038
Cetuximab+FbCT	0·029	0·172	0·095	0·067	0·054	0·046	0·041	0·036	0·033	0·031	0·029	0·026	0·022	0·017	0·014	0·012	0·010	0·007
Cetuximab+FfCT	0·002	0·012	0·031	0·044	0·055	0·066	0·074	0·081	0·087	0·091	0·098	0·099	0·089	0·067	0·046	0·031	0·019	0·008
Cetuximab+OXbCT	0·037	0·129	0·073	0·054	0·045	0·040	0·037	0·036	0·034	0·034	0·035	0·036	0·035	0·031	0·028	0·030	0·037	0·043
Lapatinib+FfCT	0·107	0·063	0·045	0·035	0·031	0·029	0·028	0·028	0·030	0·032	0·034	0·038	0·042	0·044	0·045	0·054	0·090	0·228
Rilotumumab+FfCT	0·020	0·026	0·028	0·028	0·029	0·030	0·033	0·035	0·039	0·045	0·050	0·058	0·066	0·069	0·072	0·085	0·124	0·163
Trastuzumab+FfCT	0·084	0·104	0·098	0·085	0·076	0·069	0·064	0·061	0·058	0·056	0·053	0·050	0·045	0·034	0·024	0·018	0·013	0·008
Panitumumab+FfCT	0·018	0·036	0·049	0·057	0·064	0·067	0·071	0·074	0·076	0·078	0·081	0·081	0·077	0·061	0·044	0·031	0·023	0·012
Bevacizumab+FfCT	0·011	0·021	0·027	0·032	0·037	0·042	0·047	0·052	0·059	0·065	0·073	0·084	0·091	0·088	0·077	0·072	0·072	0·051
Ramucirumab+FbCT	0·000	0·000	0·000	0·001	0·001	0·002	0·003	0·005	0·008	0·012	0·018	0·029	0·043	0·061	0·078	0·120	0·235	0·384
**Progression-free survival for esophageal cancer patients**																		
Nivolumab+FbCT	0·002	0·006	0·014	0·025	0·048	0·131	0·213	0·223	0·159	0·092	0·057	0·023	0·007					
Pembrolizumab+FbCT	0·048	0·085	0·101	0·152	0·217	0·204	0·125	0·050	0·015	0·004	0·001	0·000	0·000					
Camrelizumab+FfCT	0·210	0·237	0·213	0·146	0·097	0·057	0·027	0·010	0·003	0·000	0·000	0·000	0·000					
Tislelizumab+FbCT	0·114	0·118	0·127	0·182	0·198	0·147	0·077	0·029	0·008	0·002	0·000	0·000	0·000					

13

Sintilimab+FfCT	0·211	0·239	0·211	0·146	0·097	0·057	0·027	0·010	0·003	0·001	0·000	0·000	0·000
Toripalimab+FfCT	0·158	0·184	0·204	0·160	0·123	0·087	0·052	0·022	0·007	0·002	0·000	0·000	0·000
FbCT	0·000	0·000	0·000	0·000	0·000	0·001	0·019	0·078	0·163	0·219	0·221	0·203	0·095
FfCT	0·000	0·000	0·000	0·000	0·001	0·007	0·030	0·083	0·159	0·225	0·238	0·181	0·076
Cetuximab+FbCT	0·069	0·050	0·048	0·067	0·081	0·110	0·139	0·127	0·094	0·069	0·059	0·049	0·037
Cetuximab+FfCT	0·000	0·000	0·000	0·001	0·004	0·011	0·027	0·062	0·100	0·122	0·156	0·230	0·286
Cetuximab+OXbCT	0·150	0·053	0·047	0·063	0·063	0·078	0·095	0·093	0·072	0·060	0·060	0·074	0·091
Rilotumumab+FfCT	0·034	0·025	0·028	0·048	0·055	0·076	0·101	0·110	0·099	0·087	0·085	0·094	0·158
Ramucirumab+FbCT	0·003	0·004	0·006	0·010	0·015	0·036	0·069	0·102	0·118	0·119	0·122	0·147	0·251
**Objective response rate for esophageal cancer patients**													
Nivolumab_FbCT	0·647	0·188	0·091	0·047	0·018	0·008	0·001	0·001	0·000	0·000	0·000	0·000	0·000
Nivolumab_Ipilimumab	0·000	0·001	0·006	0·203	0·167	0·139	0·187	0·152	0·052	0·039	0·027	0·018	0·009
Pembrolizumab_FbCT	0·122	0·348	0·302	0·100	0·069	0·045	0·007	0·004	0·001	0·001	0·000	0·000	0·000
Camrelizumab_FbCT	0·002	0·002	0·002	0·006	0·006	0·010	0·014	0·019	0·036	0·050	0·075	0·120	0·659
Camrelizumab_FfCT	0·000	0·001	0·005	0·006	0·011	0·035	0·035	0·067	0·089	0·121	0·545	0·083	0·000
Tislelizumab_FbCT	0·055	0·273	0·403	0·116	0·080	0·058	0·009	0·004	0·002	0·001	0·000	0·000	0·000
Sintilimab_FfCT	0·020	0·019	0·021	0·071	0·061	0·088	0·109	0·097	0·393	0·111	0·010	0·001	0·000
Toripalimab_FfCT	0·004	0·012	0·013	0·026	0·054	0·055	0·086	0·100	0·167	0·406	0·069	0·008	0·000
FbCT	0·000	0·000	0·000	0·091	0·202	0·158	0·201	0·195	0·059	0·044	0·027	0·016	0·006
FfCT	0·000	0·000	0·000	0·001	0·002	0·003	0·016	0·019	0·043	0·066	0·074	0·576	0·200
Cetuximab_FbCT	0·082	0·086	0·073	0·202	0·137	0·156	0·082	0·049	0·039	0·041	0·040	0·012	0·001
Cetuximab_FfCT	0·001	0·011	0·033	0·032	0·060	0·142	0·107	0·210	0·065	0·069	0·072	0·099	0·098
Cetuximab_OXbCT	0·067	0·059	0·051	0·100	0·133	0·103	0·144	0·084	0·053	0·052	0·060	0·067	0·027
**Adverse events Grade no less than 3 for esophageal cancer patients**													
Nivolumab_FbCT	0·000	0·000	0·000	0·000	0·001	0·002	0·004	0·017	0·794	0·076	0·030	0·075	
Nivolumab_Ipilimumab	0·008	0·004	0·003	0·007	0·731	0·083	0·066	0·084	0·010	0·003	0·002	0·000	
Pembrolizumab_FbCT	0·000	0·001	0·002	0·002	0·026	0·129	0·377	0·414	0·028	0·012	0·009	0·000	
Camrelizumab_FfCT	0·805	0·117	0·058	0·016	0·002	0·001	0·000	0·000	0·000	0·000	0·000	0·000	
Tislelizumab_FbCT	0·000	0·001	0·001	0·002	0·080	0·211	0·237	0·409	0·036	0·011	0·011	0·000	

14

Sintilimab_FfCT	0·026	0·068	0·221	0·665	0·008	0·002	0·003	0·004	0·002	0·000	0·000	0·000
Toripalimab_FfCT	0·063	0·165	0·474	0·285	0·004	0·002	0·003	0·002	0·001	0·000	0·000	0·000
FbCT	0·000	0·003	0·003	0·002	0·114	0·535	0·282	0·044	0·011	0·004	0·000	0·000
FfCT	0·096	0·641	0·237	0·020	0·001	0·002	0·002	0·000	0·000	0·000	0·000	0·000
Cetuximab_FbCT	0·000	0·000	0·000	0·000	0·003	0·008	0·009	0·006	0·020	0·233	0·448	0·273
Cetuximab_FfCT	0·000	0·000	0·000	0·000	0·001	0·006	0·008	0·005	0·007	0·051	0·327	0·595
Cetuximab_OXbCT	0·001	0·000	0·000	0·001	0·029	0·018	0·009	0·013	0·091	0·609	0·172	0·056

15

Supplementary File 8. Heterogeneity test

Comparison	Data points	Ratio	I^2
OS	18	0·9648	2%
PFS	13	0·9381	2%
1 year OS	10	0·9981	10%
1 year PFS	9	1	11%
ORR	12	0·9989	8%
DCR	11	0·9986	9%
AEs ≥ Grade 3	11	0·9995	9%
ESCC_OS	9	1	11%
ESCC_PFS	8	0·9983	12%
ESCC_ORR	10	0·9996	10%
ESCC_AE	9	0·9997	11%

16

Supplementary File 9. Forest plots for (A) pooled (pair-wise, and network) and (B) indirect (back-calculated) analysis.

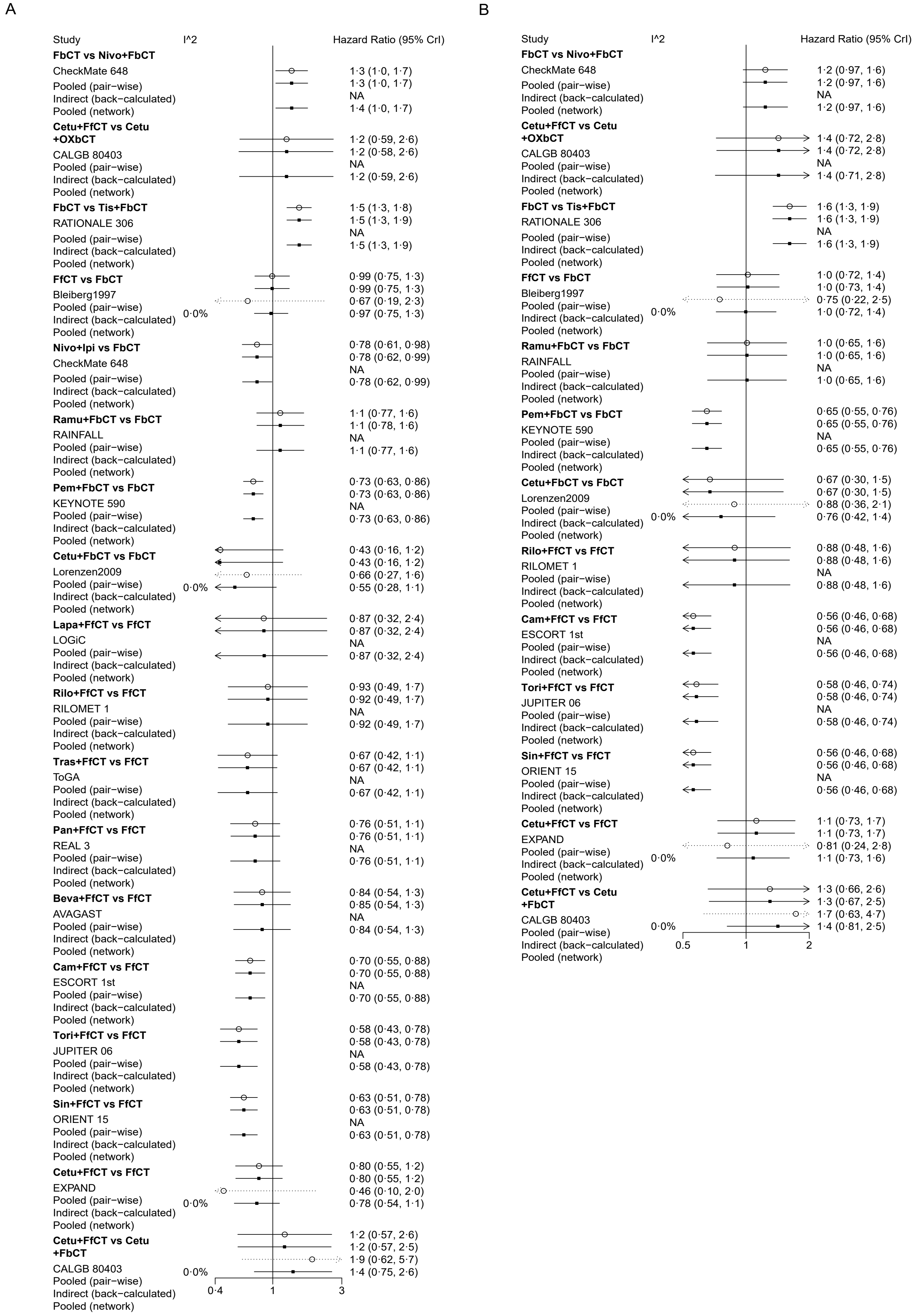

Supplementary File 10. Inconsistency test.

Comparison	Direct effect	Indirect effect	Overall network	P value
Cetuximab+FbCT vs Cetuximab+FfCT	0·26 (-0·41, 0·94)	0·54 (-0·44, 1·5)	0·34 (-0·21, 0·91)	0·65005
Cetuximab+FbCT vs FbCT	0·40 (-0·41, 1·2)	0·13 (-0·73, 0·99)	0·27 (-0·32, 0·86)	0·6552
FbCT vs FfCT	0·020 (-0·32, 0·37)	-0·25 (-1·4, 0·89)	-0·0024 (-0·33, 0·32)	0·65865
Cetuximab+FfCT vs FfCT	-0·11 (-0·54, 0·31)	0·16 (-0·94, 1·3)	-0·078 (-0·48, 0·32)	0·65605

Supplementary File 11. Transitivity assessment.

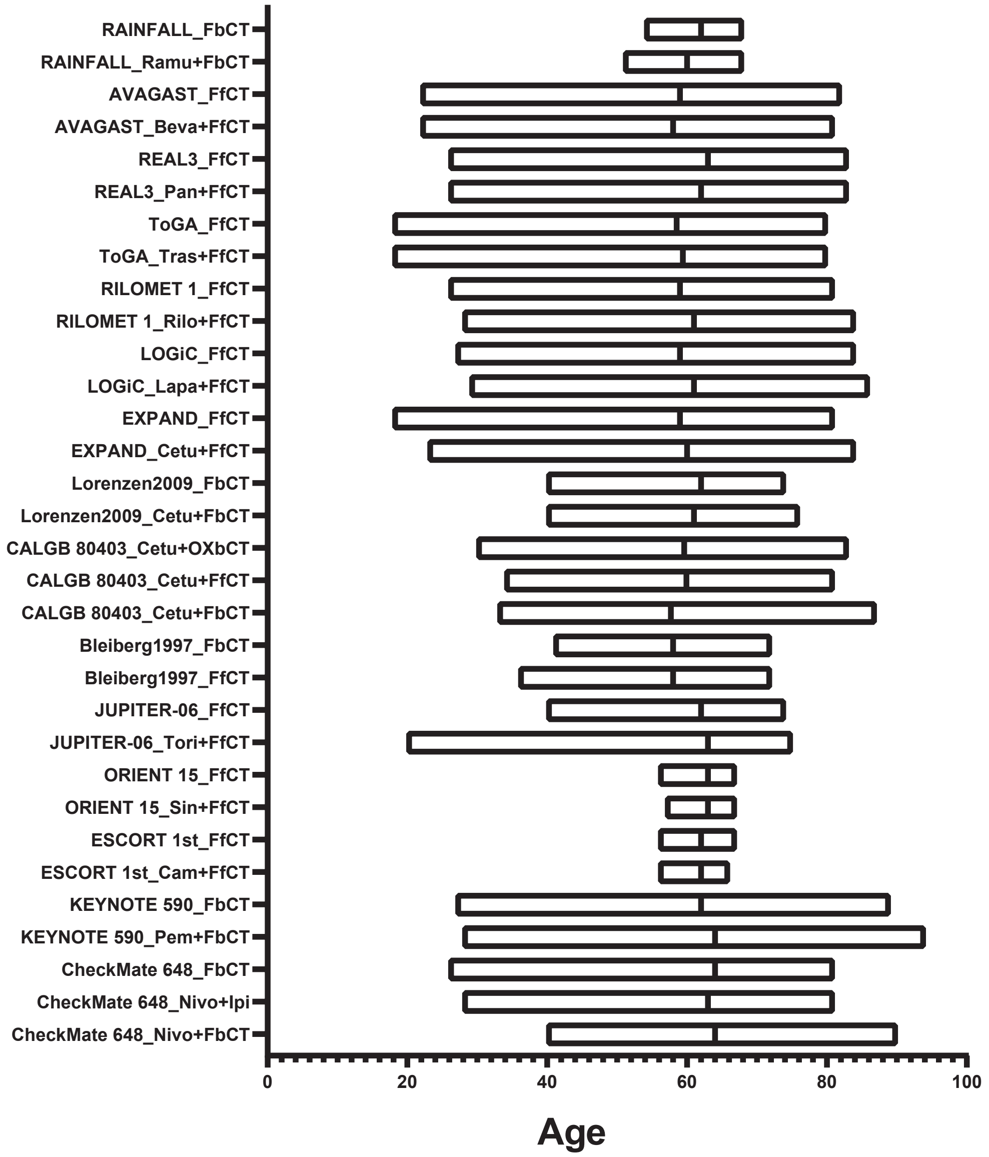

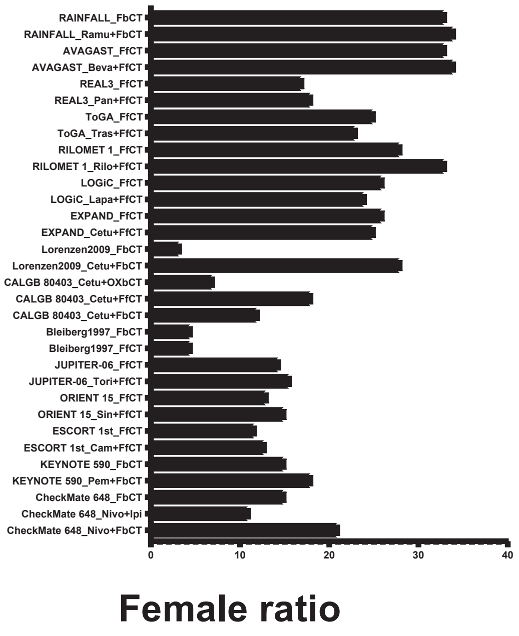

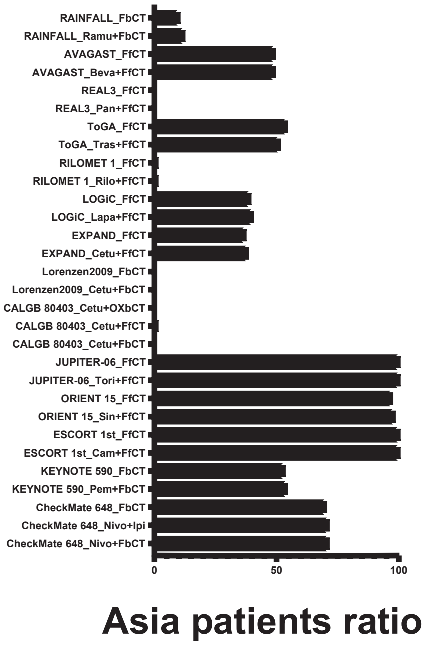

Supplementary File 12. Node-splitting analysis of inconsistency. Node-splitting analysis of inconsistency in OS (A) and PFS (B).

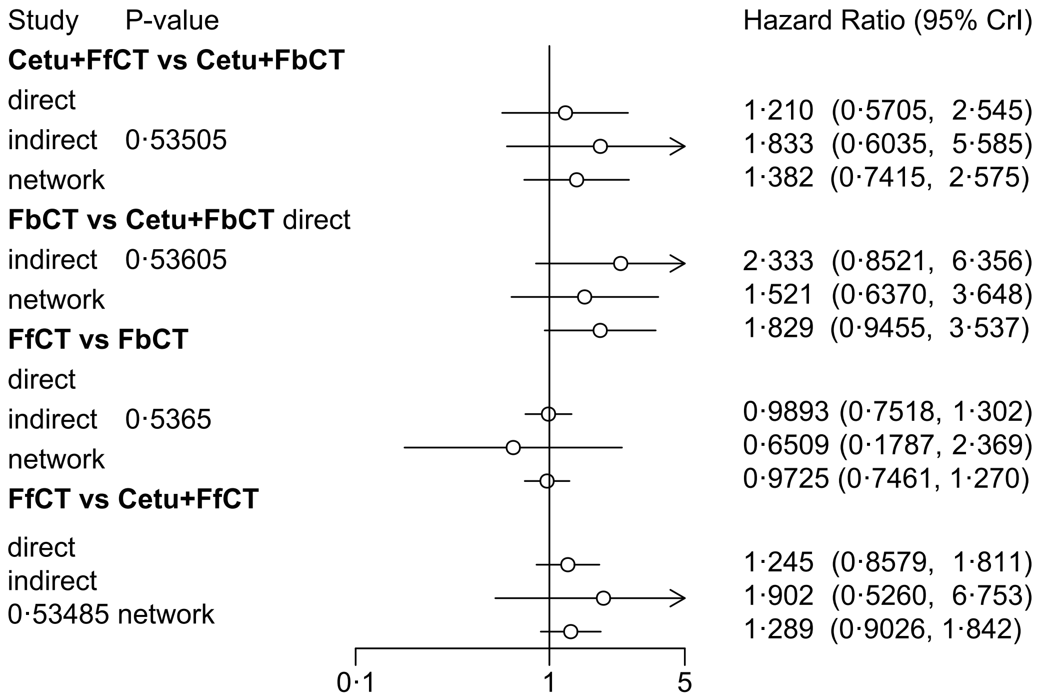

A

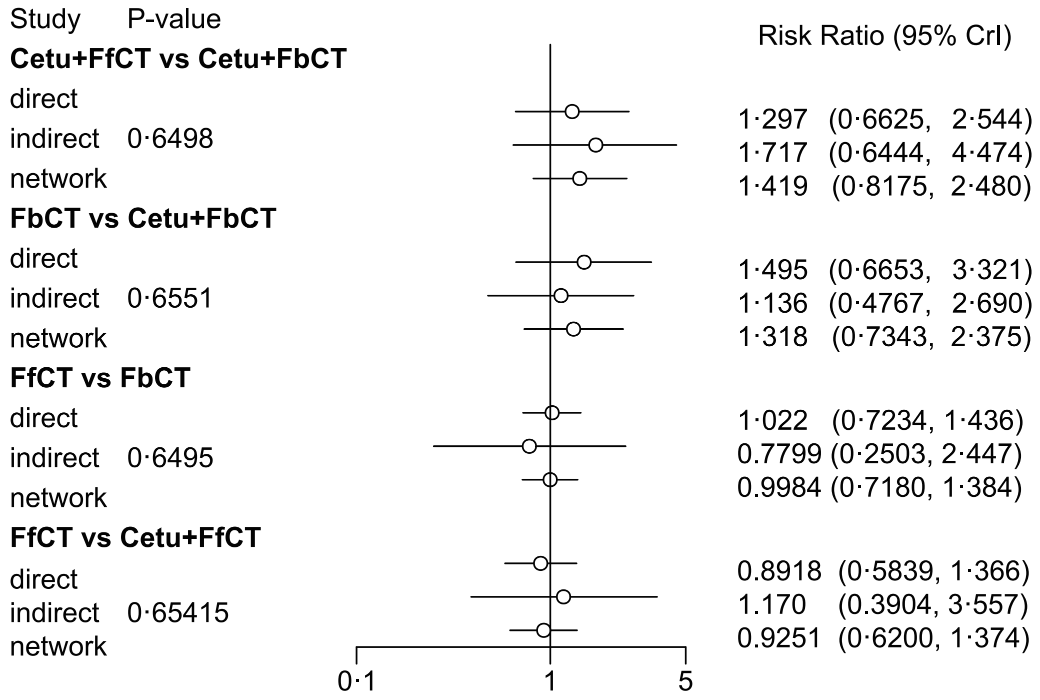

B

Supplementary File 13. The forest plots for main network meta-analysis results. (A) Overall survival (OS). (B) Progression-free survival (PFS). (C) Objective response rate (ORR). (D) Adverse events of any grade and ≥ 3 grade.

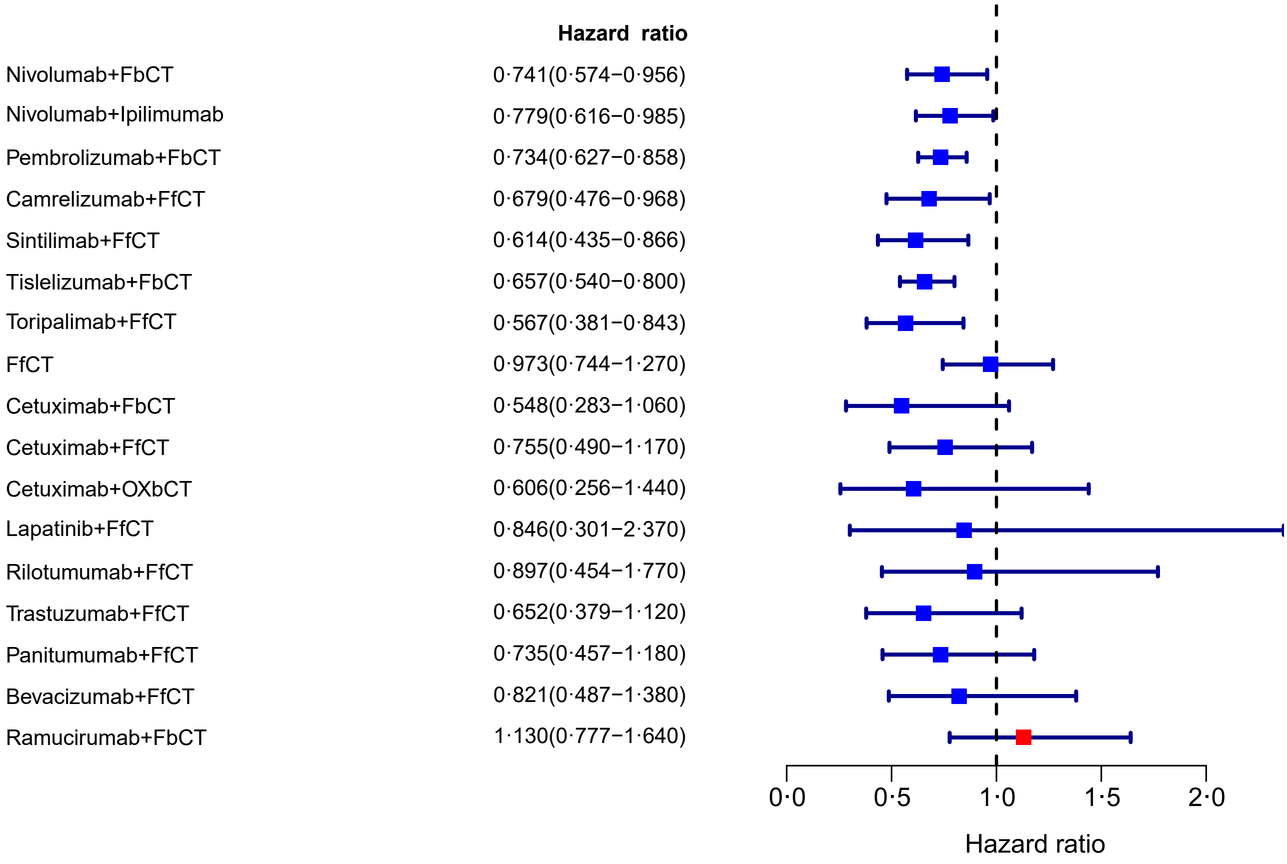

A

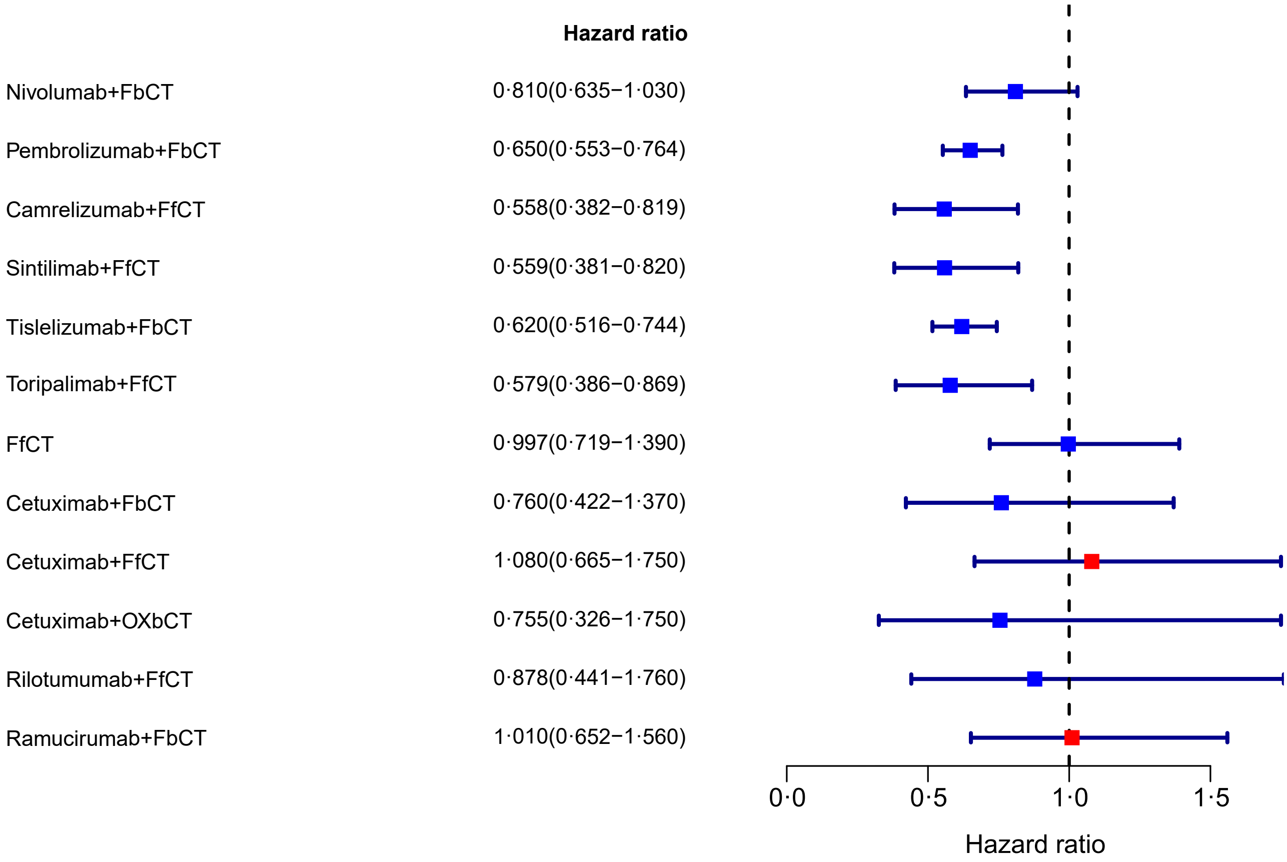

B

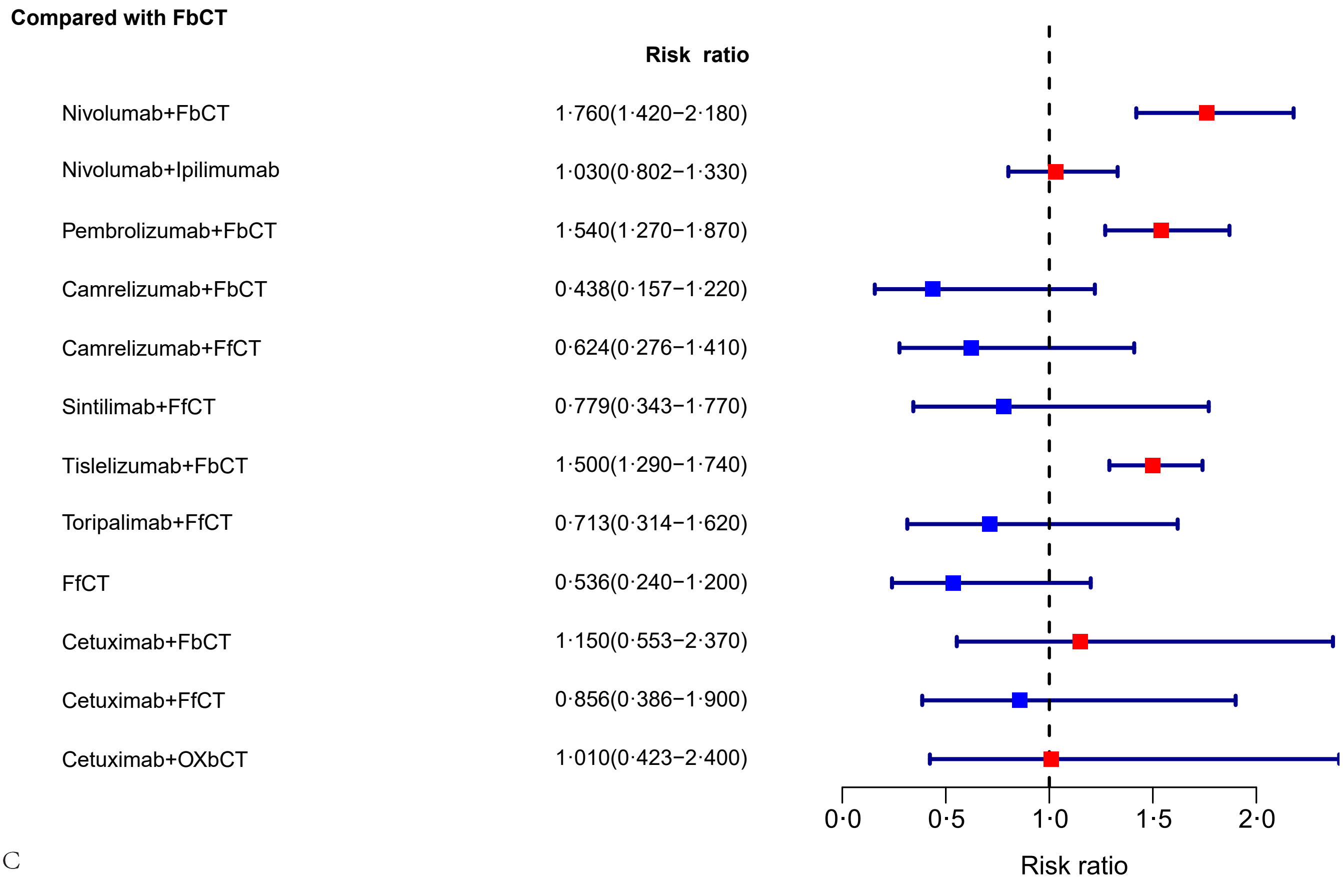
Compared with FbCT
Risk ratio
Nivolumab+FbCT 1·760(1·420−2·180)
Nivolumab+Ipilimumab 1·030(0·802−1·330)
Pembrolizumab+FbCT 1·540(1·270−1·870)
Camrelizumab+FbCT 0·438(0·157−1·220)
Camrelizumab+FfCT 0·624(0·276−1·410)
Sintilimab+FfCT 0·779(0·343−1·770)
Tislelizumab+FbCT 1·500(1·290−1·740)
Toripalimab+FfCT 0·713(0·314−1·620)
FfCT 0·536(0·240−1·200)
Cetuximab+FbCT 1·150(0·553−2·370)
Cetuximab+FfCT 0·856(0·386−1·900)
Cetuximab+OXbCT 1·010(0·423−2·400)
0·0
0·5
1·0
1·5
2·0
Risk ratio

C

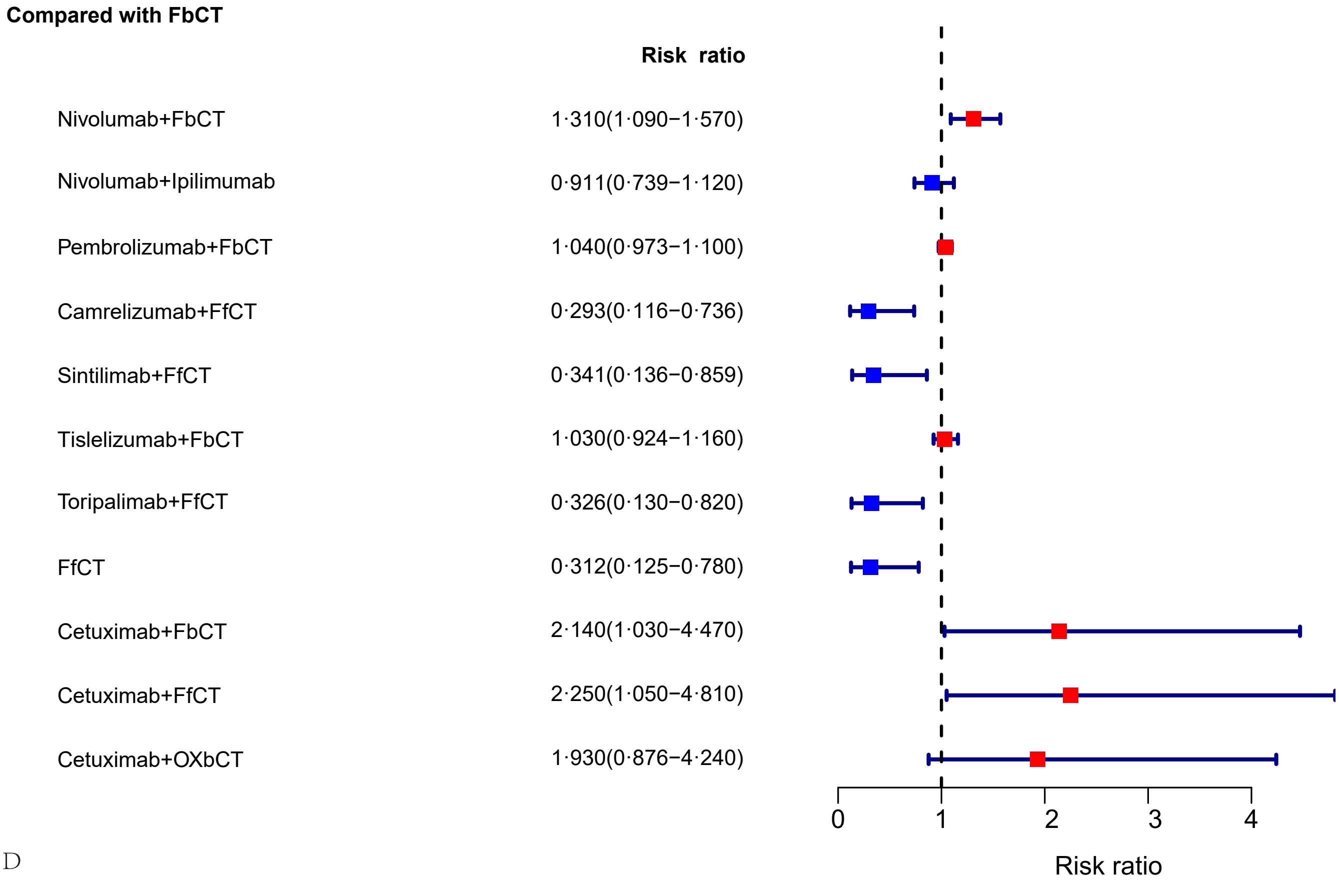
Compared with FbCT
Risk ratio
Nivolumab+FbCT 1·310(1·090−1·570)
Nivolumab+Ipilimumab 0·911(0·739−1·120)
Pembrolizumab+FbCT 1·040(0·973−1·100)
Camrelizumab+FfCT 0·293(0·116−0·736)
Sintilimab+FfCT 0·341(0·136−0·859)
Tislelizumab+FbCT 1·030(0·924−1·160)
Toripalimab+FfCT 0·326(0·130−0·820)
FfCT 0·312(0·125−0·780)
Cetuximab+FbCT 2·140(1·030−4·470)
Cetuximab+FfCT 2·250(1·050−4·810)
Cetuximab+OXbCT 1·930(0·876−4·240)
0
1
2
3
4
Risk ratio

D

Supplementary File 14. Trace and density plot.

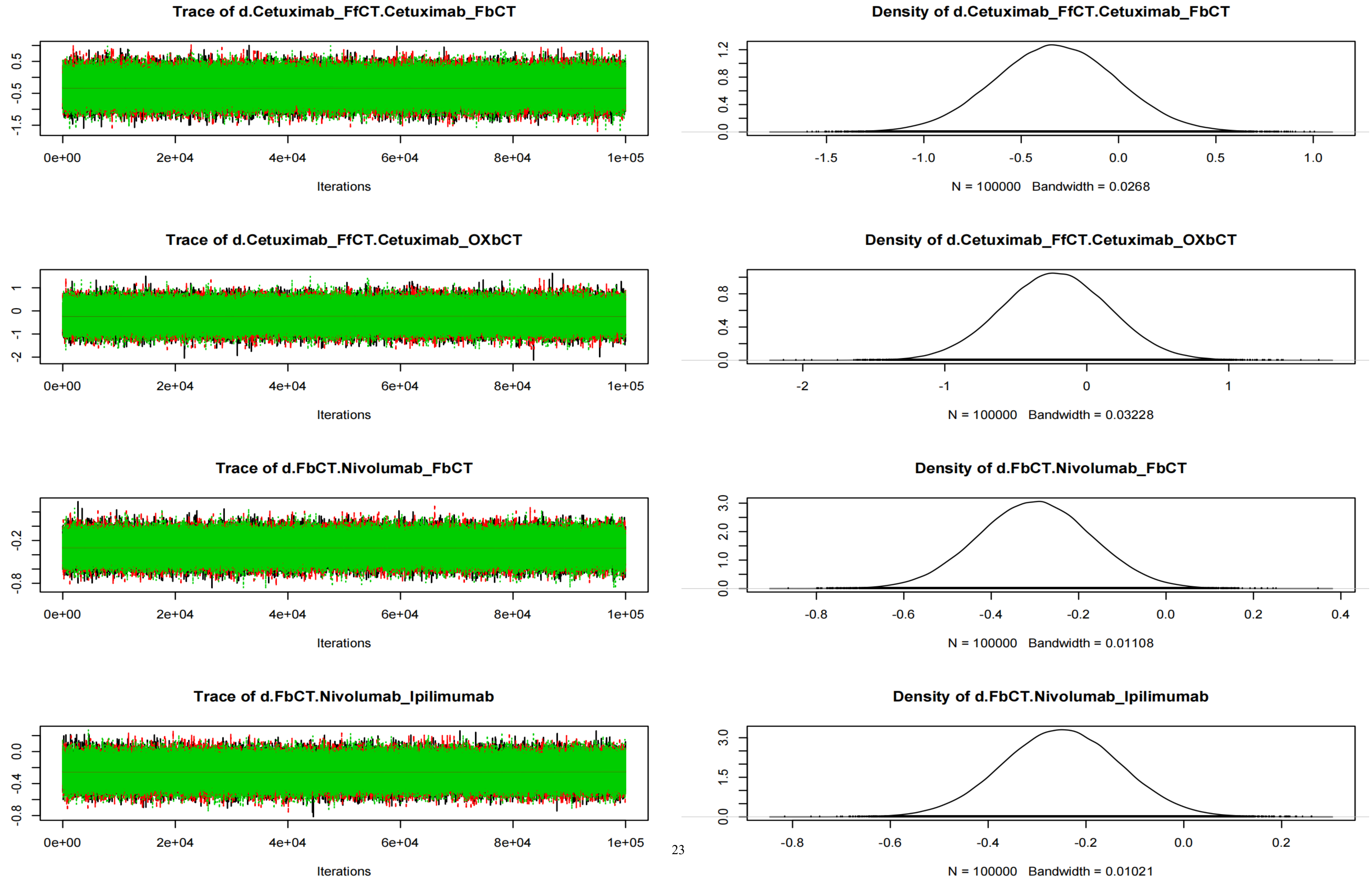

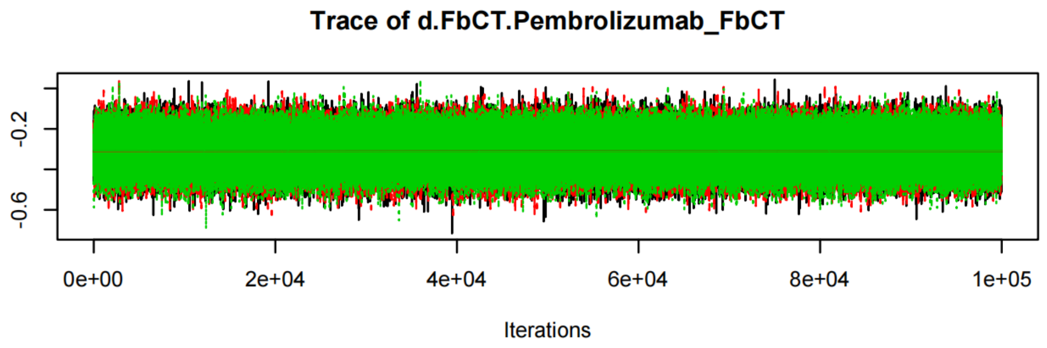
Trace of d.FbCT.Pembrolizumab_FbCT
-0.2
-0.6
0e+00
2e+04
4e+04
6e+04
8e+04
1e+05
Iterations

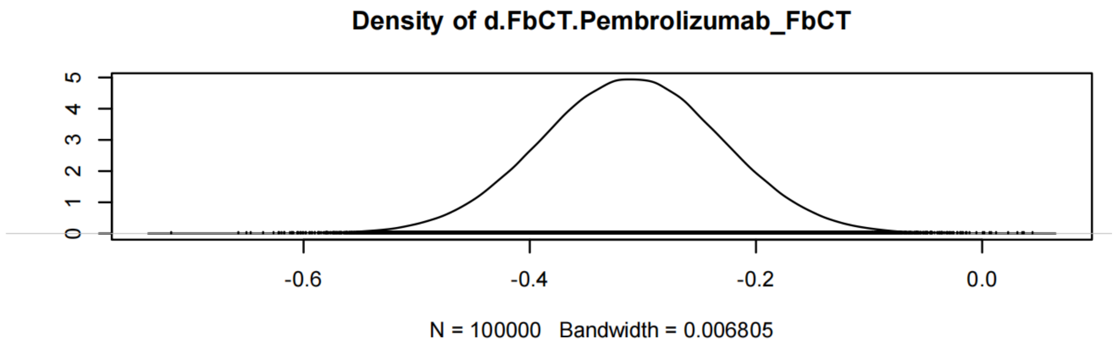
Density of d.FbCT.Pembrolizumab_FbCT
0
1
2
3
4
5
-0.6
-0.4
-0.2
0.0
N = 100000 Bandwidth = 0.006805

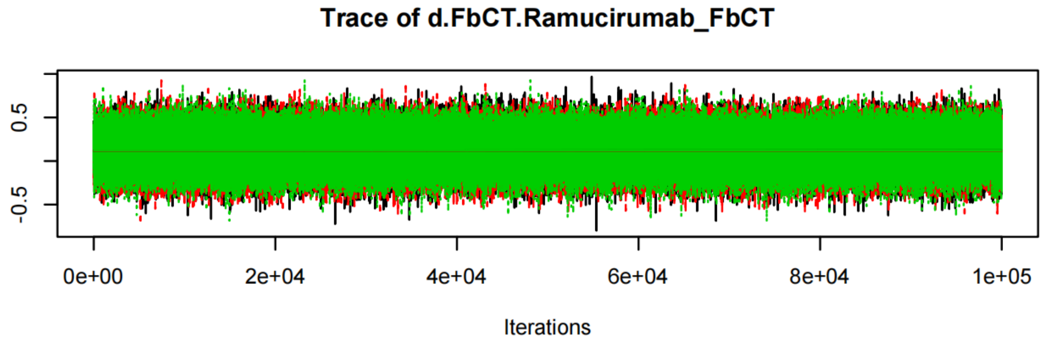
Trace of d.FbCT.Ramucirumab_FbCT
0.5
-0.5
0e+00
2e+04
4e+04
6e+04
8e+04
1e+05
Iterations

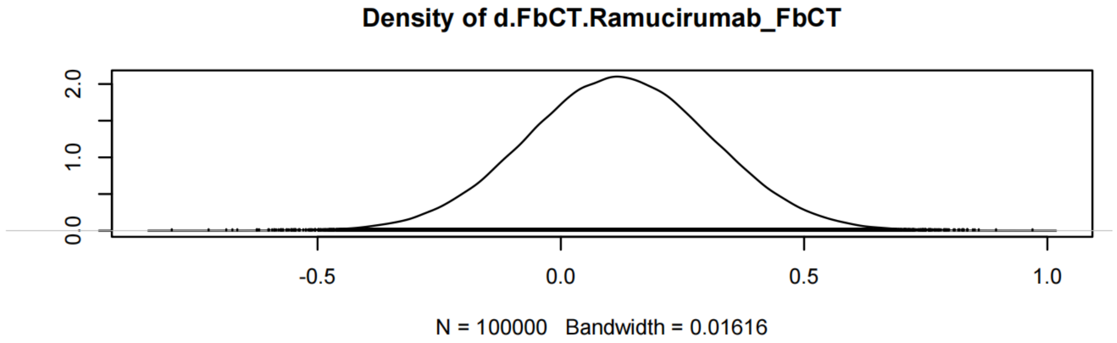
Density of d.FbCT.Ramucirumab_FbCT
0.0
1.0
2.0
-0.5
0.0
0.5
1.0
N = 100000 Bandwidth = 0.01616

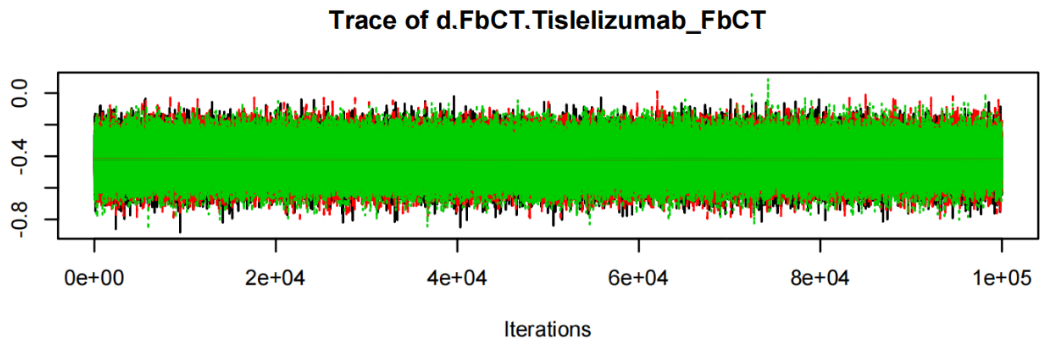
Trace of d.FbCT.Tislelizumab_FbCT
0.0
-0.4
-0.8
0e+00
2e+04
4e+04
6e+04
8e+04
1e+05
Iterations

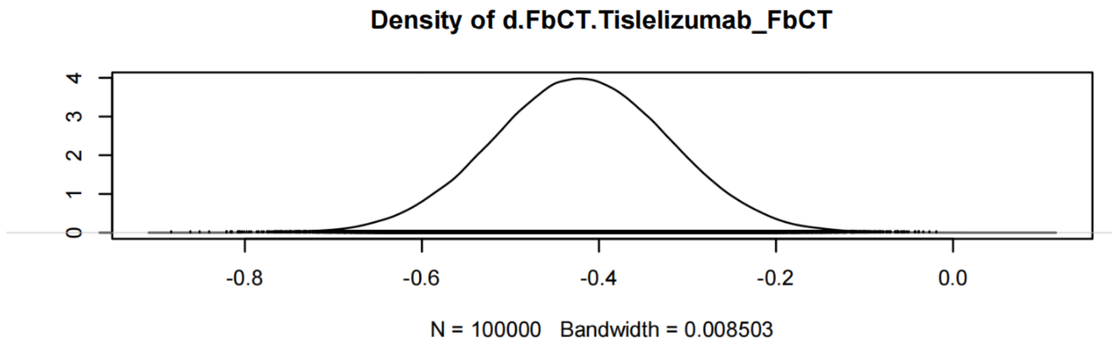
Density of d.FbCT.Tislelizumab_FbCT
0
1
2
3
4
-0.8
-0.6
-0.4
-0.2
0.0
N = 100000 Bandwidth = 0.008503

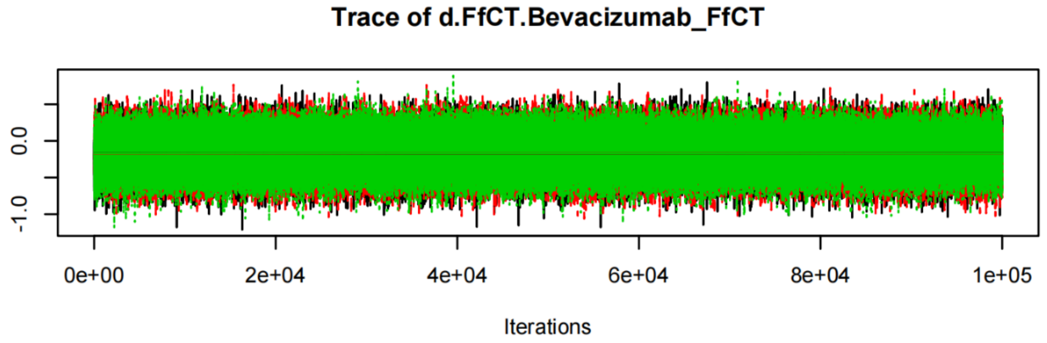
Trace of d.FfCT.Bevacizumab_FfCT
0.0
-1.0
0e+00
2e+04
4e+04
6e+04
8e+04
1e+05
Iterations

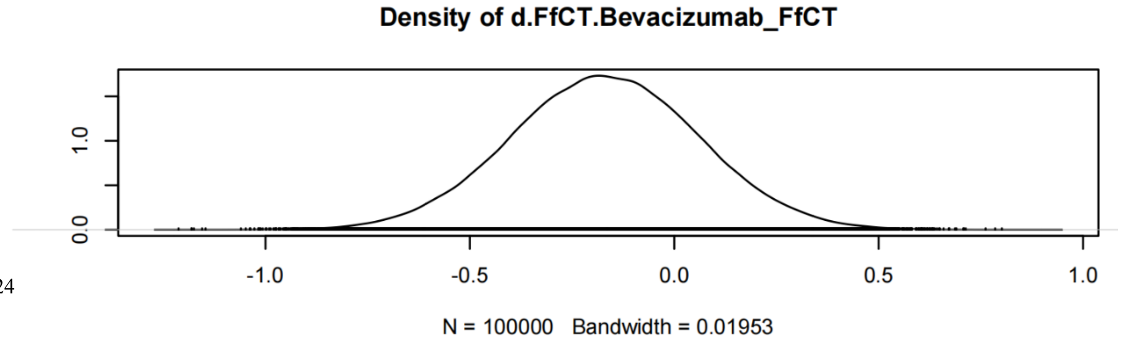
Density of d.FfCT.Bevacizumab_FfCT
0.0
1.0
-1.0
-0.5
0.0
0.5
1.0
N = 100000 Bandwidth = 0.01953

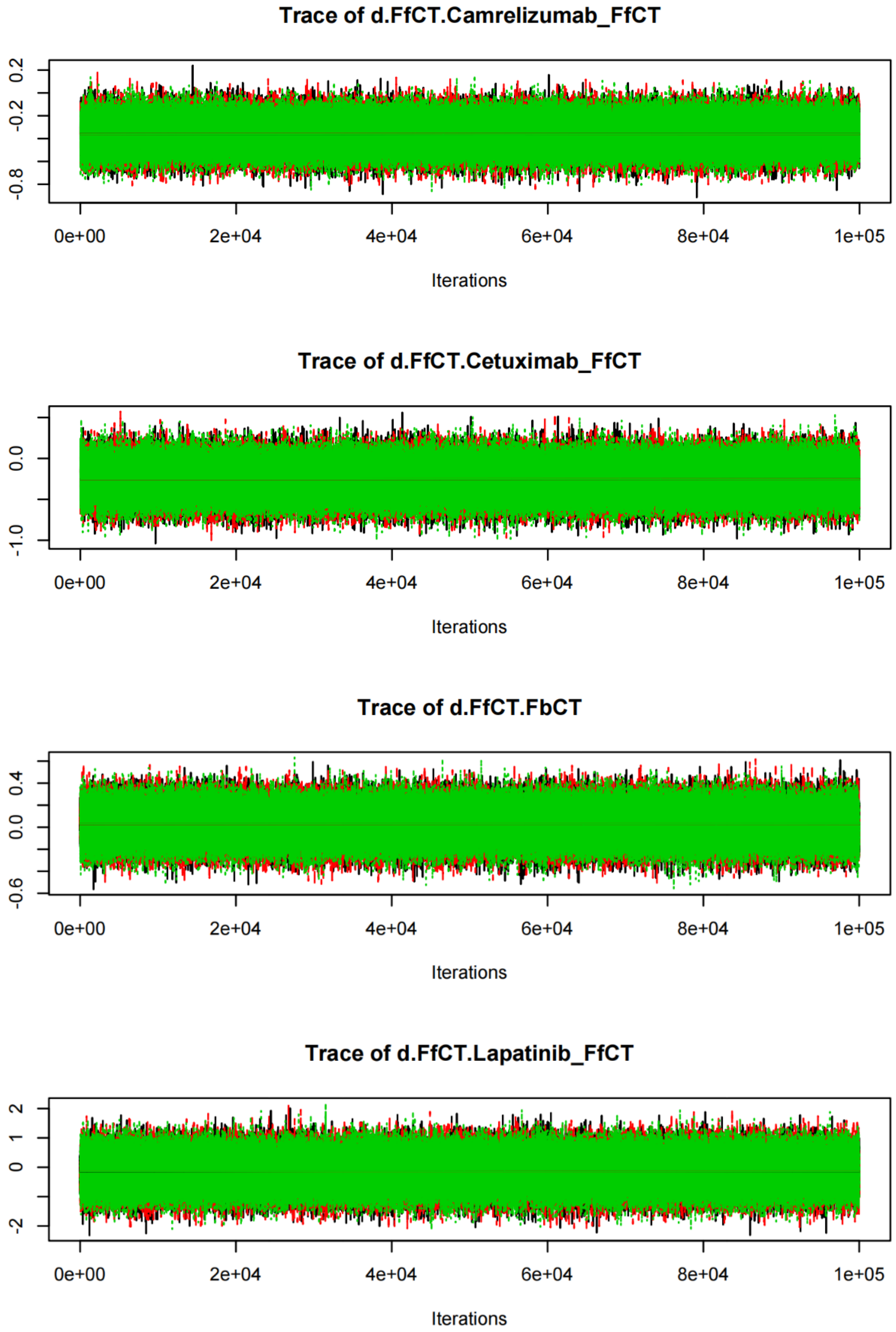
Trace of d.FfCT.Camrelizumab_FfCT
Iterations
Trace of d.FfCT.Cetuximab_FfCT
Iterations
Trace of d.FfCT.FbCT
Iterations
Trace of d.FfCT.Lapatinib_FfCT
Iterations
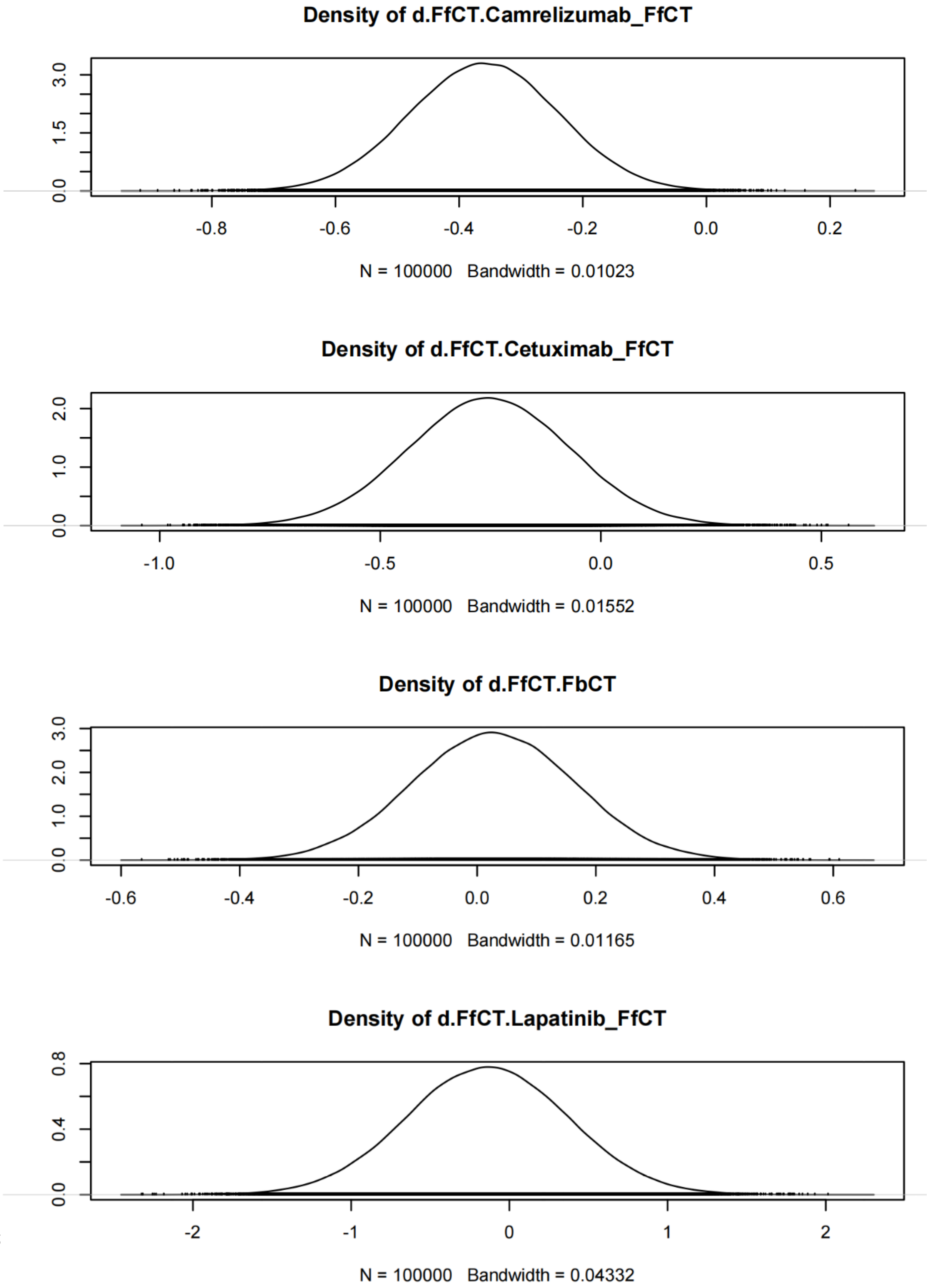
Density of d.FfCT.Camrelizumab_FfCT
N = 100000 Bandwidth = 0.01023
Density of d.FfCT.Cetuximab_FfCT
N = 100000 Bandwidth = 0.01552
Density of d.FfCT.FbCT
N = 100000 Bandwidth = 0.01165
Density of d.FfCT.Lapatinib_FfCT
N = 100000 Bandwidth = 0.04332

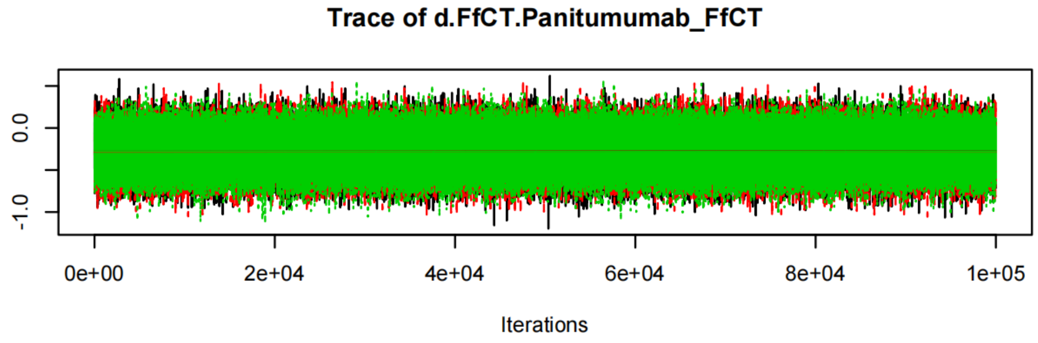
Trace of d.FfCT.Panitumumab_FfCT
0.0
-1.0
0e+00
2e+04
4e+04
6e+04
8e+04
1e+05
Iterations
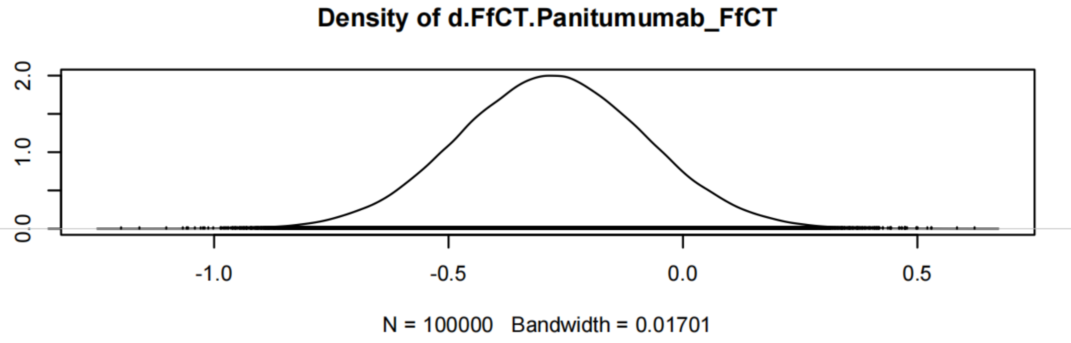
Density of d.FfCT.Panitumumab_FfCT
0.0
1.0
2.0
-1.0
-0.5
0.0
0.5
N = 100000 Bandwidth = 0.01701
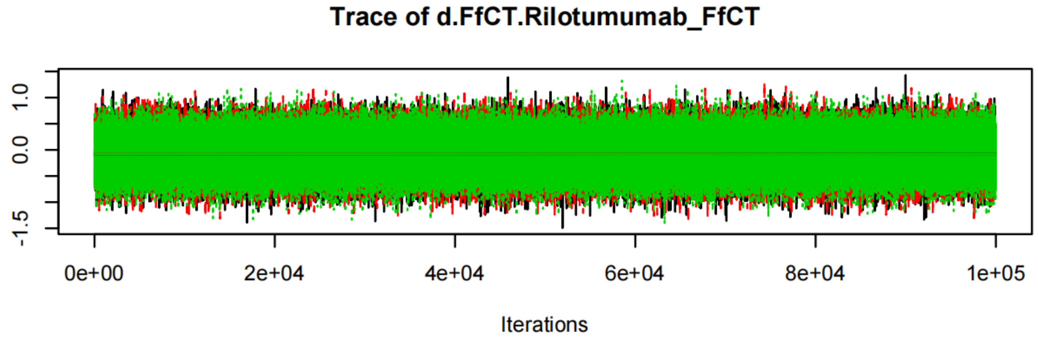
Trace of d.FfCT.Rilotumumab_FfCT
1.0
0.0
-1.5
0e+00
2e+04
4e+04
6e+04
8e+04
1e+05
Iterations
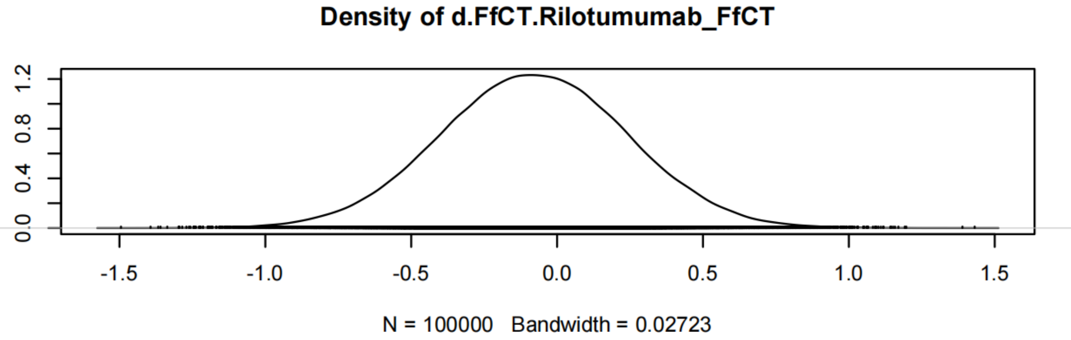
Density of d.FfCT.Rilotumumab_FfCT
0.0
0.4
0.8
1.2
-1.5
-1.0
-0.5
0.0
0.5
1.0
1.5
N = 100000 Bandwidth = 0.02723
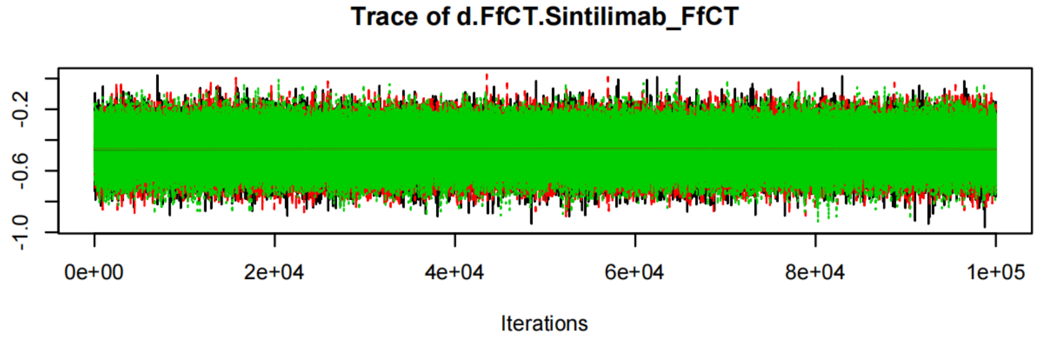
Trace of d.FfCT.Sintilimab_FfCT
-0.2
-0.6
-1.0
0e+00
2e+04
4e+04
6e+04
8e+04
1e+05
Iterations
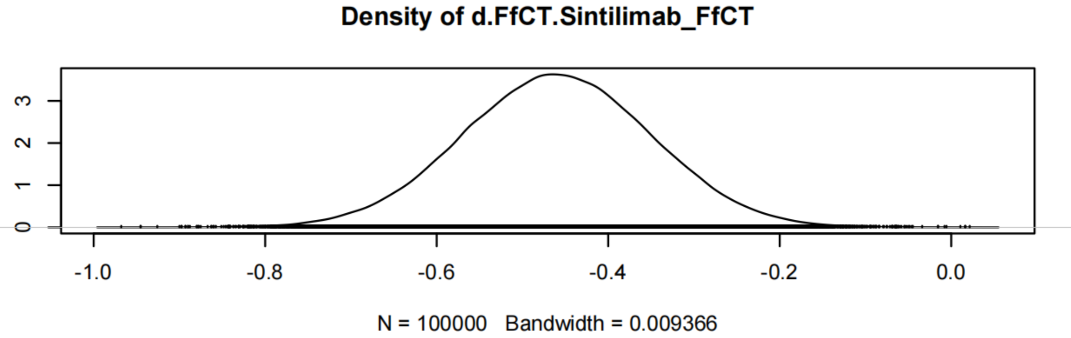
Density of d.FfCT.Sintilimab_FfCT
0
1
2
3
-1.0
-0.8
-0.6
-0.4
-0.2
0.0
N = 100000 Bandwidth = 0.009366
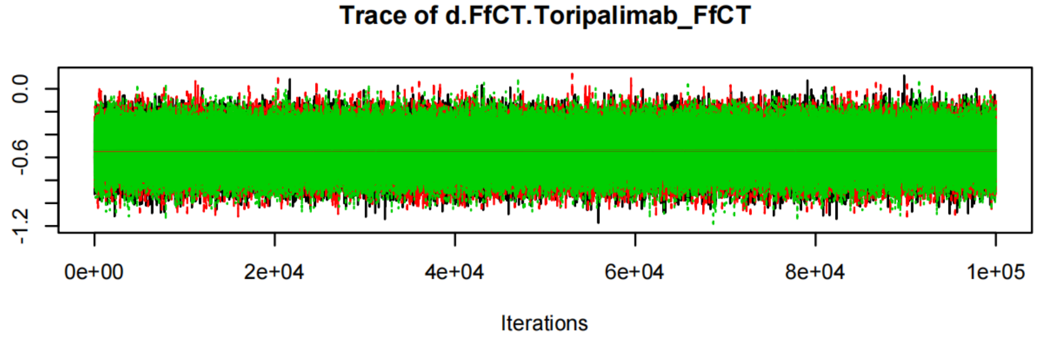
Trace of d.FfCT.Toripalimab_FfCT
0.0
-0.6
-1.2
0e+00
2e+04
4e+04
6e+04
8e+04
1e+05
Iterations
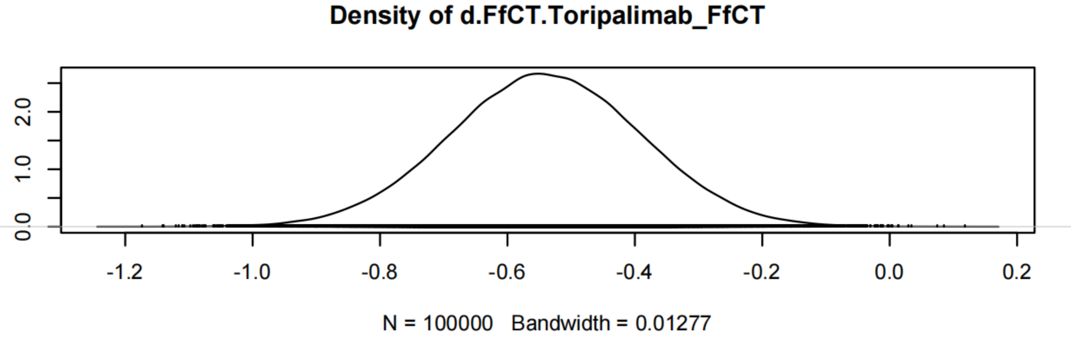
Density of d.FfCT.Toripalimab_FfCT
0.0
1.0
2.0
-1.2
-1.0
-0.8
-0.6
-0.4
-0.2
0.0
0.2
N = 100000 Bandwidth = 0.01277

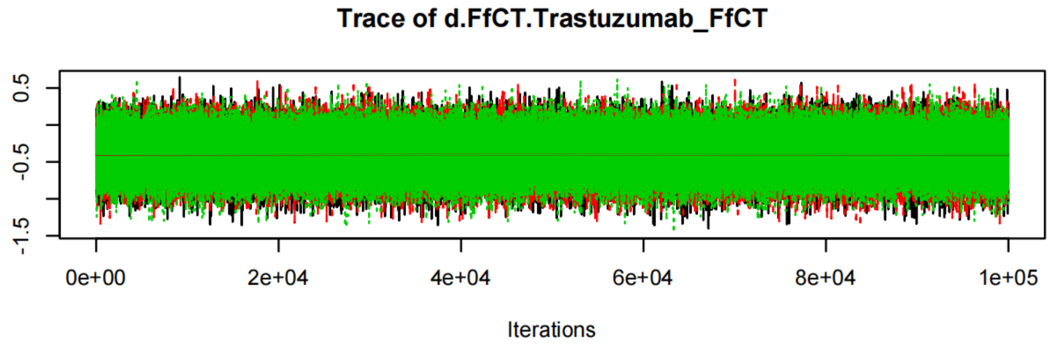

Trace of d.FfCT.Trastuzumab_FfCT
0.5
-0.5
-1.5
0e+00
2e+04
4e+04
6e+04
8e+04
1e+05
Iterations

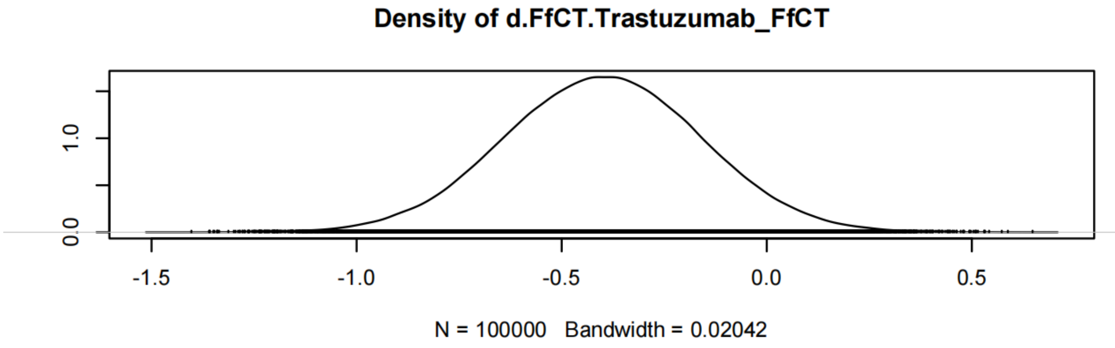

Density of d.FfCT.Trastuzumab_FfCT
1.0
0.0
-1.5
-1.0
-0.5
0.0
0.5
N = 100000 Bandwidth = 0.02042

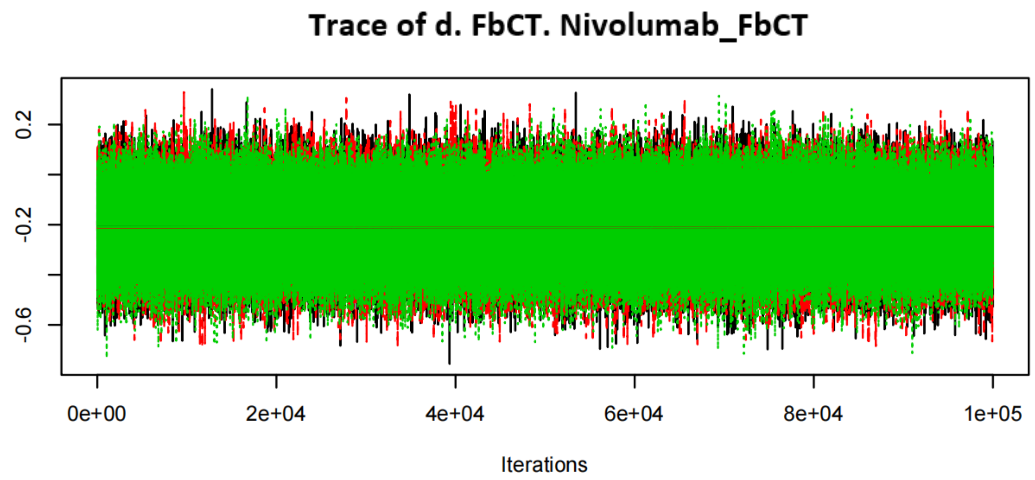
Trace of d. FbCT. Nivolumab_FbCT
0.2
-0.2
-0.6
0e+00
2e+04
4e+04
6e+04
8e+04
1e+05
Iterations

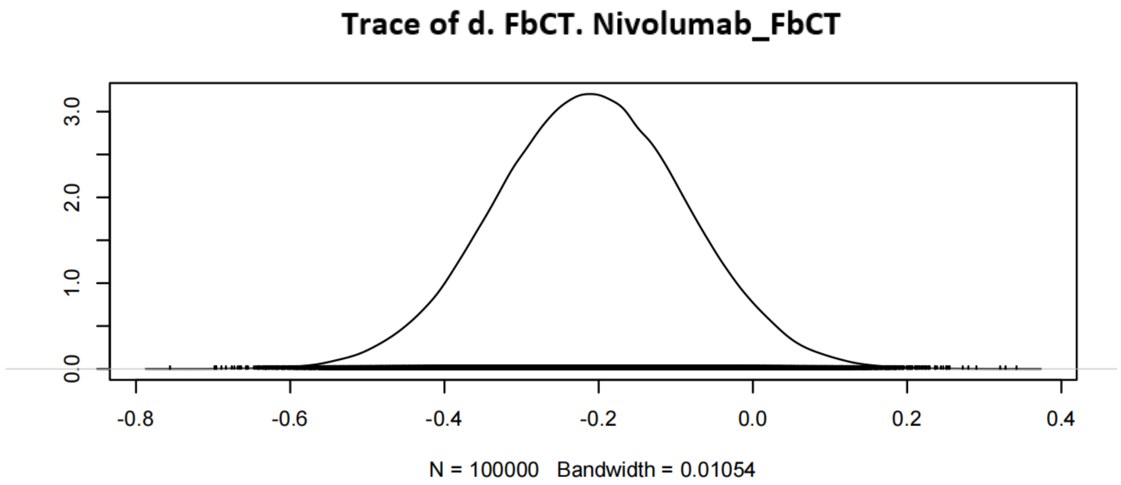
Trace of d. FbCT. Nivolumab_FbCT
3.0
2.0
1.0
0.0
-0.8
-0.6
-0.4
-0.2
0.0
0.2
0.4
N = 100000 Bandwidth = 0.01054

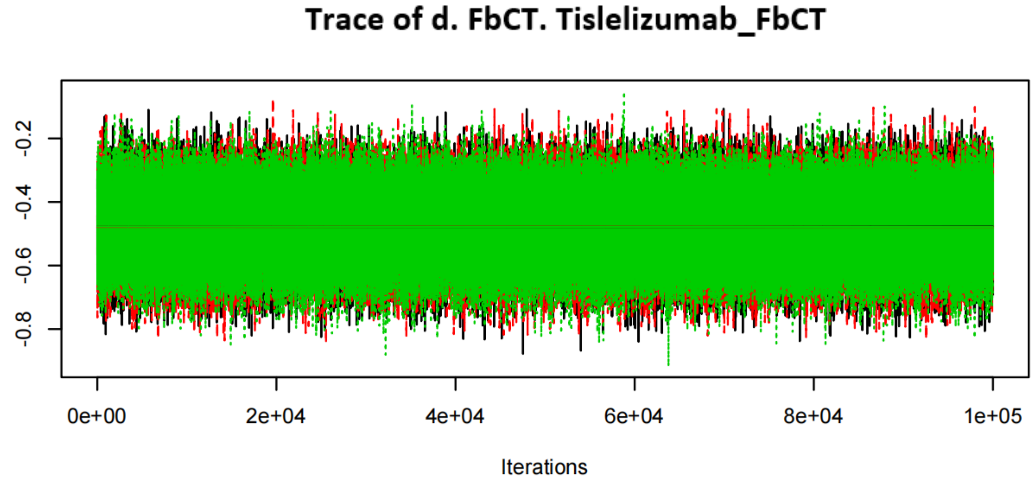
Trace of d. FbCT. Tislelizumab_FbCT
-0.2
-0.4
-0.6
-0.8
0e+00
2e+04
4e+04
6e+04
8e+04
1e+05
Iterations

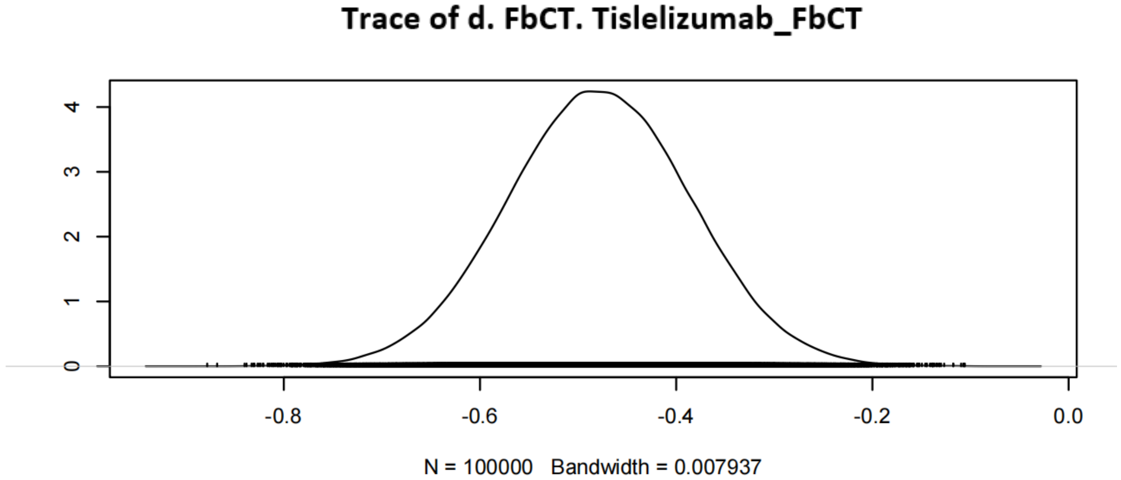
Trace of d. FbCT. Tislelizumab_FbCT
4
3
2
1
0
-0.8
-0.6
-0.4
-0.2
0.0
N = 100000 Bandwidth = 0.007937

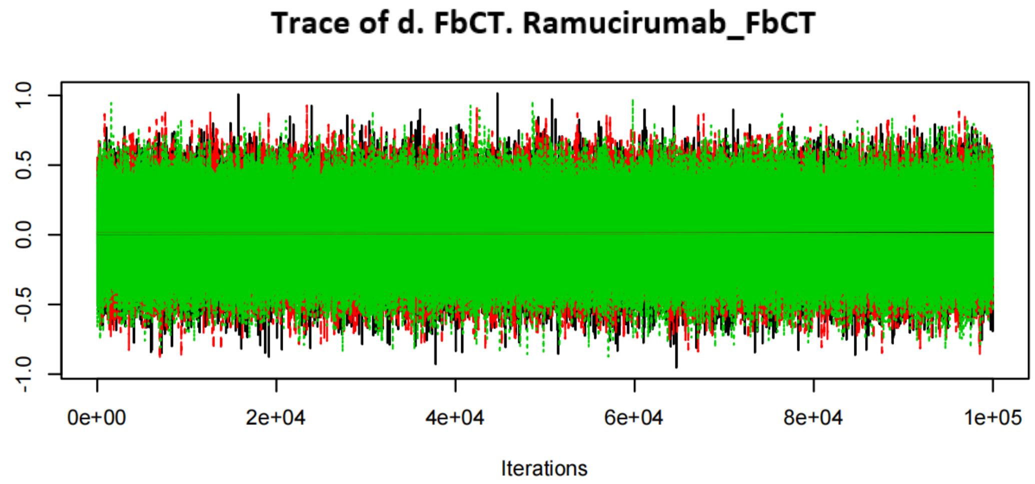
Trace of d. FbCT. Ramucirumab_FbCT
1.0
0.5
0.0
-0.5
-1.0
0e+00
2e+04
4e+04
6e+04
8e+04
1e+05
Iterations

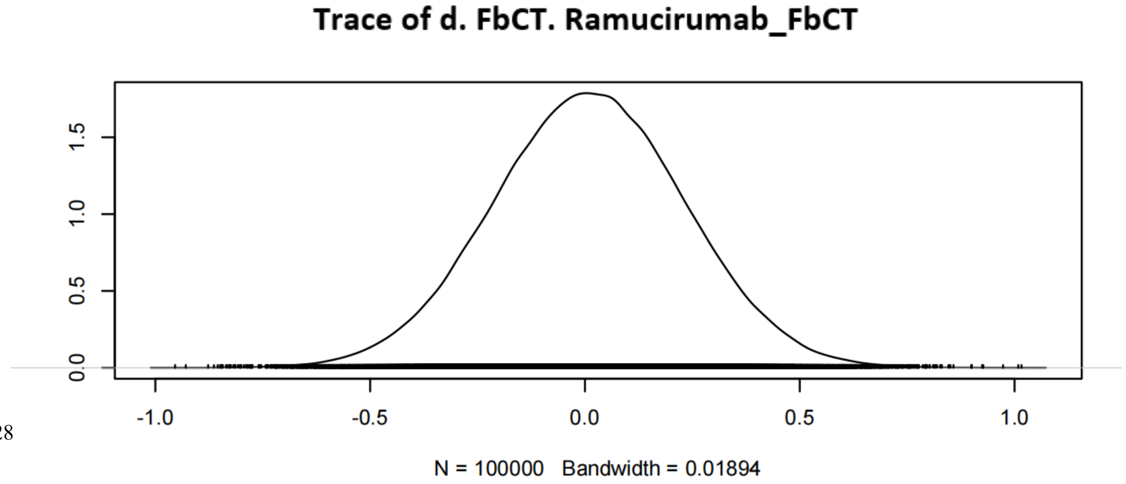
Trace of d. FbCT. Ramucirumab_FbCT
1.5
1.0
0.5
0.0
-1.0
-0.5
0.0
0.5
1.0
N = 100000 Bandwidth = 0.01894

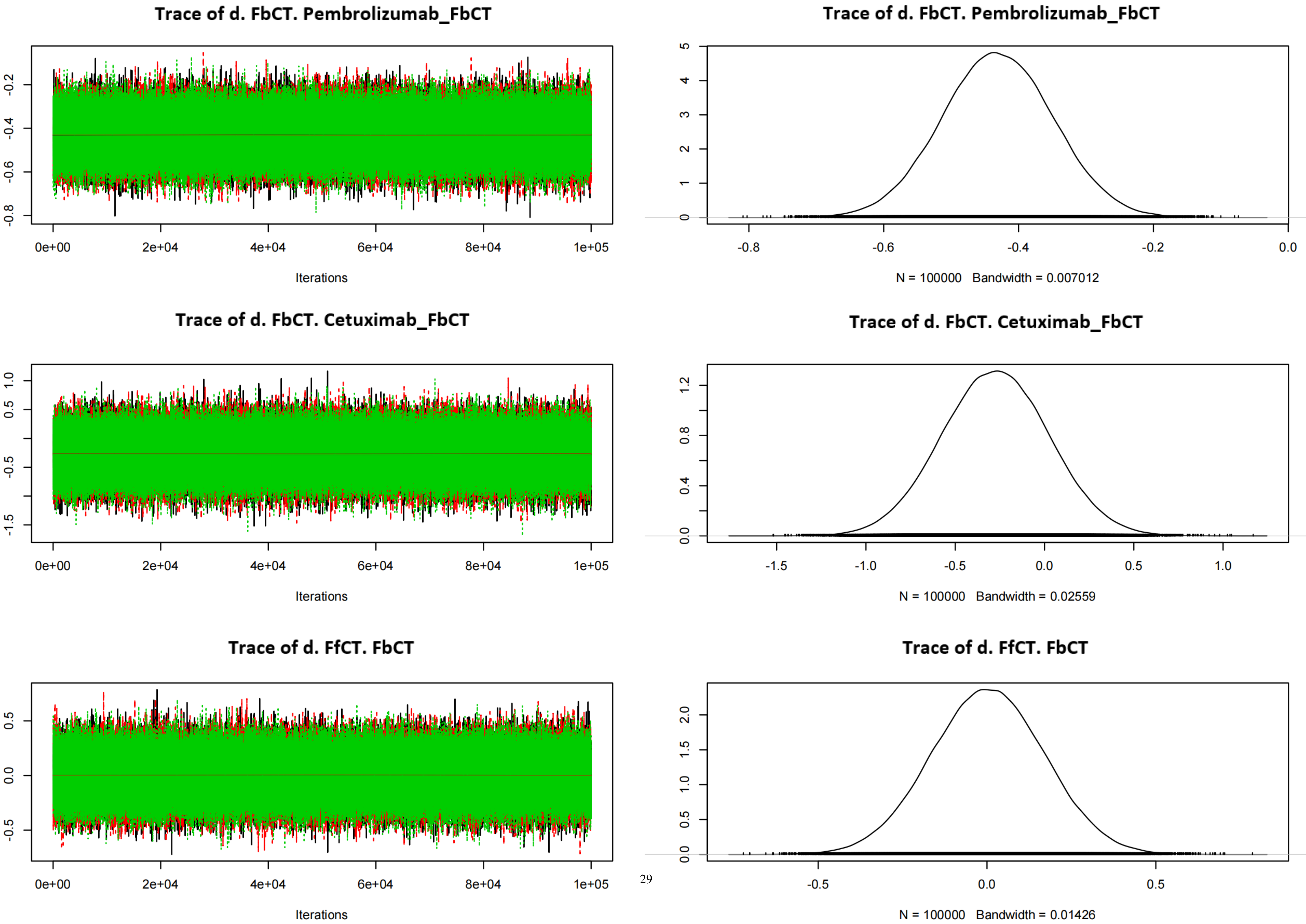

Trace of d. FbCT. Pembrolizumab_FbCT
Iterations
Trace of d. FbCT. Pembrolizumab_FbCT
N = 100000 Bandwidth = 0.007012
Trace of d. FbCT. Cetuximab_FbCT
Iterations
Trace of d. FbCT. Cetuximab_FbCT
N = 100000 Bandwidth = 0.02559
Trace of d. FfCT. FbCT
Iterations
Trace of d. FfCT. FbCT
N = 100000 Bandwidth = 0.01426

Trace of d. FfCT. Rilotumumab_FfCT

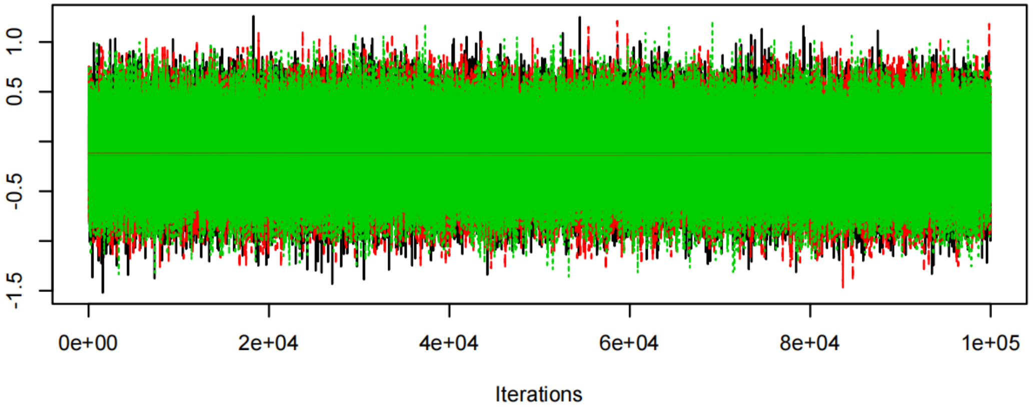

Trace of d. FfCT. Rilotumumab_FfCT

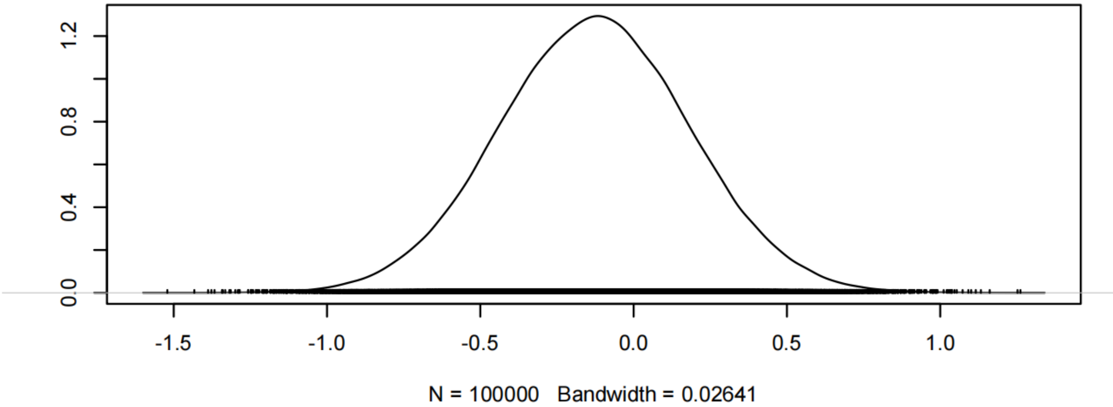

Trace of d. FfCT. Camrelizumab_FfCT

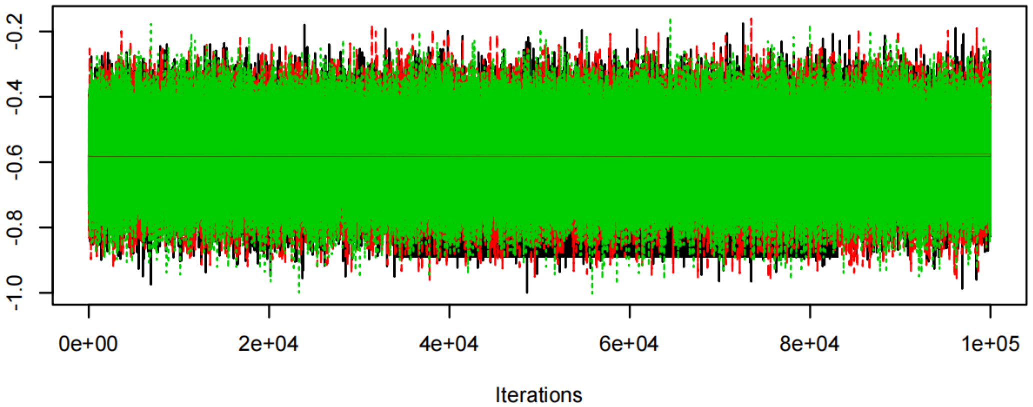

Trace of d. FfCT. Camrelizumab_FfCT

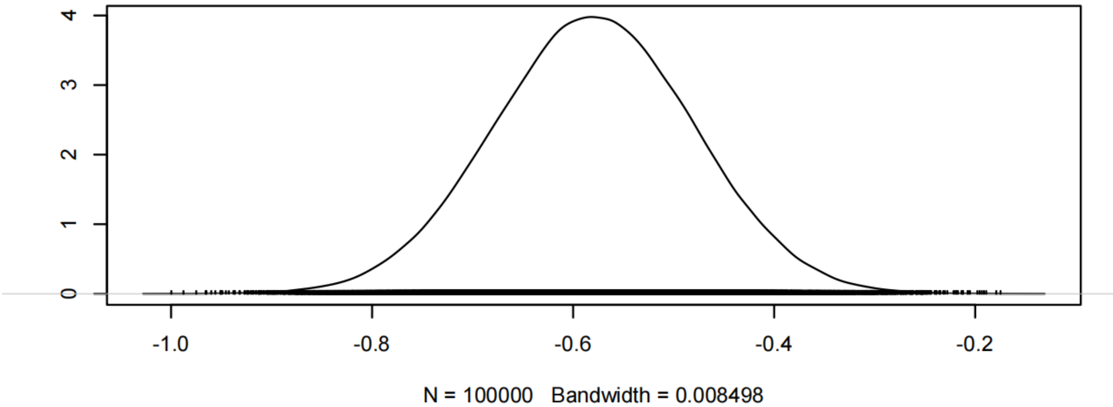

Trace of d. FfCT. Toripalimab_FfCT

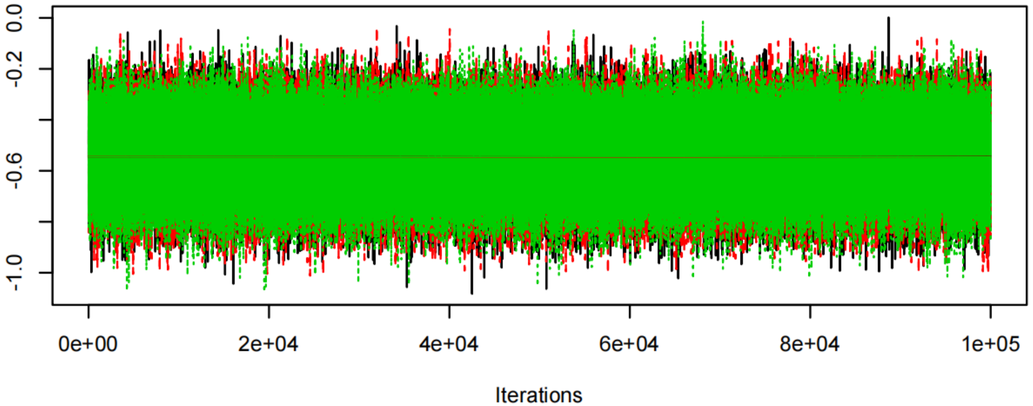

Trace of d. FfCT. Toripalimab_FfCT

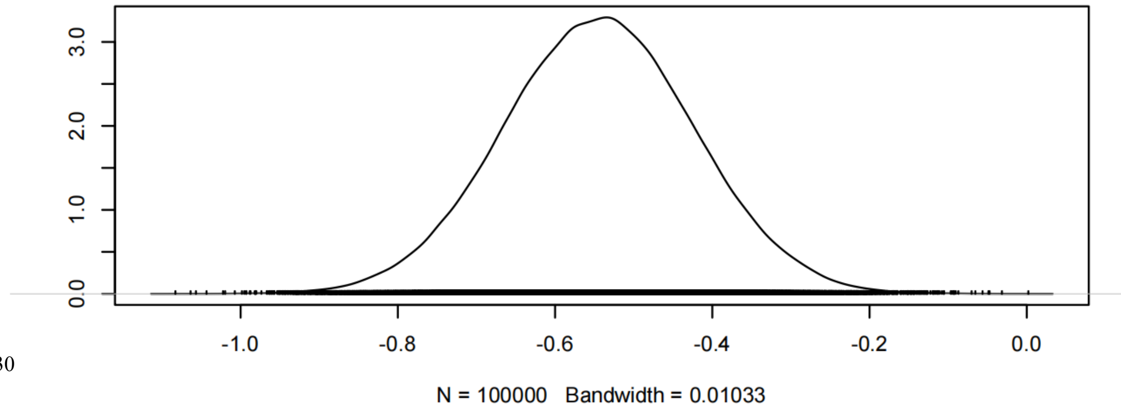

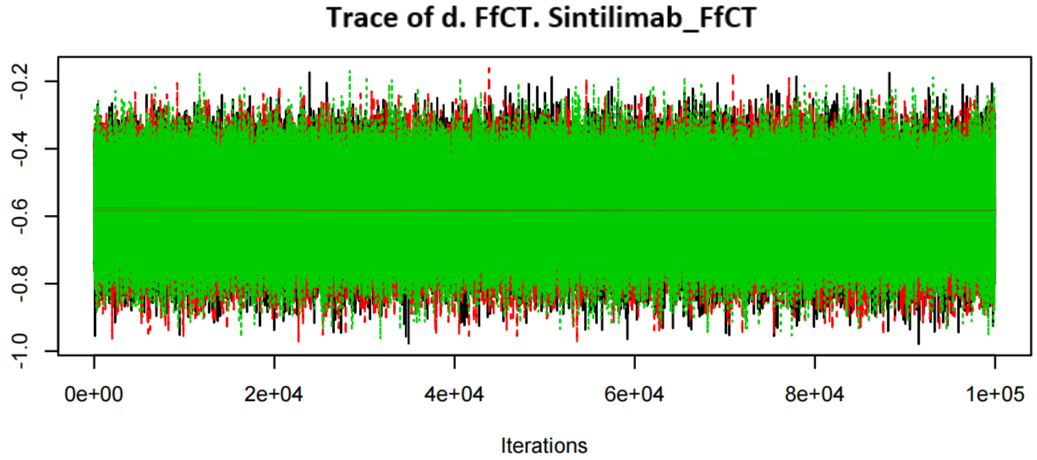
Trace of d. FfCT. Sintilimab_FfCT
Iterations

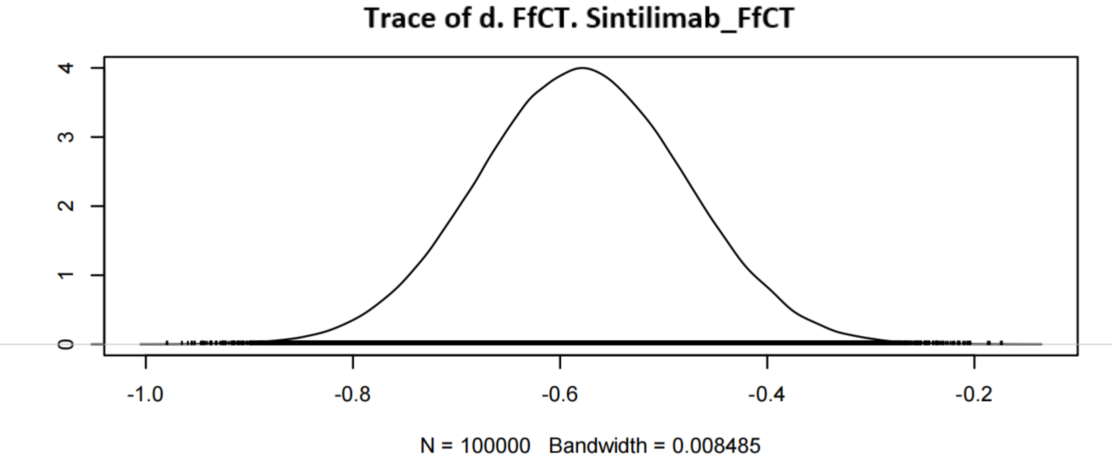
Trace of d. FfCT. Sintilimab_FfCT
N = 100000 Bandwidth = 0.008485

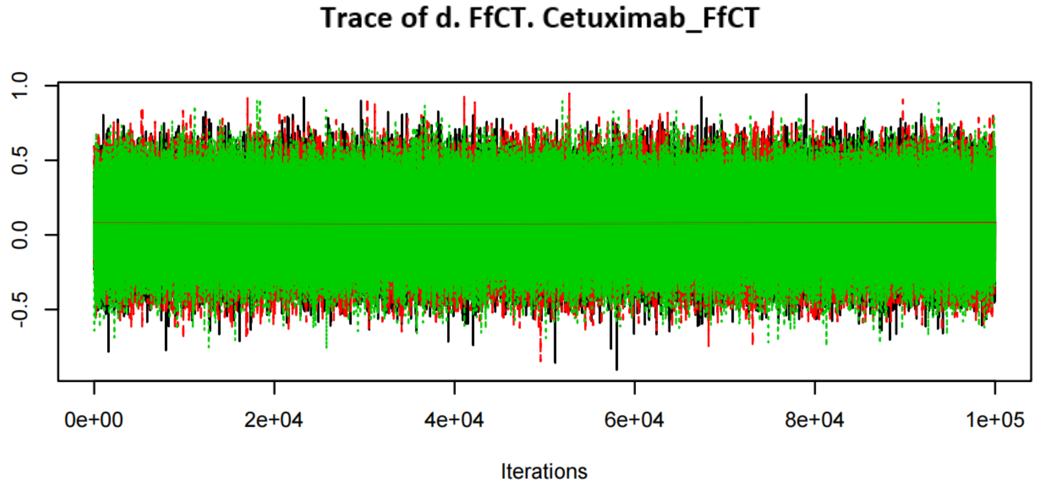
Trace of d. FfCT. Cetuximab_FfCT
Iterations

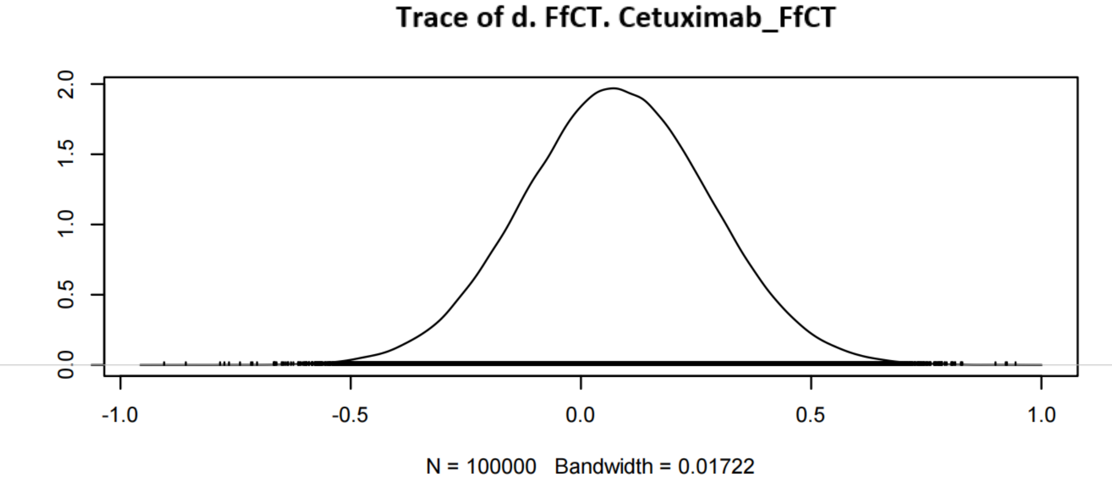
Trace of d. FfCT. Cetuximab_FfCT
N = 100000 Bandwidth = 0.01722

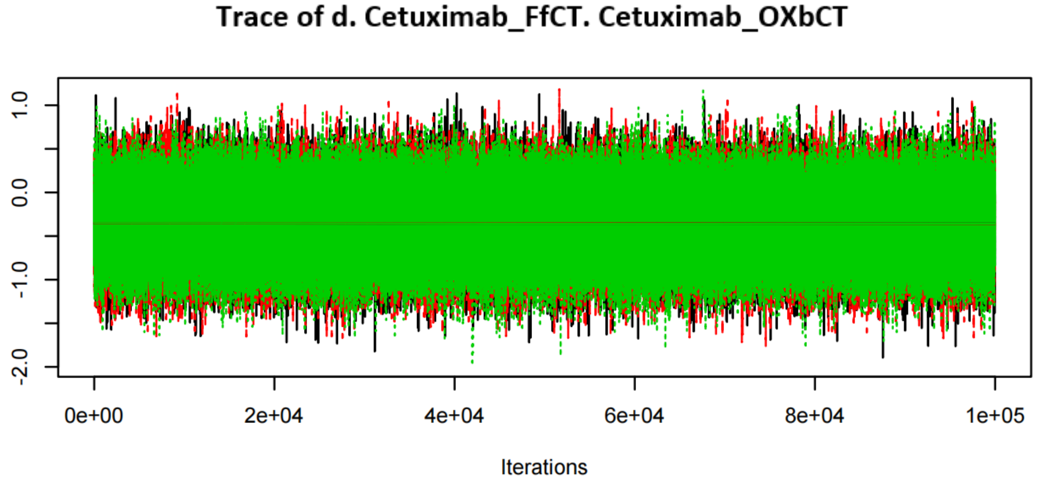
Trace of d. Cetuximab_FfCT. Cetuximab_OXbCT
Iterations

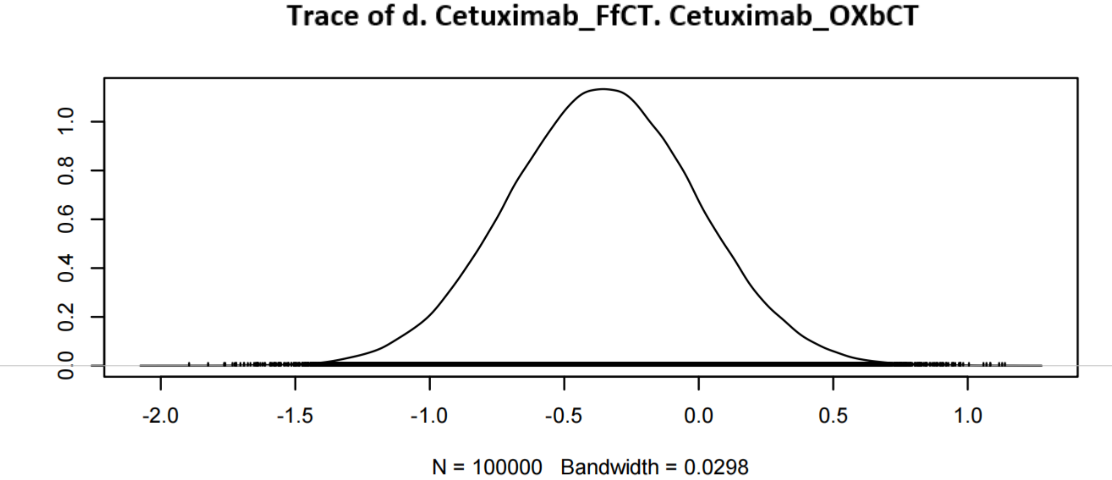
Trace of d. Cetuximab_FfCT. Cetuximab_OXbCT
N = 100000 Bandwidth = 0.0298

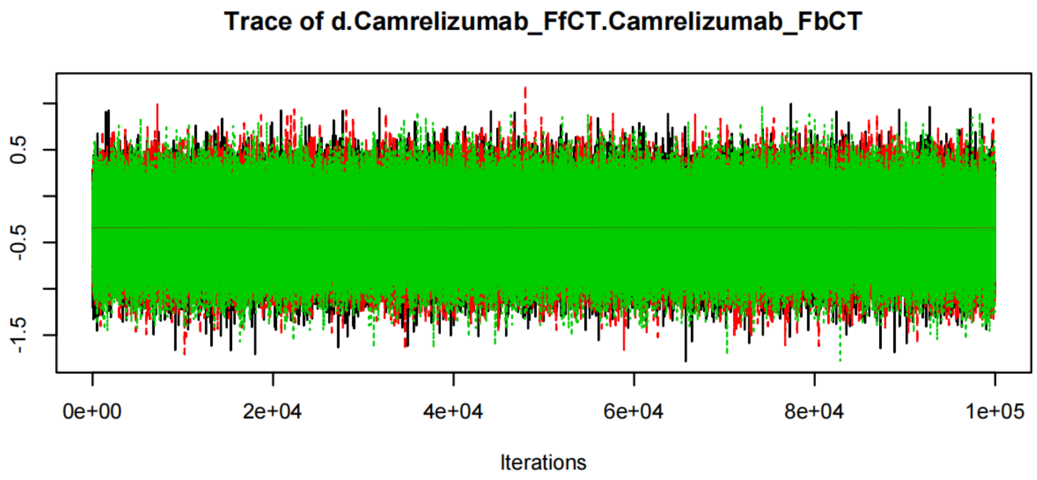
Trace of d.Camrelizumab_FfCT.Camrelizumab_FbCT
0.5
-0.5
-1.5
0e+00
2e+04
4e+04
6e+04
8e+04
1e+05
Iterations

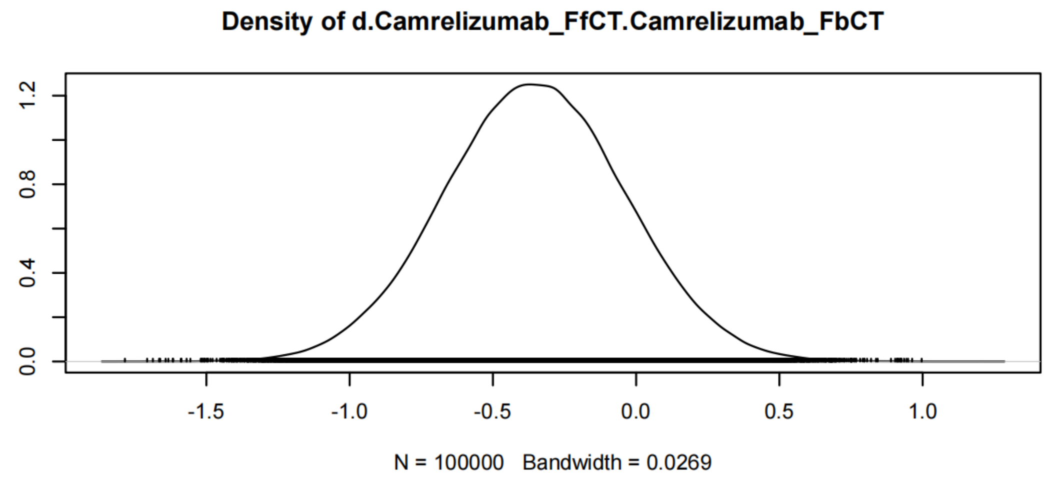
Density of d.Camrelizumab_FfCT.Camrelizumab_FbCT
1.2
0.8
0.4
0.0
-1.5
-1.0
-0.5
0.0
0.5
1.0
N = 100000 Bandwidth = 0.0269

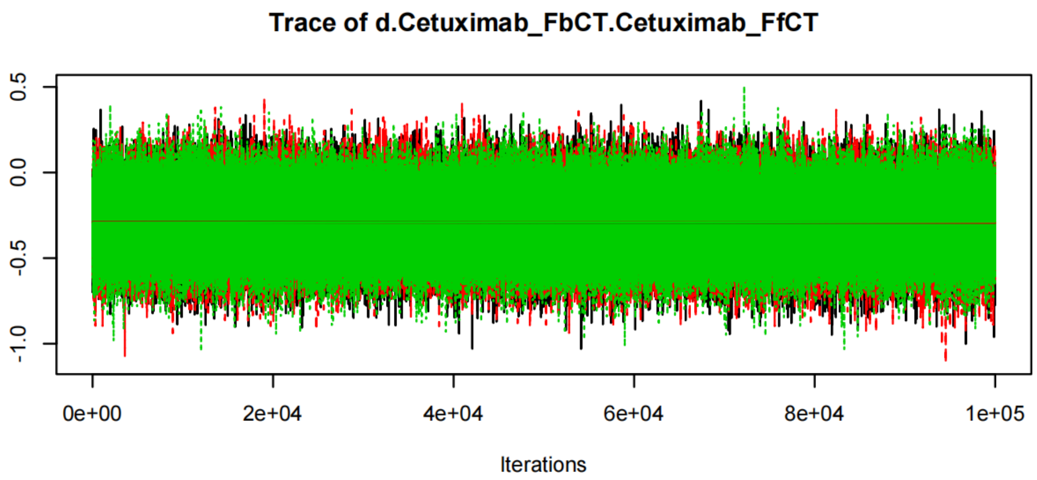
Trace of d.Cetuximab_FbCT.Cetuximab_FfCT
0.5
0.0
-0.5
-1.0
0e+00
2e+04
4e+04
6e+04
8e+04
1e+05
Iterations

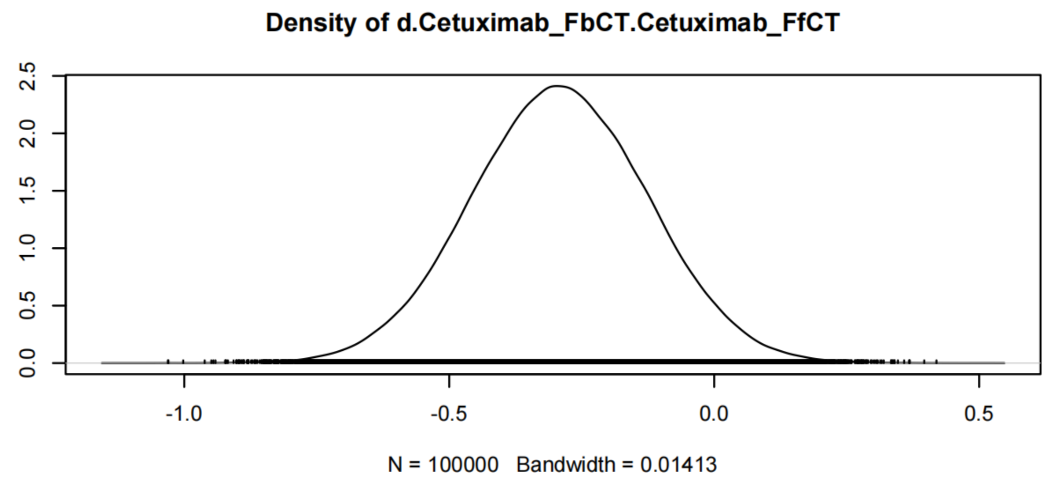
Density of d.Cetuximab_FbCT.Cetuximab_FfCT
2.5
2.0
1.5
1.0
0.5
0.0
-1.0
-0.5
0.0
0.5
N = 100000 Bandwidth = 0.01413

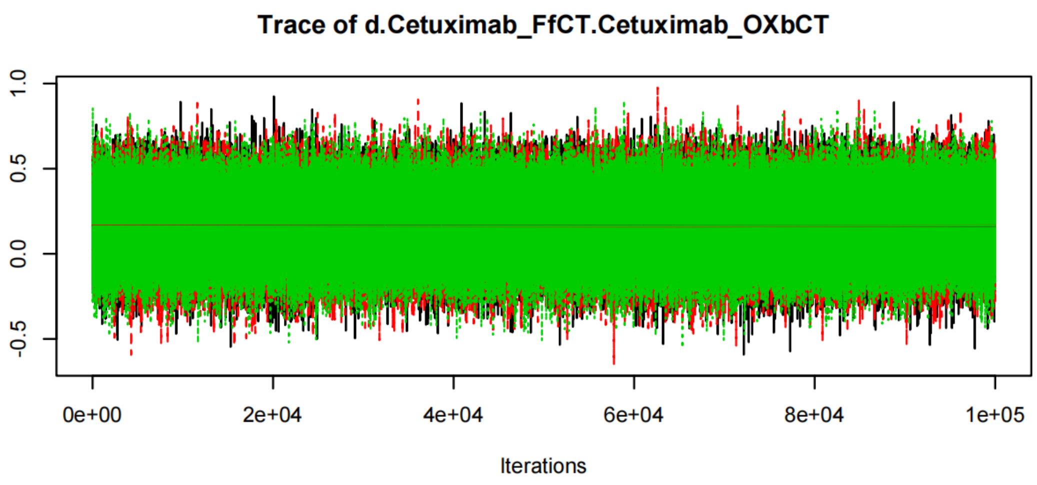
Trace of d.Cetuximab_FfCT.Cetuximab_OXbCT
1.0
0.5
0.0
-0.5
0e+00
2e+04
4e+04
6e+04
8e+04
1e+05
Iterations

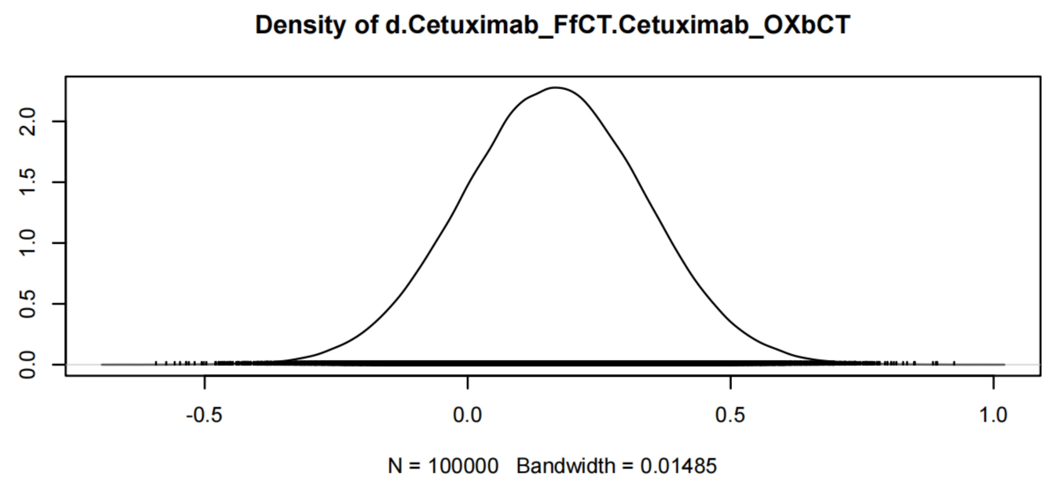
Density of d.Cetuximab_FfCT.Cetuximab_OXbCT
2.0
1.5
1.0
0.5
0.0
-0.5
0.0
0.5
1.0
N = 100000 Bandwidth = 0.01485

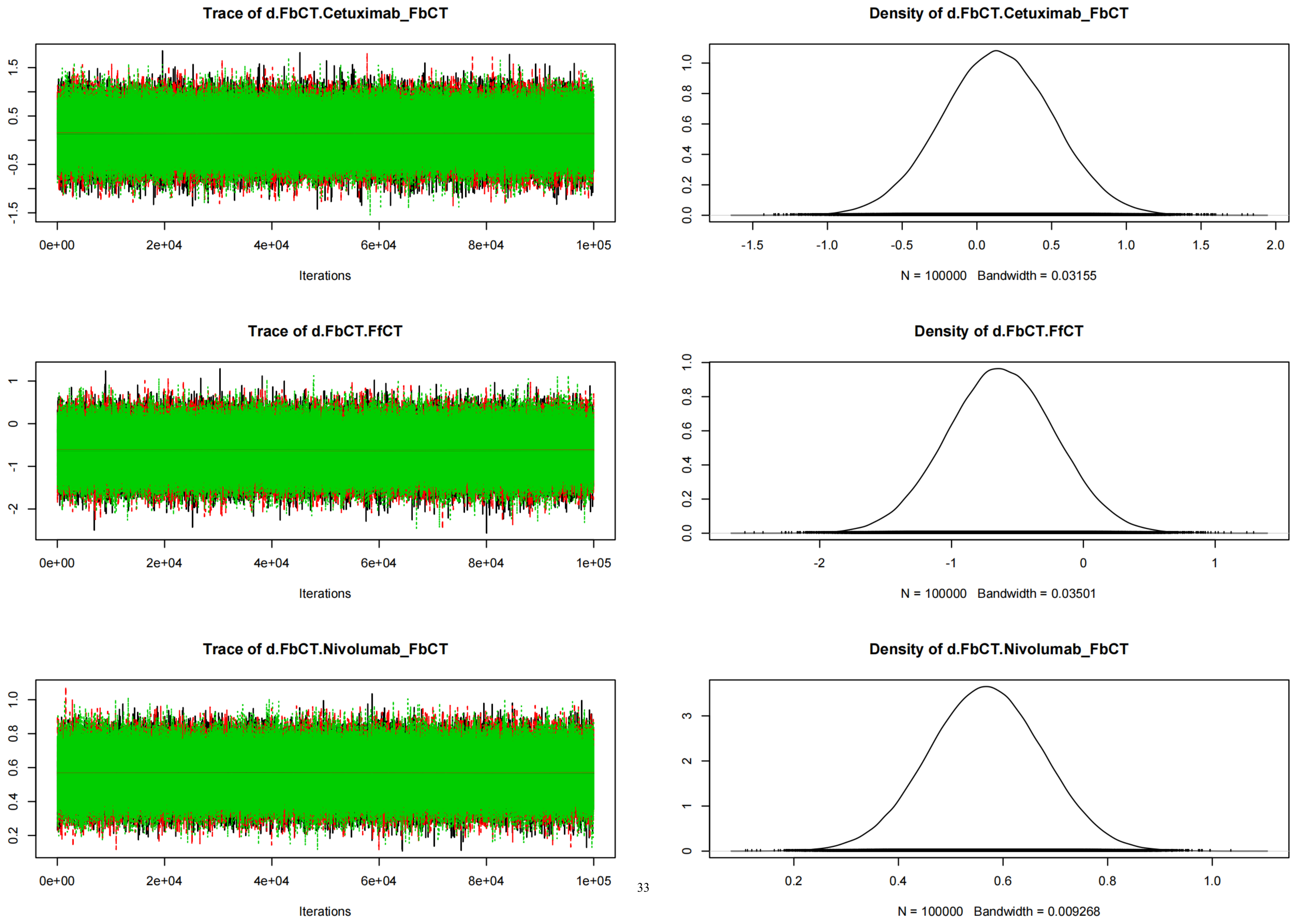
Trace of d.FbCT.Cetuximab_FbCT
-1.5
-0.5
0.5
1.5
0e+00
2e+04
4e+04
6e+04
8e+04
1e+05
Iterations
Density of d.FbCT.Cetuximab_FbCT
0.0
0.2
0.4
0.6
0.8
1.0
-1.5
-1.0
-0.5
0.0
0.5
1.0
1.5
2.0
N = 100000 Bandwidth = 0.03155
Trace of d.FbCT.FfCT
-2
-1
0
1
0e+00
2e+04
4e+04
6e+04
8e+04
1e+05
Iterations
Density of d.FbCT.FfCT
0.0
0.2
0.4
0.6
0.8
1.0
-2
-1
0
1
N = 100000 Bandwidth = 0.03501
Trace of d.FbCT.Nivolumab_FbCT
0.2
0.4
0.6
0.8
1.0
0e+00
2e+04
4e+04
6e+04
8e+04
1e+05
Iterations
Density of d.FbCT.Nivolumab_FbCT
0
1
2
3
0.2
0.4
0.6
0.8
1.0
N = 100000 Bandwidth = 0.009268

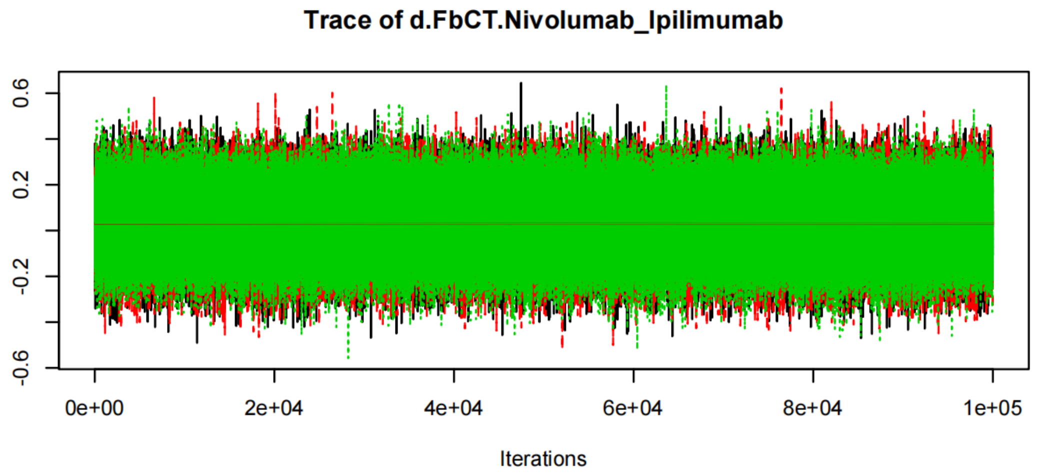
Trace of d.FbCT.Nivolumab_Ipilimumab
Iterations
0e+00
2e+04
4e+04
6e+04
8e+04
1e+05
-0.6
-0.2
0.2
0.6

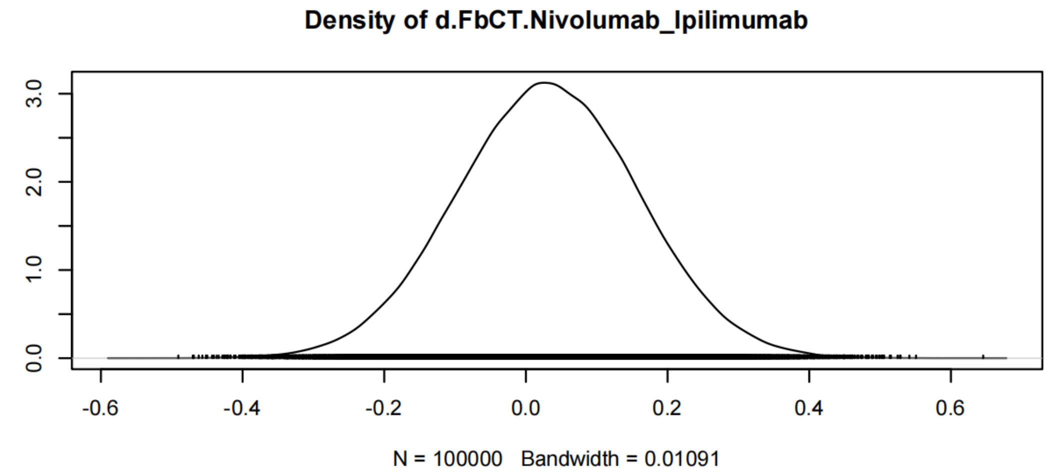
Density of d.FbCT.Nivolumab_Ipilimumab
N = 100000 Bandwidth = 0.01091
-0.6
-0.4
-0.2
0.0
0.2
0.4
0.6
0.0
1.0
2.0
3.0

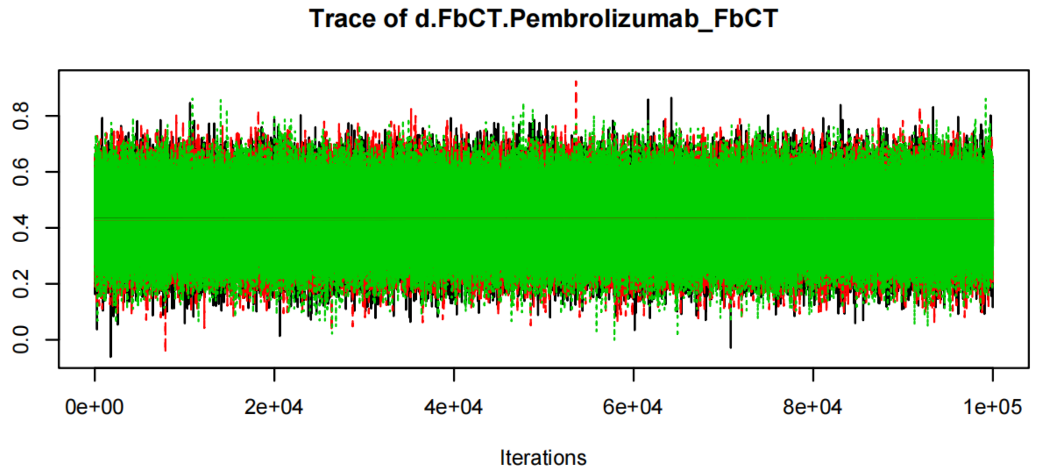
Trace of d.FbCT.Pembrolizumab_FbCT
Iterations
0e+00
2e+04
4e+04
6e+04
8e+04
1e+05
0.0
0.2
0.4
0.6
0.8

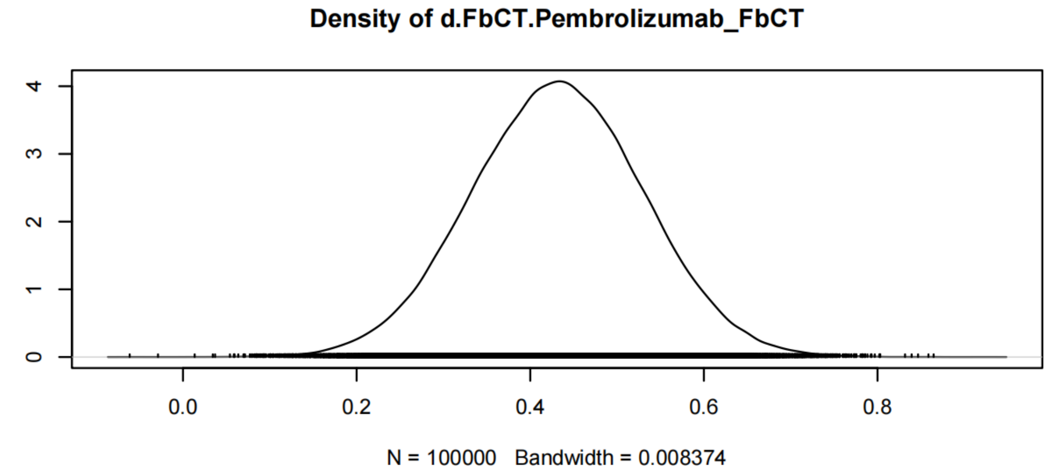
Density of d.FbCT.Pembrolizumab_FbCT
N = 100000 Bandwidth = 0.008374
0.0
0.2
0.4
0.6
0.8
0
1
2
3
4

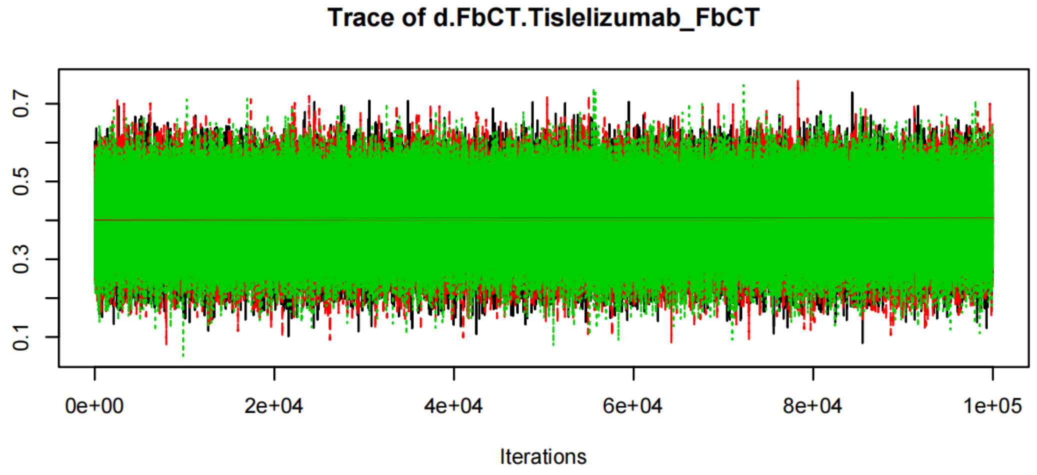
Trace of d.FbCT.Tislelizumab_FbCT
Iterations
0e+00
2e+04
4e+04
6e+04
8e+04
1e+05
0.1
0.3
0.5
0.7

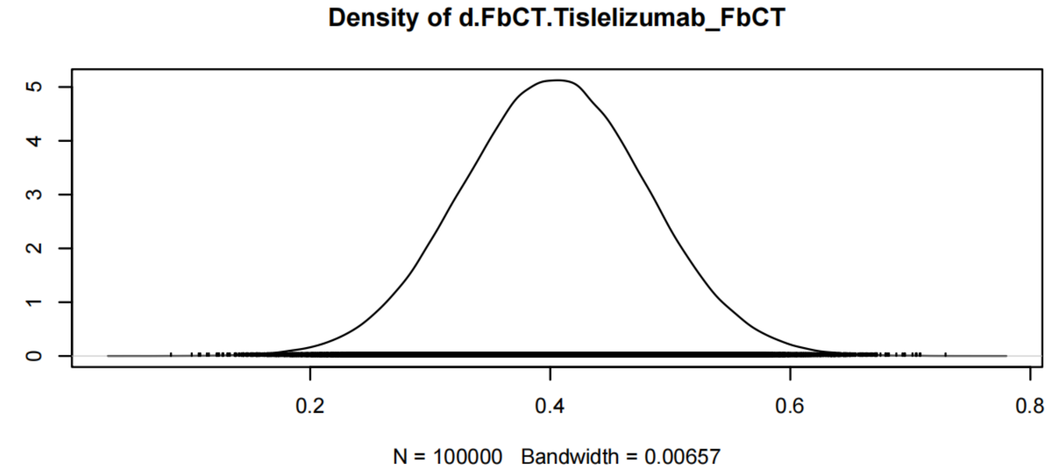
Density of d.FbCT.Tislelizumab_FbCT
N = 100000 Bandwidth = 0.00657
0.2
0.4
0.6
0.8
0
1
2
3
4
5

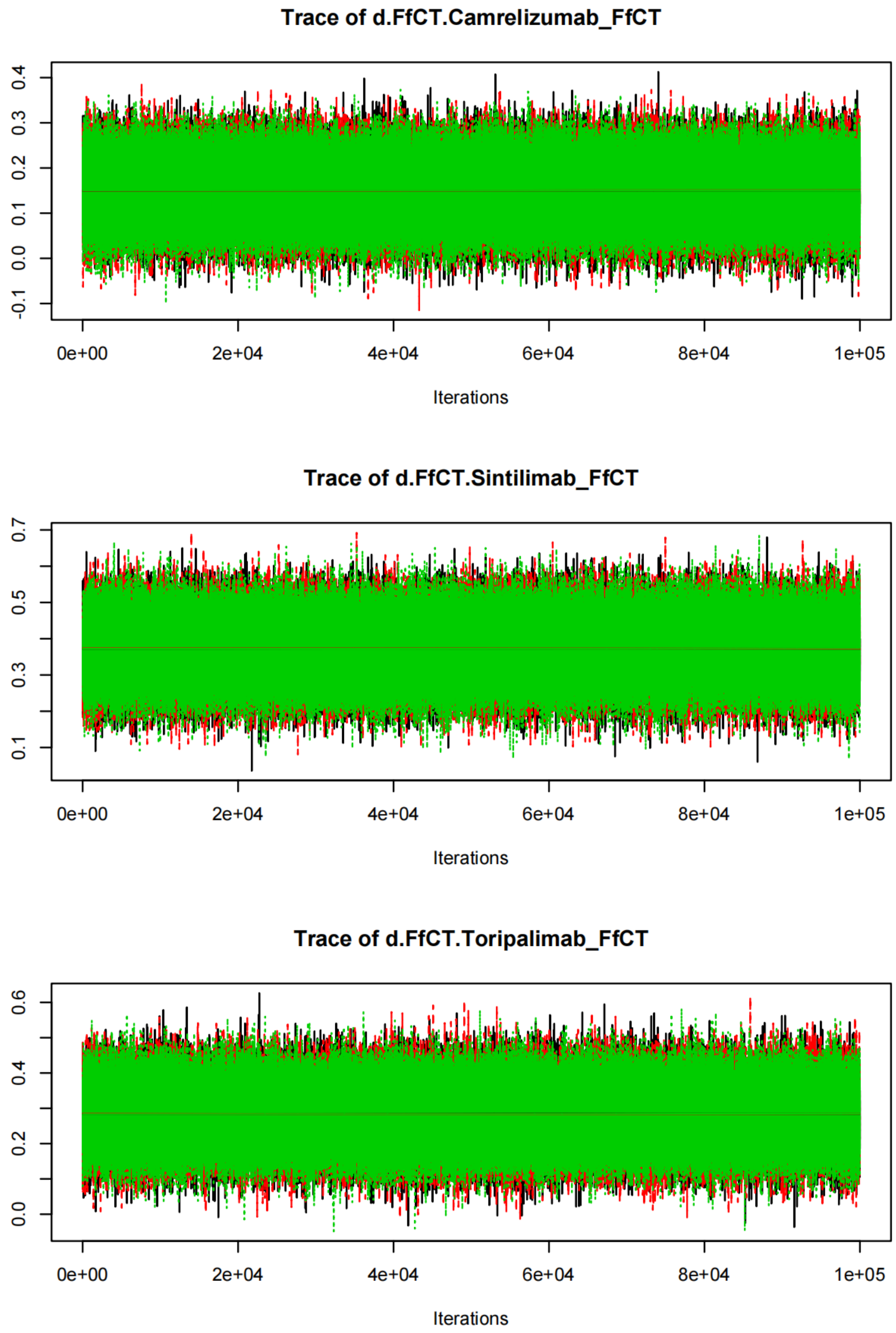
Trace of d.FfCT.Camrelizumab_FfCT
Iterations
Trace of d.FfCT.Sintilimab_FfCT
Iterations
Trace of d.FfCT.Toripalimab_FfCT
Iterations

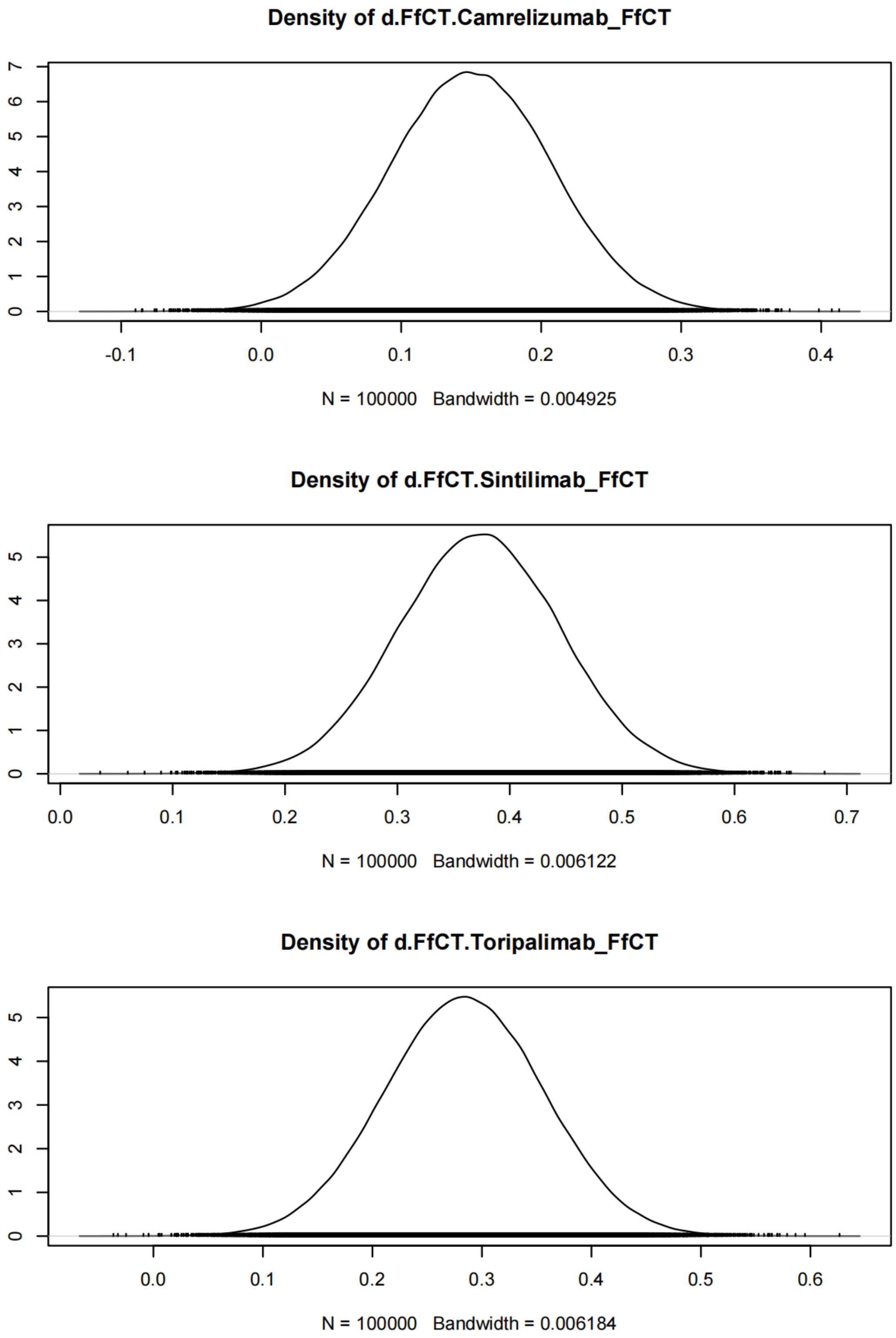
Density of d.FfCT.Camrelizumab_FfCT
N = 100000 Bandwidth = 0.004925
Density of d.FfCT.Sintilimab_FfCT
N = 100000 Bandwidth = 0.006122
Density of d.FfCT.Toripalimab_FfCT
N = 100000 Bandwidth = 0.006184

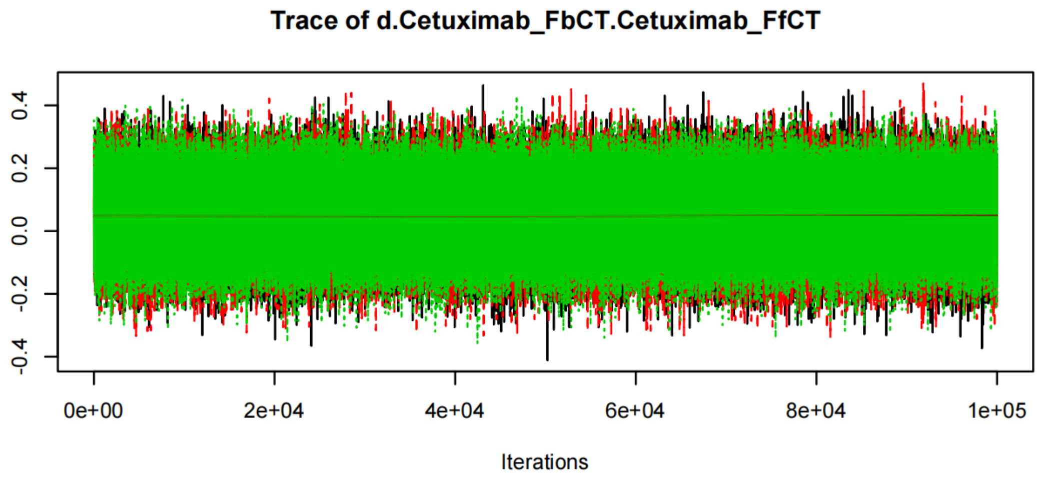
Trace of d.Cetuximab_FbCT.Cetuximab_FfCT
-0.4
-0.2
0.0
0.2
0.4
0e+00
2e+04
4e+04
6e+04
8e+04
1e+05
Iterations

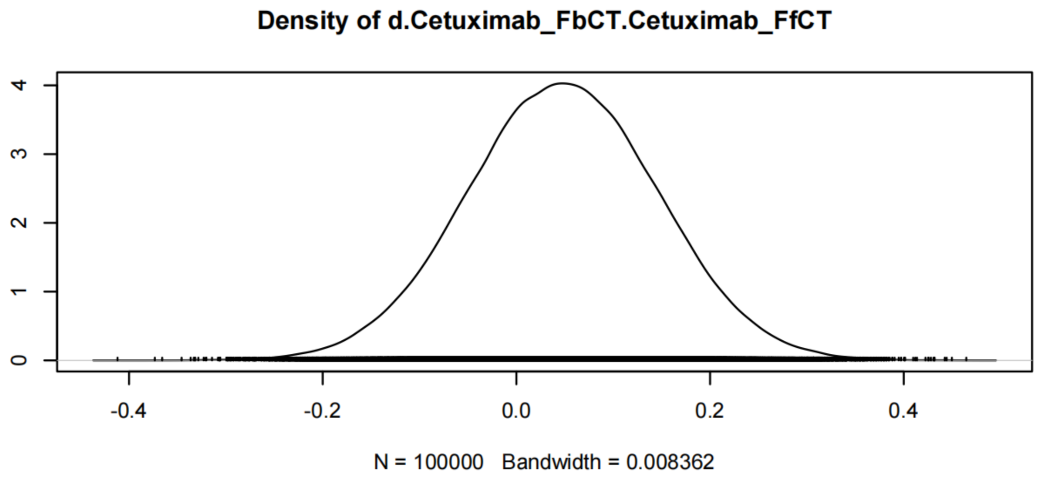
Density of d.Cetuximab_FbCT.Cetuximab_FfCT
0
1
2
3
4
-0.4
-0.2
0.0
0.2
0.4
N = 100000 Bandwidth = 0.008362

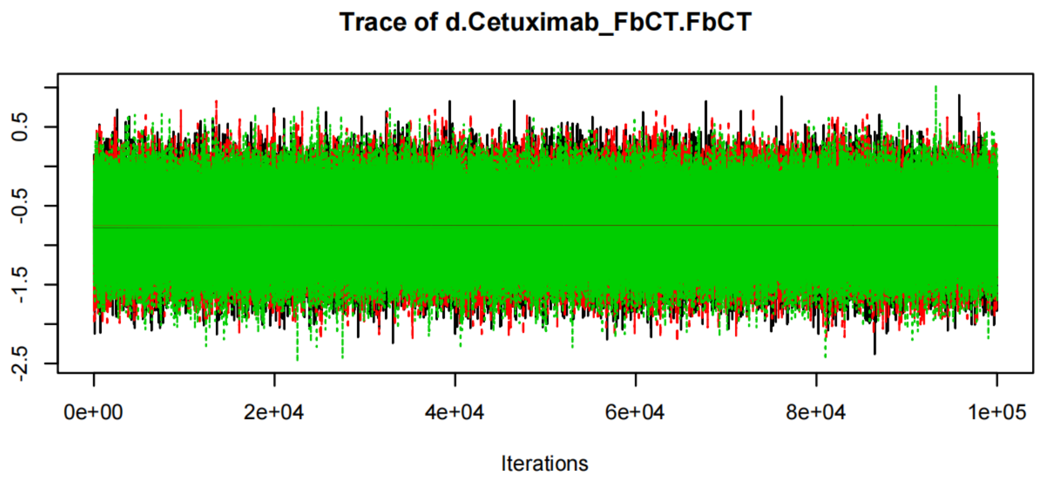
Trace of d.Cetuximab_FbCT.FbCT
-2.5
-1.5
-0.5
0.5
0e+00
2e+04
4e+04
6e+04
8e+04
1e+05
Iterations

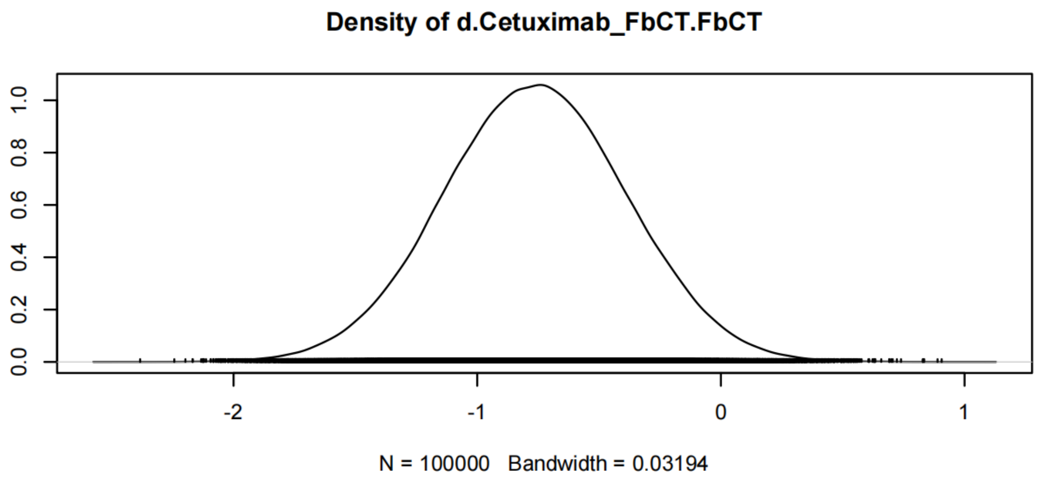
Density of d.Cetuximab_FbCT.FbCT
0.0
0.2
0.4
0.6
0.8
1.0
-2
-1
0
1
N = 100000 Bandwidth = 0.03194

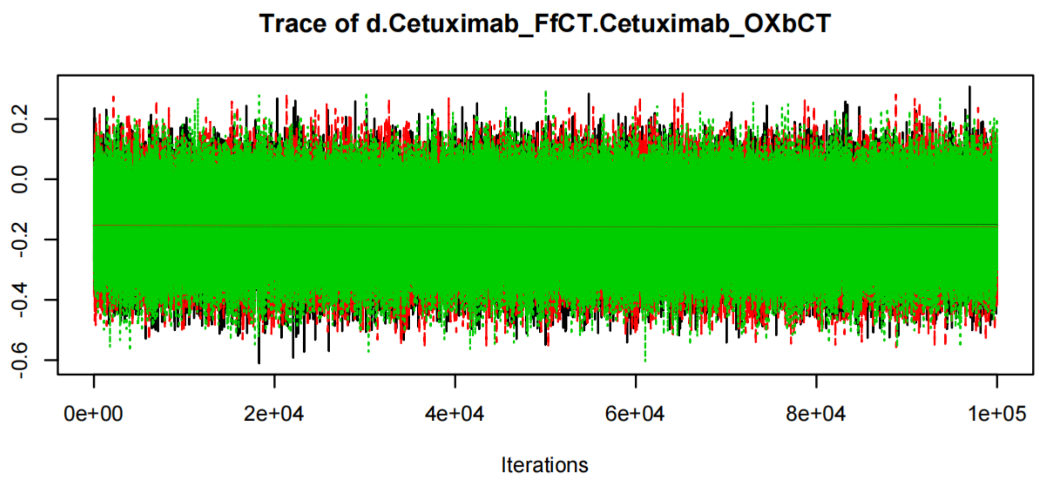
Trace of d.Cetuximab_FfCT.Cetuximab_OXbCT
-0.6
-0.4
-0.2
0.0
0.2
0e+00
2e+04
4e+04
6e+04
8e+04
1e+05
Iterations

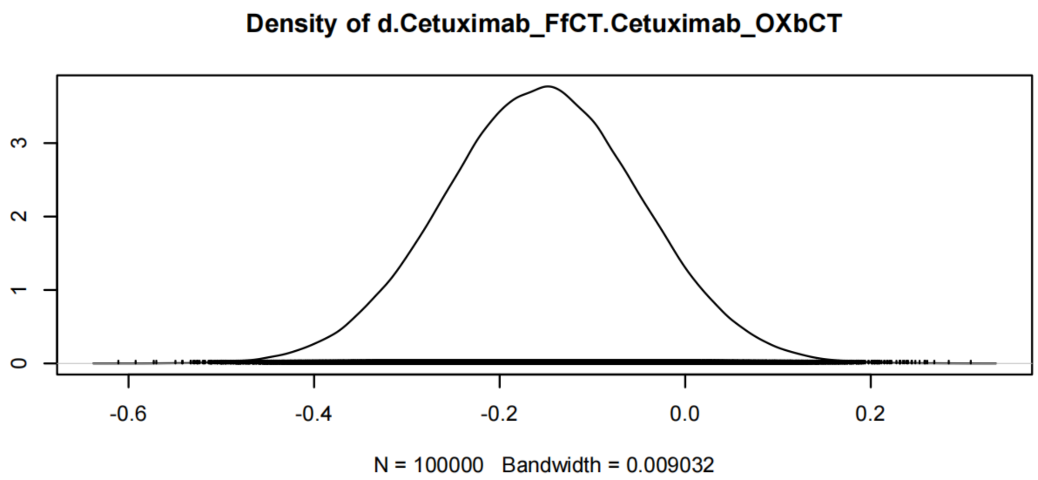
Density of d.Cetuximab_FfCT.Cetuximab_OXbCT
0
1
2
3
-0.6
-0.4
-0.2
0.0
0.2
N = 100000 Bandwidth = 0.009032

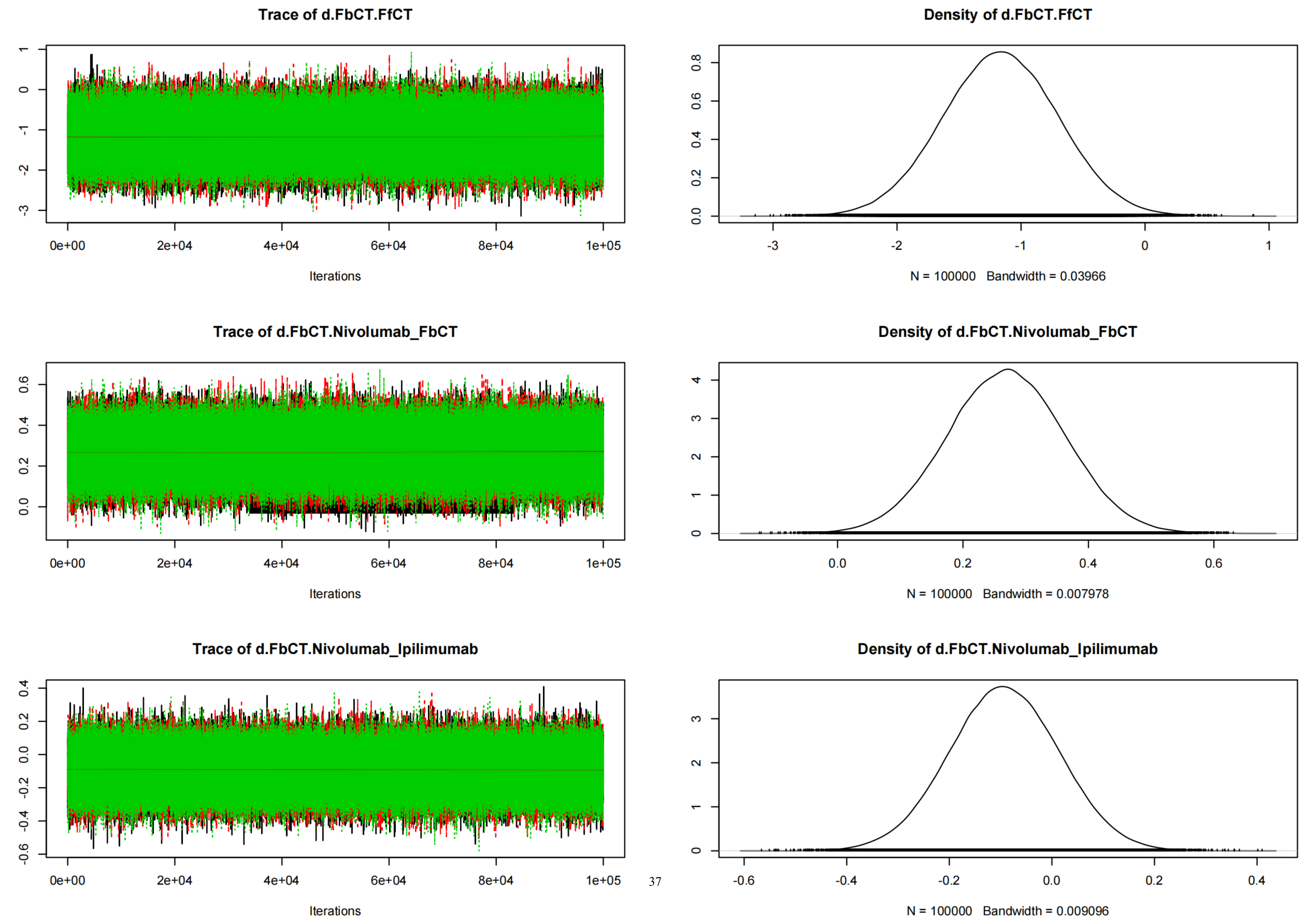
Trace of d.FbCT.FfCT
Iterations
N = 100000 Bandwidth = 0.03966
Density of d.FbCT.FfCT
Trace of d.FbCT.Nivolumab_FbCT
Iterations
Density of d.FbCT.Nivolumab_FbCT
N = 100000 Bandwidth = 0.007978
Trace of d.FbCT.Nivolumab_Ipilimumab
Iterations
Density of d.FbCT.Nivolumab_Ipilimumab
N = 100000 Bandwidth = 0.009096

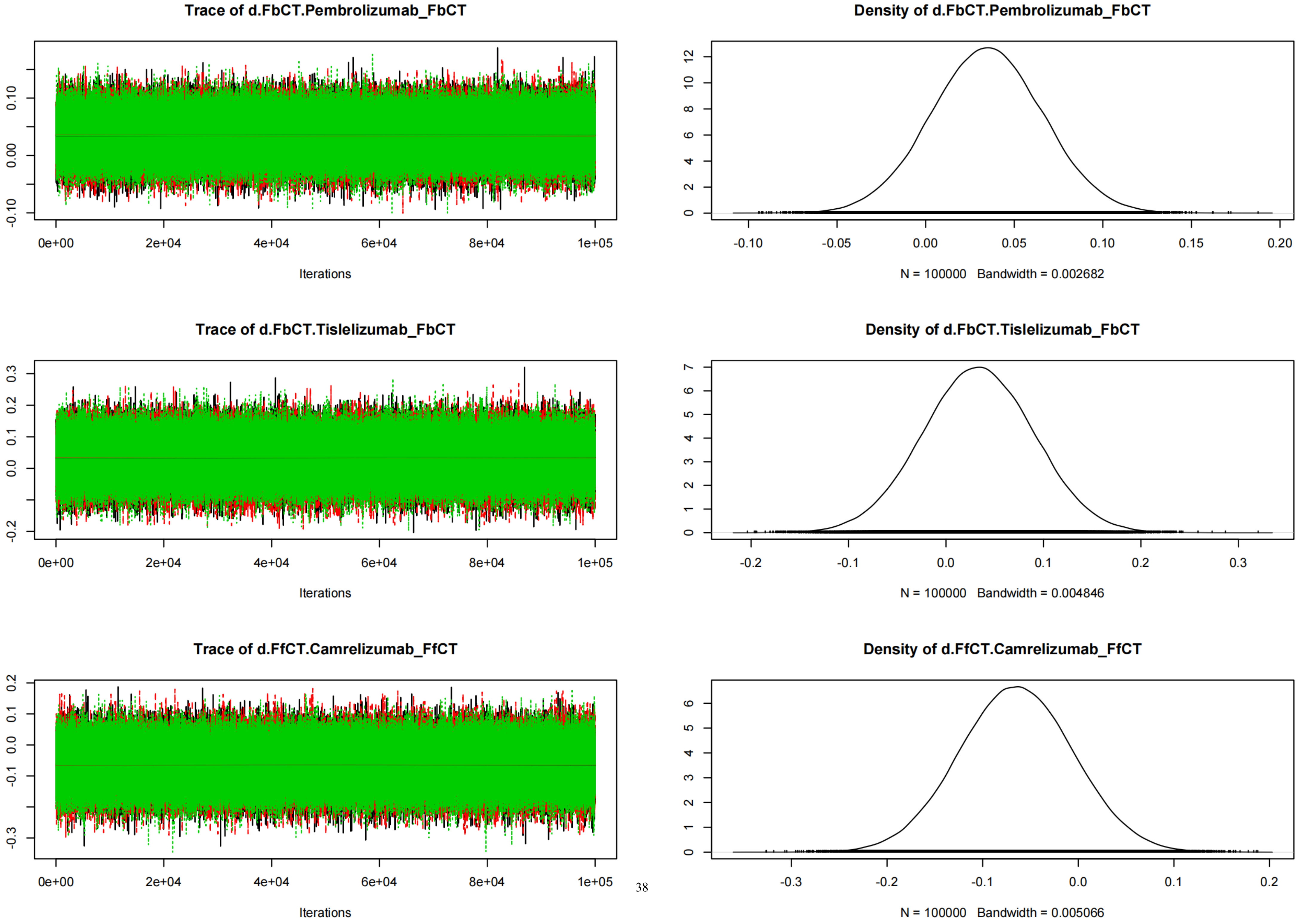
Trace of d.FbCT.Pembrolizumab_FbCT
Iterations
Density of d.FbCT.Pembrolizumab_FbCT
N = 100000 Bandwidth = 0.002682
Trace of d.FbCT.Tislelizumab_FbCT
Iterations
Density of d.FbCT.Tislelizumab_FbCT
N = 100000 Bandwidth = 0.004846
Trace of d.FfCT.Camrelizumab_FfCT
Iterations
Density of d.FfCT.Camrelizumab_FfCT
N = 100000 Bandwidth = 0.005066

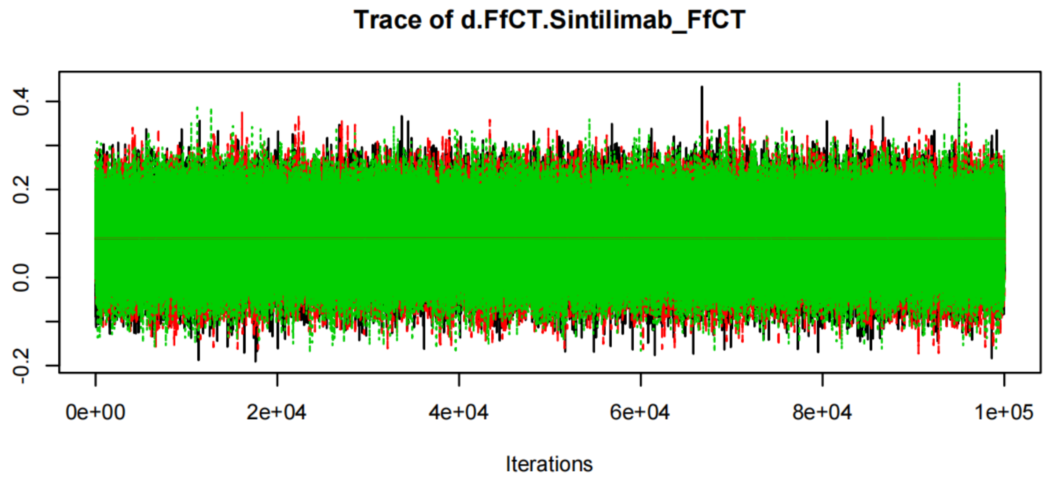
Trace of d.FfCT.Sintilimab_FfCT
-0.2
0.0
0.2
0.4
0e+00
2e+04
4e+04
6e+04
8e+04
1e+05
Iterations

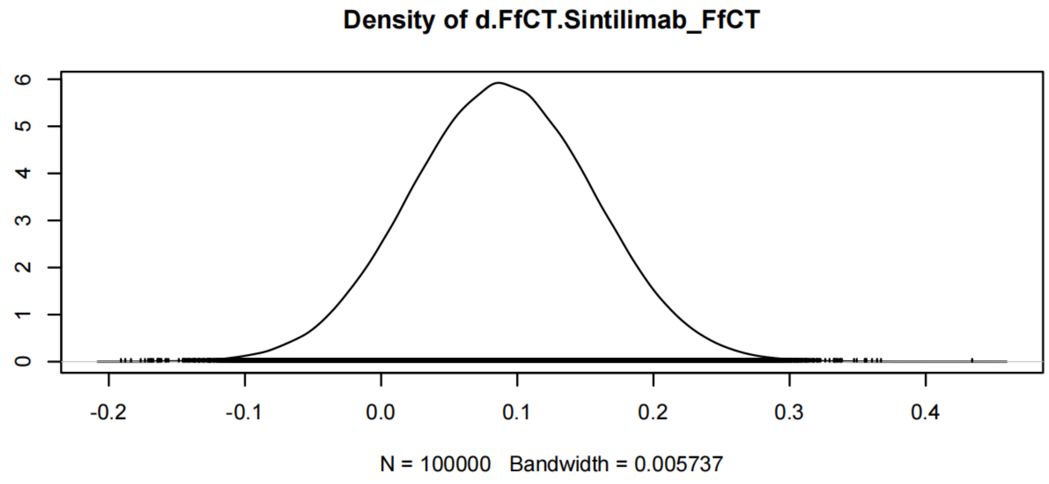
Density of d.FfCT.Sintilimab_FfCT
0
1
2
3
4
5
6
-0.2
-0.1
0.0
0.1
0.2
0.3
0.4
N = 100000 Bandwidth = 0.005737

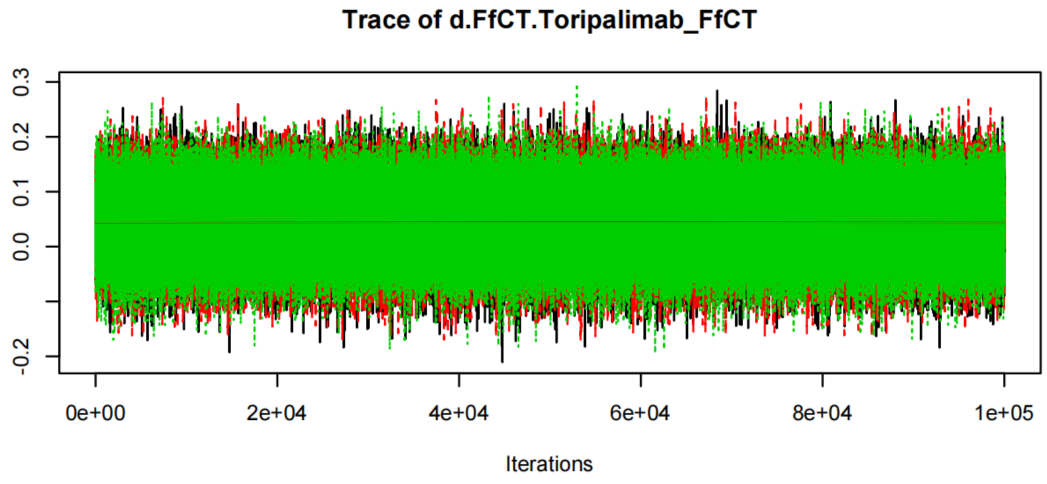
Trace of d.FfCT.Toripalimab_FfCT
-0.2
0.0
0.1
0.2
0.3
0e+00
2e+04
4e+04
6e+04
8e+04
1e+05
Iterations

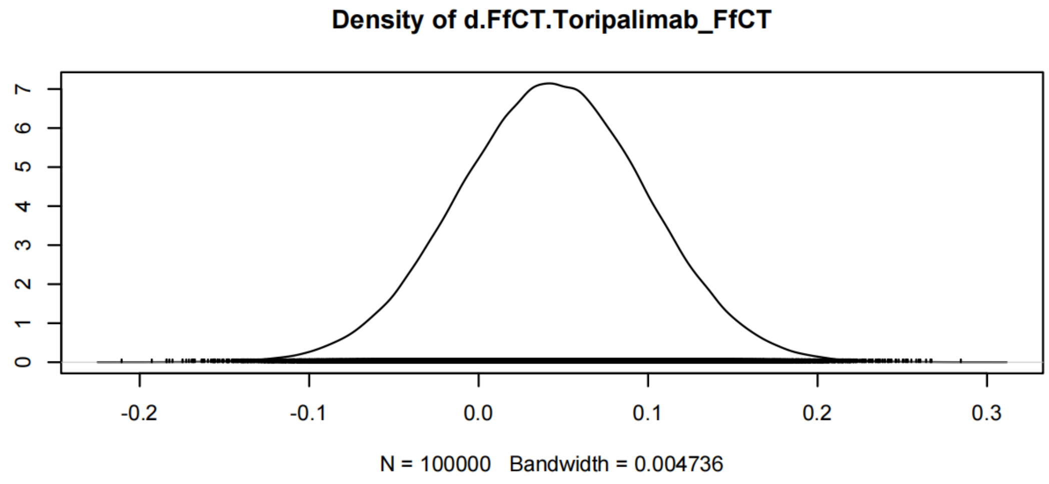
Density of d.FfCT.Toripalimab_FfCT
0
1
2
3
4
5
6
7
-0.2
-0.1
0.0
0.1
0.2
0.3
N = 100000 Bandwidth = 0.004736
